# Supplementary material for: Immune activation of Bio-Germanium in a randomized, double-blind, placebo-controlled clinical trial with 130 human subjects: Therapeutic opportunities from new insights
Source: PLoS One. 2020 Oct 19;15(10):e0240358. doi: 10.1371/journal.pone.0240358 (PMC7572073; doi:10.1371/journal.pone.0240358)
Supplement: S1 File — (PDF) [file pone.0240358.s001.pdf]

Supporting Information

S1 Table. NK Cell Cytotoxicity and IgG1 Values at Baseline and Follow-up among the Female Subjects. Subgroup analysis using only female subjects also resulted in statistically significant *P*-values in all NK cell cytotoxicity assay E:T ratios, and the findings are consistent with the findings in the entire population.

(%)	Placebo Group (*n* =58)		Bio-Germanium Group (*n* =57)				
	Baseline	Follow-up	Baseline	Follow-up	P^a	P^b	P^c
E:T = 50:1	49.30 ± 25.74	44.68 ± 24.78	42.34 ± 28.57	55.94 ± 27.08**	0.101	0.017	
Δ	*-4.62 ± 26.98*		*13.61 ± 30.97*				*0.002*
E:T = 10:1	28.79 ± 17.11	23.61 ± 18.26*	22.78 ± 14.80	33.73 ± 18.27**	0.018	<0.001	
Δ	*-5.19 ± 17.78*		*10.95 ± 23.04*				*<0.001*
E:T = 5:1	19.95 ± 13.44	13.71 ± 9.78**	13.64 ± 10.47	23.87 ± 15.47***	0.008	<0.001	
Δ	*-6.31 ± 16.37*		*10.16 ± 16.97*				*<0.001*
E:T = 2.5:1	14.17 ± 16.08	8.69 ± 8.73*	9.58 ± 10.03	16.16 ± 12.96**	0.025	<0.001	
Δ	*-5.47 ± 17.65*		*6.57 ± 15.70*				*<0.001*
IgG1	8223.45 ± 1551.66	8026.90 ± 1404.68**	8052.63 ± 1518.01	8038.42 ± 1641.09	0.435	0.825	
Δ	*-196.55 ± 515.64*		*-14.21 ± 478.15*				*0.063*

The data represent the mean ± standard deviation.
Δ represents the change from baseline at follow-up.
P^a-values were derived from the Wilcoxon rank-sum test at baseline between groups.
P^b-values were derived from the Wilcoxon rank-sum test at follow-up between groups.
P^c-values were derived from adjusted baseline ANCOVA for Δ between groups.
$^{*}P<0.05$, $^{**}P<0.01$ and $^{***}P<0.001$ values were derived from a paired *t*-test at follow-up from baseline within groups.

S2 Table. NK Cell Cytotoxicity and IgG1 Values at Baseline and Follow-up among the Postmenopausal Female Subjects. Subgroup analysis using postmenopausal female subjects also resulted in statistically significant *P*-values in most NK cell cytotoxicity assay E:T ratios and IgG1, which is consistent with the earlier findings.

(%)	Placebo Group (*n* =34)		Bio-Germanium Group (*n* =39)				
	Baseline	Follow-up	Baseline	Follow-up	P^a	P^b	P^c
E:T = 50:1	48.17 ± 26.82	42.45 ± 29.81	48.51 ± 26.82	56.35 ± 29.67 *	0.243	0.274	
Δ	*0.34 ± 29.21*		*13.90 ± 32.15*				*0.102*
E:T = 10:1	27.04 ± 16.60	21.69 ± 19.98	24.59 ± 14.57	34.88 ± 18.25**	0.048	0.009	
Δ	*-2.45 ± 18.09*		*13.19 ± 24.90*				*0.005*
E:T = 5:1	17.73 ± 15.25	12.10 ± 9.30	14.22 ± 10.40	24.27 ± 16.57**	0.165	0.001	
Δ	*-3.51 ± 18.05*		*12.17 ± 17.33*				*0.002*
E:T = 2.5:1	14.31 ± 20.19	7.43 ± 5.89	9.93 ± 8.69	15.84 ± 13.74**	0.070	0.054	
Δ	*-4.37 ± 21.05*		*8.40 ± 14.59*				*0.029*
IgG1	8130.88 ± 1480.87	7887.94 ± 1427.34*	7951.03 ± 1580.04	7950.77 ± 1785.22	0.465	0.821	
Δ	*-242.94 ± 448.93*		*-0.26 ± 496.41*				*0.032*

The data represent the mean ± standard deviation.
Δ represents the change from baseline at follow-up.
P^a-values were derived from the Wilcoxon rank-sum test at baseline between groups.
P^b-values were derived from the Wilcoxon rank-sum test at follow-up between groups.
P^c-values were derived from adjusted baseline ANCOVA for Δ between groups.
$^*P<0.05$ and $^{**}P<0.01$ values were derived from a paired *t*-test at follow-up from baseline within groups.

S3 Table. NK Cell Cytotoxicity and IgG1 Values at Baseline and Follow-up among the Premenopausal Female Subjects. Subgroup analysis of premenopausal female subjects also resulted in statistically significant *P*-values in all NK cell cytotoxicity assay E:T ratios, which is consistent with the earlier findings.

(%)	Placebo Group (*n* =24)		Bio-Germanium Group (*n* =18)				
	Baseline	Follow-up	Baseline	Follow-up	P^a	P^b	P^c
E:T = 50:1	50.91 ± 24.60	39.26 ± 20.91*	42.10 ± 26.53	55.07 ± 21.13	0.286	0.017	
Δ	*-11.65 ± 22.18*		*12.97 ± 29.13*				*0.004*
E:T = 10:1	31.27 ± 17.87	22.22 ± 15.33*	25.16 ± 14.05	31.24 ± 18.58	0.127	0.042	
Δ	*-9.06 ± 16.94*		*6.08 ± 18.08*				*0.019*
E:T = 5:1	23.10 ± 9.81	12.83 ± 8.98**	17.18 ± 12.22	23.01 ± 13.18	0.053	0.007	
Δ	*-10.27 ± 13.01*		*5.83 ± 15.75*				*0.003*
E:T = 2.5:1	13.96 ± 7.49	6.93 ± 8.65**	14.24 ± 14.83	16.85 ± 11.43	0.799	0.002	
Δ	*-7.03 ± 11.50*		*2.61 ± 17.67*				*0.003*
IgG1	8354.58 ± 1670.24	8223.75 ± 1377.62	8272.78 ± 1391.16	8228.33 ± 1300.73	0.929	0.839	
Δ	*-130.83 ± 601.74*		*-44.44 ± 448.22*				*0.625*

The data represent the mean ± standard deviation.
Δ represents the change from baseline at follow-up.
P^a-values were derived from the Wilcoxon rank-sum test at baseline between groups.
P^b-values were derived from the Wilcoxon rank-sum test at follow-up between groups.
P^c-values were derived from adjusted baseline ANCOVA for Δ between groups.
$^{*}P<0.05$ and $^{**}P<0.01$ values were derived from a paired *t*-test at follow-up from baseline within groups.

S4 Fig. Line Graphs of the NK Cell Cytotoxicity and IgG1 Values at Baseline and Follow-up in the Placebo Group.

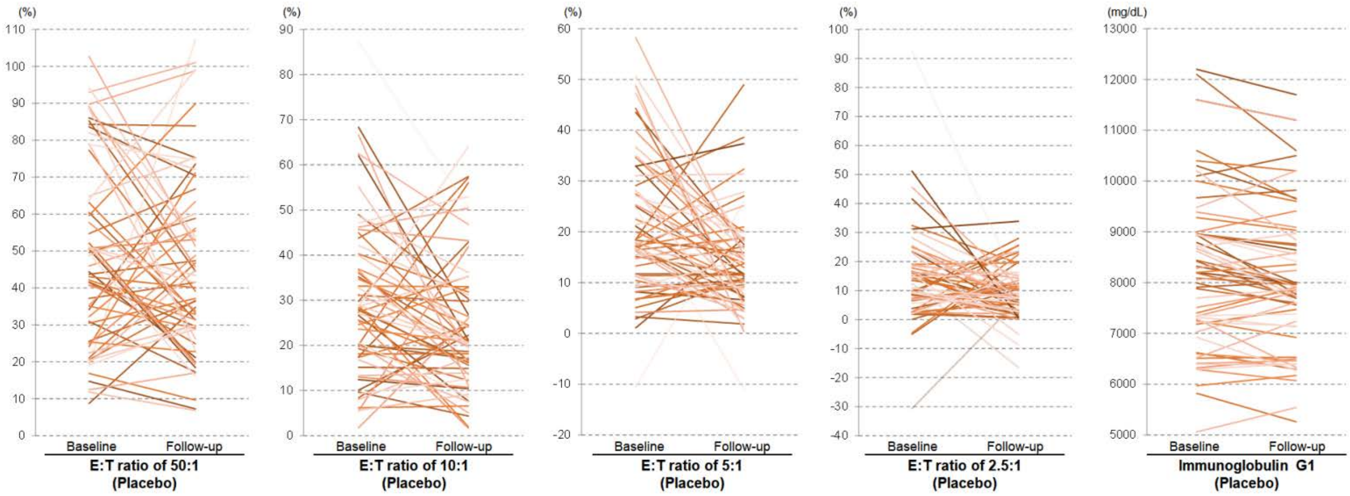

S5 Fig. Line Graphs of NK Cell Cytotoxicity and IgG1 Values at Baseline and Follow-up in the Bio-Germanium Group.

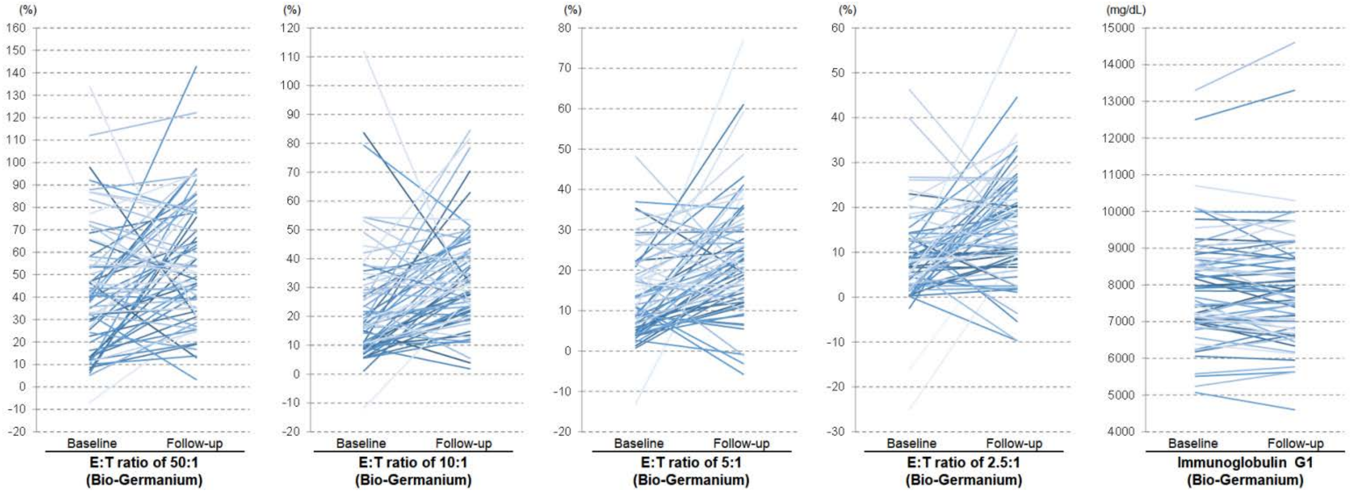

S6 Appendix. Determination of the Group Size and its Calculation.

Our group size was calculated by referring to an earlier clinical trial [61] that showed statistically significant results in NK cell cytotoxic activities. As we set NK cells as our primary biomarker, we used the results from the reference study in the calculation of our sample size.

The values and estimates using the results reported in the reference study are as follows:

Biomarker	Subject (n)		△ Mean (baseline to follow-up)		Standard Deviation (SD)		Common Standard Deviation (S)
	Placebo	Test	Placebo	Test	Placebo	Test	
Cytotoxicity of NK Cells (10:1)	31	31	-0.28	7.4	10.7	10.0	10.7

- Based on a statistical test power of 80% and significance level of 0.05
- As the SD of the difference between the test and placebo groups before and after their supplementation was not provided, the correlation coefficient between the before and after supplementation data was estimated to be 0.25
- A higher SD value of 10.7 was selected and used as S

The following assumptions are applied to estimate the sample size of participants in our trial:

1) Based on the superiority test
2) Significance level (α) of 0.05 using a two-sided test
3) Type 2 error (β) set to 0.2, and the power of the test maintained at 80%
4) A 1:1 allocation ratio in the test and placebo groups
5) Use of data from a clinical trial showing statistically significant NK cell cytotoxicity results. Based on its clinical relevance to our study, a 75% adjustment is applied to the difference in deltas, yielding an effect size (d) of 5.76 (= 7.68 × 75%)

To validate the above, the following hypotheses are set:

H0 : $\mu t = \mu c$ (the mean NK cell activity in the two groups is the same at follow-up)
H1 : $\mu t \neq \mu c$ (the mean NK cell activity in the two groups is not the same at follow-up)

Assuming 1)-5) above, the sample size calculated for our study is as follows (a two-sided test):

$$n_t = \frac{(Z_{\alpha/2}+Z_{\beta})^2 \times S^2 \times 2}{d^2}$$

where

$Z_{\alpha/2}$: Threshold or fractile that the right tail area is $\alpha/2$ in a standard normal distribution
Z_{β} : Threshold or fractile that the right tail area is β in a standard normal distribution
α : Size of Type 1 error
β : Size of Type 2 error

σ	:	Standard deviation
d	:	Effect size of the difference in Δs between the test and placebo groups following adjustment based on clinical relevance

Using the above, the minimum sample size per group is calculated as 55.

$$55 = \frac{(1.96 + 0.84)^2 \times 10.7^2 \times 2}{5.76^2}$$

Based on the reference study's NK cell cytotoxicity data, our sample size per group is set to 55 participants. Assuming a dropout ratio of 15% during the recruiting and screening process, the target sample size of participants for registration is 65 per group, which is equivalent to 130 total participants.
