# Supplementary material for: Immune activation of Bio-Germanium in a randomized, double-blind, placebo-controlled clinical trial with 130 human subjects: Therapeutic opportunities from new insights
Source: PLoS One. 2020 Oct 19;15(10):e0240358. doi: 10.1371/journal.pone.0240358 (PMC7572073; doi:10.1371/journal.pone.0240358)
Supplement: S2 File — (PDF) [file pone.0240358.s002.pdf]

Full Version in Korean

1. **Trial Study Protocol for IRB's Review**
2. Case Report Form Designed for Clinical Trial
3. Written Explanation to Participants
4. Consent Form for Participation
5. Consent Form for Human Biologic Materials
6. Survey for Stress Response Inventory (SRI)
7. Survey for Nutrition Intake
8. Self-Assessment Form
9. Advertisement for Recruitment

심의용 연구 계획서

(인간대상 연구용)

연구제목: 바이오게르마늄의 면역기능증진 효과 및 면역세포 활성화 효과 평가를 위한 인체적용시험

연세대학교
생활과학대학 식품영양학과
이 종 호

1. 연구배경

최근 들어 SARS나 조류독감 등과 같은 질병들로 인해 면역 기능 증진 관련 식품에 대한 관심도가 증대되었으며, 식생활의 향상과 의학의 발달로 인간의 평균 수명이 연장되고 문명의 발달에 따른 환경오염, 스트레스 및 운동부족이 원인이 되어 이전보다 더 심각한 병을 가진 채 장기간 생존하는 사람들이 많아지면서 면역력 저하 인구가 급증하고 있다.

이에 최근 천연물질 중 암 예방 성분이나 생리 활성 조절물질 및 식품 중 기능성 성분을 찾아내어 이를 건강의 유지와 증진을 위해 활용하고자 하는 연구가 활발히 진행되고 있으며, 이 중 식품 내에 존재하는 성분들의 단순한 영양소 역할 외에 기능성 물질로서의 역할에 대한 관심이 높아지고 있다. 이러한 추세에 부응하여 천연 식물 자원을 대상으로 노화방지, 성인병 예방, 면역 증강 효과 등에 대한 연구와 항체 생성 및 항산화 효과와 같은 각종 생리활성을 나타내는 물질에 대한 연구가 활발히 진행되고 있다.

게르마늄이라는 원소는 1886년 독일의 화학자 Clemen Winkler에 의해 최초로 발견되었으며 의학적인 효능이 처음 발견된 것은 1930년 프랑스와 스페인의 국경지방인 Lourdes의 샘물이 여러 가지 질병에 큰 효과가 있다고 알려져 샘물의 성분 분석 결과 게르마늄의 함량이 높아 치료 효과를 보인다는 보고서가 발표된 이후부터다. 여러 연구를 통해 토양, 물, 식물 그리고 동물 체내에 자연적으로 발견되는 미량 원소인 게르마늄이 항암, 항염증 및 면역 증강 기능 등 생리학적인 효능이 알려져 있다[1-11].

본 연구의 원료인 바이오게르마늄을 비롯한 합성 유기게르마늄 Ge-132, spirogermanium 등은 여러 연구에서 INF, TNF-α를 매개로 면역체계의 가장 중추적 역할을 하는 대식세포와 자연살해세포의 생성과 활성을 증가시킨다고 보고하고 있으며, 종양괴사인자인 TNF-α을 유의적 증가시켰다[12-18]. 또한 면역글로불린(Immunoglobulin)과 싸이토카인(Cytokine), 림포카인(Lymphokine)를 유의적으로 증가시키고[19] B 림프구와 T 림프구의 활성을 강화시키는[12-13), 16] 것으로 나타나 바이오게르마늄은 선천성 면역체계 및 후천성 면역체계 등의 각 면역세포들이 상호 협조하여 효과적인 면역체계를 이루어내는 것으로 사료된다.

따라서 본 연구는 일반인을 대상으로 이전 인체적용시험연구와 동일한 원료인 바이오게르마늄 효모(2,800~3,300ppm)를 섭취하였을 때, 면역관련 지표인 WBC 수, NK cell activity, IFN-ɣ, TNF-α, IgG1, IgG2, IgM 그리고 IL-2, 6, 12의 면역관련 지표들의 개선효과를 통해 면역 기능 증진 및 면역세포 활성화 효과를 확인하고자 한다.

1.1. 전임상시험 및 임상시험 결과

1.1.1. 독성시험 결과

년도	연구명 및 연구소	요약

1994	급성 독성시험<br>(충북대)	ㆍ목적: 게란티 바이오게르마늄의 급성독성여부시험<br>ㆍ결론: ICR 마우스를 대상으로 게란티 바이오게르마늄을 5,000mg/kg의 용량으로 투여 시 사망률 임상증상 체중변화 임상 병리 조직학적 독성 등이 발견되지 않았음.
1996	급성 독성시험<br>(Biological Test Center, 미국)	ㆍ목적: 게란티 바이오게르마늄의 급성독성여부시험미국 (실험업체에 문의)<br>ㆍ결론: ICR 마우스를 대상으로 바이오게르마늄을 5,000mg/kg의 용량으로 투여 시 독성의 발현이 없었으며 장기의 손상이나 병리학적 이상 또한 발견되지 않았음.
2001	만성 독성시험<br>(강원대)	ㆍ목적: 게란티 바이오게르마늄의 급성독성여부시험<br>ㆍ결론: 랫트와 비글견을 대상으로 바이오게르마늄을 3,000mg/kg 및,000mg/kg의 용량으로 0개월간 투여시 독성발현 없고 해부학적 병리학적 혈액학적 등의 이상증후 없었음
2001	유기태 및 N-terminal 확인을 위한 신물질 분리 정제 실험<br>(서울대 NICEM)	ㆍ목적: 게란티 바이오게르마늄의 유기형태여부확인<br>ㆍ결과: 1년 6개월 간 게란티 바이오게르마늄을 분리정제하여 계측한 결과 N-terminal을 확인하고, 에놀라아제 등의 펩타이드와 결합되어있는 유기형태의 게르마늄임을 확인함.
2002	단회투여 독성시험 I<br>(바이오톡스텍)	ㆍ목적: 게란티 바이오게르마늄의 급성독성여부시험<br>ㆍ결과: 랫트를 대상으로 바이오게르마늄을 2,000mg/kg의 용량으로 투여 시 사망사례, 일반병리증상 체중변화 조직병리학적 등의 이상이나 독성이 관찰되지 않았음.
2002	단회투여 독성시험 II<br>(바이오톡스텍)	ㆍ목적: 게란티 바이오게르마늄의 소핵 형성여부시험<br>ㆍ결론: ICR 마우스의 골수세포를 이용하여 소핵시험을 실시한 결과 게란티 바이오게르마늄(최대농도,000 mg/kg)에 의한 조혈기능 억제 등의 세포독성은 관찰되지 않았으며 마우스 골수세포의 소핵 형성에 영향을 주지않는 것으로 사료됨.
2002	반복투여 독성시험 I<br>(바이오톡스텍)	ㆍ목적: 게란티 바이오게르마늄의 만성 독성 여부 시험.<br>ㆍ결론: 랫트를 대상으로 게란티 바이오게르마늄을 최대 2,000mg/kg의 용량으로 13주간 투여 시, 사망사례, 체중 변화, 안과 및 뇨 검사, 혈액학적, 혈액생화학적 검사 및 부검 등에서 이상이나 독성이 관찰되지 않았으며, 무해용량(NOEL)은 2,000mg/kg로 사료됨.
2002	반복투여 독성시험 II<br>(바이오톡스텍)	ㆍ목적: 게란티 바이오게르마늄의 만성 독성 여부 시험.<br>ㆍ결론: 비글견을 대상으로 게란티 바이오게르마늄을 최대 2,000mg/kg의 용량으로 13주간 투여 시, 사망사례, 체중 변화, 안과 및 뇨 검사, 혈액학적, 혈액생화학

		적 검사 및 부검 등에서 이상이나 독성이 관찰되지 않았으며, 무해용량(NOEL)은 2,000mg/kg로 사료됨.
2003	유전 독성시험:<br>복귀돌연변이 시험<br>(바이오톡스텍)	・목적: 게란티 바이오게르마늄의 복귀돌연변이 유발성 여부 시험.<br>・결론: Salmonella Typhimurium 및 Echerichia Coli 균주를 이용한 복귀돌연변이 유발 가능성의 실험 결과, 게란티 바이오게르마늄(최대 농도 5,000 ㎍/plate)은 복귀돌연변이를 유발할 가능 성이 없는 것으로 사료됨.
2003	유전 독성시험:<br>염색체이상 시험<br>(바이오톡스텍)	・목적: 게란티 바이오게르마늄의 염색체이상 유발성 여부 시험.<br>・결론: CHL (Chinese Hamster Lung) 배양세포를 이용한 염색체이상 유발 여부의 시험 결과, 대사활성계의 적용여부에 관계없이 게란티 바이오게르마늄(최대 농도 5,000 ㎍/plate)은 CHL 세포에 대해 구조적 또는 숫적 이상의 염색체 이상을 유발하지 않는 것으로 사료됨.
2003	유전 독성시험:<br>소핵 시험<br>(바이오톡스텍)	・목적: 게란티 바이오게르마늄의 소핵 형성 여부 시험.<br>・결론: ICR 마우스의 골수세포를 이용하여 소핵시험을 실시한 결과, 게란티 바이오게르마늄(최대 농도 2,000 mg/kg)에 의한 조혈기능 억제 등의 세포독성은 관찰되지 않았으며, 마우스 골수세포의 소핵 형성에 영향을 주지 않는 것으로 사료됨.
2004	신장 독성 분석 실험<br>(한국화학시험연구원)	・목적: 게란티 바이오게르마늄의 신장잔류여부확인<br>・결론: 13주 반복투여 독성실험에서 게란티 바이오게르마늄을 투여한 모든 그룹의 랫트의 신장을 적출하여 게르마늄의 잔류여부를 측정결과 게란티 바이오게르마늄은 신장에 잔류되지 않았음을 확인함.
2003~<br>2005	인공위액(SGF)에<br>의한 해리 여부를<br>통한 유기게르마늄<br>확인 시험<br>(중앙대 핵자기공명<br>연구실)	・목적: 게란티 바이오게르마늄의 유기형태 여부 확인<br>・결론: 2003년과 2005년 2회 실시한 실험에서, 게란티 바이오게르마늄은 인공위액(SGF)에서 무기 형태 혹은 이온 형태의 게르마늄으로 해리되지 않고 견고한 유기형태로 유지되고 있음을 확인함.
2005	제조 및 이의<br>게르마늄 결합에<br>관한 연구<br>(중앙대/게란티제약<br>중앙연구소	・목적: 게란티 바이오게르마늄 내의게르마늄 결합 상태 확인<br>・결론: 게란티 바이오게르마늄 내에서 단백질과 결합하고 있는 게르마늄이 인공위액에서 해리되는 지 알아본 결과, 투석막을 이용한 투석 전후에 따른 게르마늄 총량이 유의적인 차이가 나타나지 않음을 확인함. 이는 인공 위액에서 바이오게르마늄이 무기형태 혹은 이온형태의 게르마늄으로 쉽게 해리되지 않는 것으로 사료됨.
2007	바이오게르마늄 내의	・목적: 게란티 바이오게르마늄의 유기형태 여부확인

	GeO_2 정성분석<br>(전남대)	ㆍ결론: 정량 및 정성분석 시험과 NMR 시험을 병행하여 게란티 바이오게르마늄 내에 존재하는 게르마늄은 단백질 올리고펩타이드 아미노산 등과 결합된 유기게르마늄이며, 무기게르마늄이 잔존하지 않음을 확인함.

1.1.2. 시험관 시험 결과

시험물질	시험대상	농도	바이오마커	결과
신청원료 바이오게르마늄 효모	-	-	1.복강삼출세포 변화측정<br>2.복강대식세포 활성능 검사: Rosette 형성능 및 탐식능 측정<br>3. 주화능<br>4. 부착능<br>5. 활성산소 생성능<br>6. 항체 생성능	전반적으로 면역세포의 활성화에 유의성 있는 효과를 관찰하여 면역증진에 유용성이 있을 가능성을 확인함[20].
신청원료 바이오게르마늄 효모	-	-	1. NO<br>2. TNF-α	바이오게르마늄 효모는 NO생성에 있어 양성대조물질 (Lipopolysaccharide) 보다 NO생성 결과가 낮았으나, TNF-α 의 생성은 양성대조물질과 동등한 결과를 얻어 면역기능 활성의 가능성을 관찰함[21].
바이오게르마늄 효모 복합물	C57BL/6 마우스의 비장으로 분리된 자연살해세포	1, 10 및 100 ㎍/ml	NK cell cytotoxicity	게란티-골드는 100㎍/ml에서 높은 활성을 나타내었지만, 1 및 10㎍/ml에서는 활성을 나타내지 못 함. 이를 통하여, 일정량 이상의 농도(100㎍/ml 이상)가 되어야 자연살해세포의 활성을 증가시킬 수 있다는 것으로 판단됨[22].
신청원료 바이오게르마늄 효모	대식세포 Raw 264.7	0, 5, 10, 25, 50, 100, 200 ㎍/ml	1. 세포생존율<br>2. iNOS 발현<br>3. TNF-α 생성	1. 세포생존율(%)과 NO의 생성량은 10㎍/ml의 처리 농도에서 유의적으로 증가함 (vs C, $p<0.05$)<br>2. 면역 및 항암 조절인자로 알려진 TNF-α 생성이 10㎍/ml 의 농도에서 유의적

				으로 증가 하였으며 (vs C, $p<0.05$), 농도 의존적으로 증가함[23].
신청원료 바이오게르마늄 효모	-	-	1. 비장 세포수<br>2. T림프구<br>3. 대식세포<br>4. 수상돌기 세포수<br>5. B림프구<br>6. 증식(MTT assay)<br>7. LDH assay	바이오게르마늄 효모는 NO 생성에 있어 양성대조물질 (Lipopolysaccharide) 보다 NO생성 결과가 낮았으나, TNF-α 의 생성은 양성대조물질과 동등한 결과를 얻어 면역기능 활성의 가능성을 관찰함[24].

1.1.3. 동물 시험 결과

시험물질	시험대상	농도	바이오마커	결과
신청원료 바이오게르마늄 효모	S-180 복수암 유발 ICR마우스	200 및 2,000 mg/kg	복수암에 대한 종양억제 효과 측정:<br>1. 생존일수중앙치 및 연명률<br>2. 복수암의 진행에 따른 체중증가	바이오게르마늄 투여군은 대조군의 14.9일 보다 18.2% 높은 17.6일 생존일수평균치를 기록하여 연명효과에 유의성이 있음을 관찰함[25].
	S-180 cell을 이식하여, 고형암을 유발 (양성대조군은 정맥투여)	600, 1,200 및 2,400 mg/kg	고형암에 대한 종양억제 효과 측정:<br>체중측정, 종양무게, 비장무게, 고형암 형성 억제율 확인	1. 종양세포 저해율은 양성 대조군에서 51.55%로 측정 되었으나, 시험물질 2,400 mg/kg 투여군에서는 63.04%의 저해율을 나타냄.<br>2. 종양중량은 시험물질 농도에 반비례하게 감소하였으며 저해율은 농도의존적으로 증가하는 것을 확인 함[26].
	-	-	1. 항체생성능<br>2. Plaque-forming cell assay	Plaque-forming cell assay를 실시한 결과, 일정 농도 이상에서 비장 세포당 플라그(plaque)를 형성한 세포의 수가 양성대조군과 동일하거나 더 높은 증가를 보였음으로 바이오게르마늄은 체액성 면역계를 활성화 한다고 판단됨[27].
	female	100,	1. 체중변화	1. 바이오게르마늄 효모섭취로

	balb/cmice	200, 400, 800 mg/kg / 4주	2. 면역장기의 변화 여부: 비장의 크기 변화, 세포수, 길이, 무게 측정<br>3. 비장의 세포 분화 및 세포독성 여부 확인<br>4. T-cell로 부터 IL-2 및 IFN-ɣ의 생성능 확인<br>5. 플라크 형성 확인: T-의존성항원인 면양적혈구(Sheep Red Blood Cells,v SRBC)에 대한 항체(IgM) 생성능의 영향 확인<br>6. 면역 관련 세포: T림프구, B림프구, 대식세포, 수상돌기세포 수의 변화 확인	인한 부작용은 없었음.<br>2. 바이오게르마늄 효모100, 200, 400, mg/kg 을 투여한 군에서 비장 세포수가 증가하였으나, 비장의 길이, 무게에서 군간 유의적 차이는 나타나지 않음.<br>3. 바이오게르마늄 효모100, 200, 400, 800 mg/kg을 투여한 군에서 B림프구가 대조군과 비교하여 유의적으로 증식하였으며 (vs C, $p<0.05$), 농도 의존적으로 증가함.<br>4. 플라크 형성은 바이오게르마늄 효모200 mg/kg이상에서 유의적으로 증가하였으며(vs C, $p<0.05$), 농도의존적으로 증가함. 이를 통하여, 바이오게르마늄 효모200 mg/kg이상에서 면역 증진 효과가 있을 것으로 사료됨.<br>5. B-cell, T-cell에 대한 Cell specific 세포독성여부 확인 결과, 바이오게르마늄 효모800 mg/kg 투여군에서 비교적 세포독성이 낮은 결과를 나타냈으며, T-cell의 경우 음성대조군과 비교하여 유의적인 결과를 보여(vs NC, $p<0.05$) T-cell에 대한 세포 보호 효과가 인정됨[28].

1.1.4. 인체적용시험 결과

○ 50-75세의 연구대상자 50명 중 시험군과 대조군에게 바이오게르마늄 효모와 corn

starch를 각각 400mg 경질 캡슐에 충진하여 1일 3캡슐 1.2g을 2개월간 복용시켜 섭취 전, 후 비교분석하였다. 혈청 지질의 변화 중 바이오게르마늄 효모 섭취군의 중성지방만이 유의적으로 저하되었으며 면역세포의 변화에서는 바이오게르마늄 효모 섭취군의 B세포만이 유일하게 증가하였고 TNF-α도 바이오게르마늄 효모 섭취군에서 보충 전보다 보충 후에 유의하게 증가하였다. 따라서 바이오게르마늄 효모는 세포의 면역 반응을 개선하고 다양한 만성질환으로부터 조직을 보호 하는데 유용할 것이라고 사료된다[29].

○ Ge-132의 섭취가 암환자의 말초 혈액 림프구의 자연살해세포 활성에 미치는 영향을 조사한 실험에서 Ge-132 1000mg/day의 지속적인 경구 투여는 투여 전 수치가 낮은 4명에게서 활성 증가가 나타났으나 투여 전 수치가 비교적 높은 2명에게서는 증강 효과가 나타나지 않았다. Ge-132의 간헐적인 투여에서 7명 모두 활성이 증가하였으며, 그 중 4명은 측정 3, 10일 째에서 큰 변화가 나타나지 않았다. 또한 투여 전의 수치를 밑도는 것은 한 명도 확인되지 않았다. Ge-132는 간헐적인 투여가 더 효과적인 것으로 판단되며, NK 유도인자인 INF의 빈번한 투여는 NK의 활성 저하를 보이는 경우가 있다고 사료된다[30].

○ IFN의 효과 및 호중성백혈구 기능에서 IFN의 유도인자를 측정하기 위해 IFN 1일 3.0×10(6) units을 근육 내로 투여하거나 Ge-132 1일 2회, 250mg을 섭취시킨 12명의 종양 환자에게서 neutrophil ChL(chemiluminescence)를 측정하였다. 그 결과 처리 일주일 후 측정된 neutrophil ChL의 최고 농도는 처리전과 비교 시 증가하였으나 처리 한 달 후에는 매일 시험물질을 섭취하였음에도 불구하고 처리 전에 비해 감소되었다. IFN와 Ge-132는 생체 내에서 방어반응 초기에 출연하는 호중구 기능을 활성화하여 폭넓은 방어 기구의 활성화에 관여한다고 사료된다[31].

○ 바이오게르마늄 효모의 게르마늄 결합상태를 확인한 실험에서 효모 균체 내에 유입된 게르마늄이 인공 위액에서 무기 형태 혹은 이온 형태로 쉽게 해리되지 않는 것으로 나타나 본 원료에 함유된 최종 게르마늄의 형태가 무독성의 유기 게르마늄임이 확인 되었다[32-34].

○ 바이오게르마늄 효모를 대상으로 실시한 단회투여독성시험 (설치류, 비설치류), 3개월 이상 반복투여독성시험(설치류, 비설치류), 유전독성시험(복귀돌연변이시험, 염색체 이상시험, 소핵시험)을 실시한 결과 시험물질과 관련된 사망과 이상반응은 발생하지 않았고 무독성량은 2,000mg/kg이상이며 돌연변이, 염색체 이상 가능성이 없는 것으로 나타났다[35-42].

○ 10개월간의 반복경구독성시험결과(설치류, 비설치류)에서도 독성과 관련된 이상반응은 나타나지 않았으며 게르마늄의 섭취는 간과 신장에서 축적되지 않는다[43].

○ 사람에게 바이오게르마늄 효모 1.2g을 2개월 간 섭취시킨 결과에서도 혈액학적 및 혈액화학적 검사에서 이상반응이나 부작용은 발생하지 않았다[29].

○ 합성 유기 게르마늄인 Ge-132의 경우 섭취한 후 24시간이내에 대사적인 변이 없이 100% 배설된다고 조사되었다[44].

○ 바이오게르마늄 효모를 1.2g(게르마늄 3.6mg)섭취시킨 인체시험[29]에서 1일 게르마늄 함량은 외국에서 판매중인 Ge-132의 순수게르마늄 1일 섭취량인 12.8~192.6mg에 비해 소량이다. (표 2. 외국에서 판매되는 게르마늄 보충용 제품의 게르마늄 형태 및 섭취량)

표 3. 외국에서 판매되는 게르마늄 보충용 제품의 게르마늄 형태 및 섭취량

제품명	게르마늄형태	제품설명	일일 Ge-132 섭취량	순수 게르마늄 함유량
Jarrow Formulas:	organic germanium(Ge-132)	전제품 일일 1~3정 복용 (Ge-132 30mg, 100mg, 150mg/캡슐)	30mg~450mg	12.8mg~192.6mg
NUTRICOLOGY /Allergy Research	Ge-132	캡슐제품:<br>Ge-132 150mg/캡슐<br>: 일일 한 알씩 1~5회 복용<br>Ge-132 100mg/캡슐<br>: 일일 1~2알씩 1~4회 복용<br>파우더제품<br>: 일일 Ge-132 34~104mg 복용	34~750mg	14,6mg~321mg
1001herbs	Bis-carboxyethyl germanium sesquioxide	Ge-132 30mg/캡슐<br>: 일일 1~3캡슐 복용	30mg~90mg	12.8mg~38.5mg

○ 이상의 결과를 토대로 신청 원료인 바이오게르마늄 효모의 게르마늄은 무독성인 유기게르마늄 형태로 존재하며, 기본독성시험과 본 품목의 기능성이 발현되는 용량을 사람이 섭취시에도 이상 반응이나 부작용은 발생하지 않았고 유기게르마늄이 간이나 신장에 축적되지 않는 것으로 나타나 안전성에는 문제가 없는 것으로 판단된다.

2. 연구목적

본 연구는 일반인을 대상으로 바이오게르마늄 효모를 섭취하였을 때 대조식품과 비교하여 면역기능 증진 및 면역세포 활성화에 미치는 유효성을 평가하기 위하여 계획되었다.

3. 연구 실시 기관명 및 주소

연세대학교 생활과학대학 식품영양학과 (서울시 서대문구 연세로 50)

4. 연구 지원기관

게란티제약㈜ (서울 강남구 테헤란로 7길 22 과학기술회관 신관 408호)

4.1. 연구 수탁기관 *(CRO)*

네오뉴트라㈜ (서울시 종로구 대학로44 우일빌딩 4층)

5. 연구책임자, 공동연구자, 참여연구원, 과제담당자의 성명과 직명

	성명	직명
연구책임자	이 종 호	연세대학교 생활과학대학 식품영양학과 교수
연구담당자	조 정 민	연세대학교 생활과학대학 식품영양학과 박사과정
참여연구원	신 동 엽	세브란스병원 내분비내과 임상교수
	문 민 정	연세대학교 생활과학대학 식품영양학과 석사과정
	한 영 민	연세대학교 생활과학대학 식품영양학과 석사과정

6. 연구기간

IRB 승인일로부터 2019년 03월 31일

7. 연구대상자

7.1. 선정기준

다음 기술된 조건에 모두 부합되는 사람을 연구대상자로 선정한다.

1) 만 25세 이상 ~ 만 75세 이하의 남, 여
2) 스크리닝 검사에서 측정한 말초혈액 백혈구 수치가 $4X10^{3}$cells/ul 이상~$8X10^{3}$cells/ul 미만인 자
3) 본 연구에 참여할 것을 동의하고 서면 동의서에 연구대상자(또는 법정 대리인)가 자의로 서명한 자

7.2. 제외기준

다음 기술된 조건에 하나라도 해당되는 사람은 연구대상자에서 제외한다.

1) 임상적으로 유의한 급성 또는 만성 심뇌혈관계, 면역계, 호흡기계, 간담도계, 신장 및 비뇨기계, 신경계, 근골격계 질환, 정신성, 감염성 및 혈액·종양성 질환 등으로 현재 치료 중인 자 (단, 대상자의 상태를 고려하여 연구책임자의 판단에 따라 연구에 참여 가능하다.
2) 조절되지 않는 고혈압이 있는 자 (140/90mmHg 이상, 연구대상자 10분 안정 후 측정

기준)

3) 조절되지 않는 당뇨병 환자 (공복혈당 126mg/dL 이상 또는 3개월 이내에 당뇨로 인해 약제를 새로 시작하는 경우)
4) 스크리닝 전 3개월 이내에 예방접종을 실시한 자
5) AST(GOT) 또는 ALT(GPT) 혈중치가 120 IU/L 초과한 자
6) Creatinine 혈중치가 남자 2.4 mg/dL, 여자 1.8 mg/dL 초과한 자
7) 스크리닝 전 2주 이내에 면역에 영향을 미칠 수 있는 건강기능식품을 섭취했거나 섭취 중인 자
8) 속쓰림, 소화불량 등의 심한 위장관 증상을 호소하는 자
9) 임신, 수유 중이거나 본 연구 기간 동안 임신 계획이 있는 자
10) 시험 식품 관련 식품에 민감하거나 알레르기가 있는 자
11) 본 연구 중에 다른 연구에 참가할 계획이 있는 자
12) 본 연구 시작 4주 이내에 다른 연구에 참여했던 자
13) 연구자가 본 연구에 부적합하다고 판단하는 자

7.3. 이중눈가림의 유지

이중눈가림 유지를 위하여 연구에 사용되는 식품의 생산/포장 및 라벨링에서 언급된 내용 이외에, 각 군별로 고유코드의 할당 내역은 연구자가 봉인된 상태로 관리하며, 중대한 약물 이상반응 발생으로 부득이하게 해당 코드 열람이 필요한 경우*를 제외하고는 연구 종료 시까지 공개하지 않는다. 연구자는 선정된 연구대상자의 섭취 배정번호와 일치하는 고유코드 식품을 연구대상자에게 공급하며, 시험용/대조용 식품의 결손 및 파손 시에는 여분(고유코드별)을 사용함으로써 눈가림을 유지한다.

* 해당 코드 열람이 필요한 경우 눈가림해제절차: 연구대상자에게 중대한 약물이상반응이나 기타 중요한 임상적 상황으로 배정 된 군을 확인해야 하는 상황이라고 연구자의 판단이 들면 즉시, 전화나 Fax로 이 사실을 연구수탁기관의 모니터에게 알린다.
이 사실을 전달 받은 모니터는 의뢰사에 알리고 연구 관련자들과 눈가림 해제가 꼭 필요한 사항인지 논의를 통하여 눈가림 해제 여부를 결정 후 연구자에게 결과를 통지해 준다.
연구자는 눈가림 해제 수락을 통보 받은 경우에 한하여, 보관된 눈가림 봉투 중 해당 연구대상자의 배정번호와 일치하는 봉투를 개봉하여 시험용 식품인지 대조용 식품인지 확인 후, 필요하다면 그에 따른 처치를 실시하도록 한다. 눈가림해제 시에는 해당 연구대상자 정보와 해제 사유를 무작위 배정 코드 파기 기록지에 기록하도록 하며, 무작위배정 코드가 해제된 연구대상자는 연구를 지속 할 수 없다.

7.4. 무작위배정 방법

본 연구는 시험군 또는 대조군을 무작위 배정하여 평행시험으로 진행하며, 필요한 대상자 수는 탈락율(15%)을 고려하여 각 군당 65명씩 총 130명이다.
방문2(Baseline Visit, Week 0)에서 선정/제외기준에 적합한 모든 연구대상자에 대하여 블록화 무작위배정법의 할당코드에 의하여 각 군으로 배정한다.

무작위배정표는 SAS®system의 Randomization program으로 발생된 난수(A, B 의 Random number)의 순열을 연구대상자 번호 1번부터 순차적으로 적용시킨 것으로 SAS®를 통해 연구 전에 미리 고안하여 생성한다. 의뢰자는 시험 식품 포장 시 무작위 배정표에 따라 임상연구용 라벨을 부착한 후, 연구 시작 전에 연구기관에 공급한다.

연구기관에서는 동의서에 서명하고, 스크리닝 검사를 시행하는 연구대상자에게 다음의 스크리닝 번호를 부여한다.
AA-S ZZZ {AA: 기관코드, S: Screening의 첫글자, ZZZ: 일련번호(001, 002~)}

이후, 방문2에서 해당 연구대상자가 본 연구의 대상자로 적합한 경우에 다음의 연구대상자 번호를 순서대로 부여하고, 이때 일련번호와 같은 번호의 시험/대조 식품을 제공하여 섭취하게 한다.
AA-R ZZZ {AA: 기관코드, R: Randomization의 첫글자, ZZZ: 일련번호(001, 002~)}

연구기관 기관코드는 다음과 같다.
연세대학교 : YG

8. 예상 연구대상자 수와 산출 근거

8.1. 연구대상자 수

	시험군	대조군	총 대상자 수
최종 평가 예수(PP)	55	55	110
Drop-out(15%) 고려예수	65	65	130

대상자 선정, 제외기준에 적합한 130명 이상을 확보하여 섭취하도록 하고, Protocol에 명시된 PP기준에 적합한 최종 유효성 평가 예수로 110명 이상(군당 55명 이상)을 분석하기로 계획한다.

8.2. 산출근거

본 인체적용시험의 일차적인 목적은 투여기간 8주 후 NK cell activity의 증가가 시험군(바이오게르마늄 효모)이 대조군(placebo)과 비교하여 우월하다는 것을 증명하고자 한다.

・섭취 전(Baseline visit, week - 2), 섭취 후(Closing visit, week 8) 시험군과 대조군의 NK cell activity 평균값의 변화량 비교

적절한 연구대상자 수를 산정하기 위하여 본 연구의 주평가지표와 동일한 평가지표를 사용하여 연구를 한 후 그 결과를 보고한 참고문헌들 중 주평가변수가 통계학적으로 유의한 결과를 보인 참고문헌[45)]을 이용하여 연구대상자 수의 산정에 참고하였다. 참고문헌에서 보인 각 추정 값은 다음의 표와 같다.

평가지표	연구대상자 수(명)		평균(baseline- 복용 후)		표준편차(S.D)		S
	placebo	Test	placebo	Test	placebo	Test	
Cytotoxicity of NK cells	31	31	- 0.28	7.4	10.7	10.0	10.7

- 통계적검정력과 유의 수준은 각각 80%와 5% 임
- 시험약/위약의 복용 전,후 차이에 대한 표준편차 값은 주어져 있지 않아 복용 전, 후 값의 상관계수가 0.25라고 가정하고 추정하였음.
- 공통 표준편차 S는 두 집단의 표준편차 중 최대값으로 하였음.

본 연구의 유효한 연구대상자 수를 산정하기 위하여 다음을 가정한다.

1) 우위성시험(Superiority test)
2) 유의수준(Level of significance) 5%, 양측검정
3) 제 2종 오류(β)는 0.2로 하여 검정력(Power of the test)은 80%를 유지
4) 시험군과 대조군의 시험예수의 비율은 같다.
5) 기존의 연구 중 본 연구와 동일한 평가 변수인 NK cell activity를 이용한 연구인 논문의 결과를 이용하였고, 평가변수의 차이와 표준편차를 고려하여 표본의 수를 산정하였다. 평가변수의 차이는 기존연구의 효과의 임상적 유용 정도를 고려하여 약 75% 수준인 5.76을 사용하였다.

이를 검정하기 위한 연구의 가설은 다음과 같다.

H0 : $\mu t = \mu c$ (연구 종료 후 시험군과 대조군의 NK cell activity의 평균은 같다)
H1 : $\mu t \neq \mu c$ (연구 종료 후 시험군과 대조군의 NK cell activity의 평균은 다르다)

위의 1) – 5)를 가정하였을 때 연구에 필요한 시험예수는 다음과 같다. (양측검정)

$$n_t = \frac{(Z_{\alpha/2}+Z_{\beta})^2 x S^2 x 2}{d^2}$$

여기서,

$Z_{\alpha/2}$:표준정규분포에서 오른쪽 꼬리부분 면적이 $\alpha/2$ 가 되는 임계치 혹은 분위수

Z_{β}:표준정규분포에서 오른쪽 꼬리부분 면적이 β 가 되는 임계치 혹은 분위수

α : 제1종 오류의 크기

β : 제2종 오류의 크기

σ : 표준편차

d: 임상적으로 의미가 있는 시험군과 대조군간의 평균변화 차이

상기 식으로 연구대상자의 수를 계산하면, 군당 최소 대상자 수는 군당 약 55명 이다.

$$55 = \frac{(1.96 + 0.84)^2 x 10.7^2 x 2}{5.76^2}$$

따라서 본 연구의 유효성 평가 변수인 NK cell activity를 이용하여 계산된 55명을 군당 연구대상자 수로 정하고 탈락률(15%)를 고려하면 군당 등록할 연구대상자 수는 65명이며, 총 인원은 130명으로 한다.

9. 연구대상자 모집

본 연구는 만 25세 이상 만 75세 이하의 남, 여 중 스크리닝 검사에서 측정한 말초혈액 백혈구 수치가 $4X10^3$cells/ul 이상 ~ $8X10^3$cells/ul 미만인 사람으로 연구계획서와 연구자의 임상적 판단에 따라 연구대상자를 모집할 계획이다. 또한 지역 내 포스터를 부착하여 모집할 예정이며, 이를 통해 130명의 연구대상자가 등록될 때까지 지속한다.

10. 연구대상자 동의

연구책임자 및 연구책임자의 위임을 받은 연구자는 본 연구의 실시에 있어 연구대상자에게 연구내용 및 시험용 식품의 효과, 이상반응에 대해 사전에 충분히 설명하고 대상자의 동의를 얻는다.

스크리닝 결과 본 연구 참여가 가능한 사람을 대상으로 연구에 대한 정보를 제공하게 된다. 정보 제공 후 참여의사가 있는 대상자에게 본 연구담당자가 연구의 목적과 방법, 시험용 식품의 효과, 이상반응 및 안전성 등에 대한 모든 사항을 설명한 후, 연구 참여 여부를 숙고할 수 있도록 하고, 일정 시간 경과 후, 자발적인 의사로 동의서에 서명할 수 있도록 한다.

연구대상자의 서면 동의를 받는 항목은 대상자 동의서와 인체유래물 연구 동의서이다. 각 동의서에 서명을 받은 후 연구대상자 선정제외 기준을 만족하는지 선별하는 과정을 통해 선정기준을 만족하면 연구에 등록한다. 향후 연구 진행 시 인체유래물 연구 동의서에 동의한 인체유래물만을 연구에 사용한다. 연구대상자는 연구에 자발적으로 참여하고 본인의 의사에 따라 참여를 철회할 수 있다. 연구대상자가 연구에 참여하지 않거나 중도에 연구 참여 철회를 결정하더라도 연구대상자에 대한 어떠한 불이익이 발생하지 않으며 연구대상자가 원래 받을 수 있는 이익에 대한 어떠한 손실도 없다.

11. 연구방법

11.1. 연구의 설계

본 연구는 이중눈가림, 무작위배정, 위약대조 평행시험으로 디자인 되었다. 자의(또는 법정대리인)에 의해 연구 동의서에 서명 후 인구학적 조사, 병력/약물투여력 조사, 이학적검사, 신체계측, 체온측정, 활력징후, 임상병리검사(임신반응 검사 포함), WBC 수, 상기도 감염발생여부 등의 방문 평가를 실시하여 선정기준에 적합하고, 제외기준에 해당되지 않는 연구대상자에 한하여 본 연구에 등록되며 시험군 및 대조군 중 한 군으로 무작위 배정된다. 배정된 대상자는 총 8주간 시험용 식품 또는 대조용 식품을 섭취하게 된다. 각 군의 배정비율은 시험군 : 대조군 = 1 : 1로 한다.

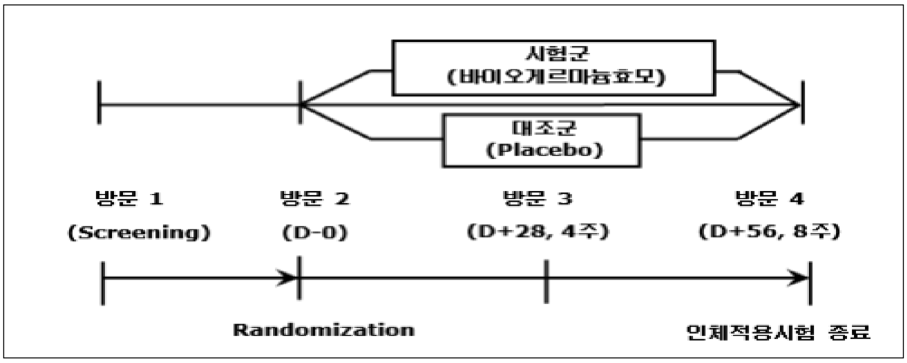

11.2. 시험 식품

11.2.1. 시험 식품의 개요

11.2.1.1. 시험식품 ***(***바이오게르마늄 효모***)***

- 주성분명: 바이오게르마늄 효모
- 성상 및 제형 : 경질캡슐

- 중량 : 300mg/캡슐
- 용법 및 용량 : 1일 2회, 1회 2캡슐(바이오게르마늄 효모로써 1.2g/day)
- 보관방법 : 실온 (직사광선을 피해 서늘한 곳에 보관)
- 원료 및 배합비율(300mg/캡슐 중)

원료명	배합비율(%)	함량(mg)
바이오게르마늄 효모	100.0	300.0
**합 계**	***100.0***	***300.0***

- 제조사 : (재)춘천바이오산업진흥원

11.2.1.2. 대조식품 ***(Placebo)***

- 주성분명: 옥수수전분
- 성상 및 제형 : 경질캡슐
- 중량 : 300mg/캡슐
- 용법 및 용량 : 1일 2회, 1회 2캡슐
- 보관방법 : 실온 (직사광선을 피해 서늘한 곳에 보관)
- 원료 및 배합비율 (300mg/캡슐 중)

원료명	배합비율(%)	함량(mg)
옥수수 전분	96.661	289.985
된장향(액상)	0.316	0.948
미숫가루향(액상)	0.105	0.315
식용색소 천연브라운	2.000	6.000
식용색소 노랑	0.386	1.157
식용색소 빨강	0.406	1.217
식용색소 초록	0.126	0.378
**합 계**	***100.0***	***300.0***

- 제조사 : (재)춘천바이오산업진흥원

11.2.2. 시험용 식품의 생산**/**포장 및 라벨링

시험용 식품(시험/대조 식품)은 의뢰자가 제조 후, 포장하여 연구자에게 공급한다.

시험 식품으로 생산되는 식품은 각 식품의 외형 및 성상이 동일하여 육안으로는 차이가 관찰되지 않아야 하며, 중량 차이도 크지 않아야 한다. 또한 시험용 식품과 대조용 식품은 동일한 라벨을 부착함으로써 연구대상자 및 연구자에 대하여 눈가림(Blind)이 유지되도록 한다.

시험 식품 라벨의 기재는 의약품 등 안전에 관한 규칙 제69조 제6항에 따르며 군별 차이가 노출되지 않도록 제조번호(Lot No.)는 시험용 식품, 대조 식품 제조번호를 함께 병기한다. 눈가림을 위한 샘플의 생산과 유지방법은 다음과 같다.

① 분리보관: 시험 식품과 대조 식품들은 라벨링 작업 전까지 제조번호별로 섞이지 않게 별도 보관한다.

② 라벨링: 아래의 내용이 포함된 라벨을 제작한다.

1. 무작위배정번호
2. 고유코드번호
3. "인체적용시험용"이라는 표시
4. 연구계획서 번호: GRT_Bio_Ge
5. 성분명/코드명: 바이오게르마늄 효모 또는 전분으로 된 위약
6. 제조번호: ____________ 또는 ______________
7. 연구계획 승인을 받은 자의 명칭, 주소 및 전화번호
게란티제약㈜, 서울 강남구 테헤란로 7길 22 과학기술회관 신관 408호,
02- 2226- 9088
8. 사용(유효)기간 또는 재검사일자
9. 저장방법: 실온(직사광선을 피해 서늘한 곳에 보관)
10. 섭취방법: 1일 2회, 1회 2캡슐
11. 어린이 손에 닿지 않는 곳에 보관
"연구 외의 목적으로 사용할 수 없음"이라는 표시

③ 포장 및 고유 코드의 기록: 시험 식품을 각각 4주분 씩 소분 포장한 후, 무작위배정표에 따라 고유코드를 포장 라벨지에 기록한다.

11.2.3. 시험 식품의 교부

시험 식품의 불출은 연구자가 배부한다. 이 때, 연구자의 서명이 있는 처방전에 의해 행해져야 하며 방문일, 시험 식품 처방 수 등이 기록되어야 한다.

11.2.4. 시험 식품의 관리 및 기록

연구담당자는 수불대장에 연구대상자의 무작위배정번호, 처방일, 시험 식품 불출 수량, 시험 식품 재고량 등을 기록하여야 한다. 시험 식품의 입고가 필요한 경우 연구 수탁기관의 모니터요원에게 이를 알려야 하며, 모니터요원은 의뢰자에게 이를 요청한다. 또한, 모니터요원은 정기적으로 관리•기록된 수불대장을 확인한다.

11.2.5. 시험 식품의 회수 및 폐기

연구담당자는 연구대상자로부터 시험 식품을 회수해야 하며, 모니터요원은 회수된 시험 식품 및 미사용 된 시험 식품을 의뢰자에게 반납한다.

11.2.6. 시험 식품 섭취량, 섭취방법 및 섭취기간

*11.2.6.1. 1*일 섭취량 및 섭취방법

- 시험군(바이오게르마늄 효모) : 1일 2회, 1회 2캡슐 (바이오게르마늄 효모로써 1.2g/day)
- 대조군(Placebo) : 시험군과 동일한 방법으로 섭취

11.2.6.2. 섭취기간

시험 식품 또는 대조 식품을 8주(56일)간 섭취

11.2.6.3. 섭취량 설정사유

1) 섭취량: 바이오게르마늄 효모 1.2g/day

2) 근거내용

50-75세의 남, 여 50명을 대상으로 바이오게르마늄 효모를 1일 1.2g을 2개월간 위약대조로 섭취시킨 결과, 혈액학적 검사 및 혈액 생화학적 검사에 유의적인 변화가 나타나지 않았으며, 시험물질 섭취 군에서 B세포와 TNF-α가 섭취 전에 비해 유의적으로 증가하였다. 또한 대조군과 바이오게르마늄 효모 섭취 군의 군간 비교에서 B세포, NK세포, TNF-α에서 유의적인 차이를 보였으므로 섭취량을 바이오게르마늄 효모로써 1.2g/day 으로 식약처 개별인정 인허가에 의거하여 설정하였다.

11.3. 병용 금지약물 및 식품

다음 약물 및 식품의 사용은 안전성, 내약성 또는 유효성의 평가를 방해할 수 있다. 따라서, 다음에 열거하는 약물은 연구기간 동안 병용 투여를 금지한다.

1) 면역관련 건강기능식품 및 식품(예: 홍삼, 인삼, 클로렐라, 알콕시글리세롤 함유 상어간유,알로에 겔, 한약 등)
2) 면역 관련된 예방접종(예: Influenza 등)

연구기간 동안 연구대상자가 이들 약물 및 식품 중 어느 것이라도 투여한 경우에는 모니터요원에게 즉시 알려야 한다. 모니터요원에게 연락될 때까지는 연구대상자를 연구에서 중도탈락 시켜서는 안 된다. 이들 약물을 투여한 연구대상자의 연구 중단 여부 결정은 사례 별로 연구자와 상의한 뒤에 연구결과(유효성)에 영향을 미칠 수 있는 경우 연구에서 제외/탈

락될 수 있다.

시험 식품 섭취를 시작한 후에 새로운 약물을 투여한 경우 연구자(또는 담당자)에게 보고하도록 연구대상자를 교육한다. 연구대상자의 시험 식품 섭취 이후의 모든 약물 투여 및 유의한 비약물 치료(물리치료 및 수혈 포함)를 증례기록서 '병용약물 및 병용요법 확인'에 기재한다.

12. 관찰 항목 및 검사 방법

12.1. 관찰항목

12.1.1. 연구대상자 동의 및 인구학적 조사

연구에 들어가기 전, 본 연구의 목적과 내용에 대하여 연구대상자(또는 법정 대리인)에게 상세히 설명하고, 서면으로 동의를 받으며, 서면 동의를 받는 순서에 따라 스크리닝 번호를 부여한 후 인구학적 정보를 조사한다. 인구학적 정보 조사의 기록사항은 서면 동의 여부 및 동의일자와 연구대상자의 이니셜, 성별, 생년월일, 연령, 면역질환 관련 증상 가족력, 흡연여부, 음주여부, 운동여부 등을 조사한다.

12.1.2. 병력***/***약물 투여력 조사***,*** 병용약물 및 병용요법

방문1과 방문2에서 문진과 과거 진료 기록 점검 등을 통하여 조사한다. 스크리닝 시점을 기준으로 3개월 이내 외과적 수술력을 포함한 병력을 상세히 조사하여 기록한다. 과거력 및 현 병력에 대하여 발생시기(발생년도 또는 발생년월), 치료기간, 지속여부, 연구자의 의견 등을 기재한다. 약물 투여력은 스크리닝 시점을 기준으로 4주 이내의 선행약물을 모두 확인한다.
시험 식품 섭취 이후 방문2와 방문3에서 병용약물 및 병용요법을 확인한다.

12.1.3. 이학적검사

방문1, 3, 4에 심혈관계, 폐 및 호흡기계, 위장관/간 및 담도계, 대사/내분비계, 신장/요로계, 생식기계, 근골격계, 피부 및 결합조직, 신경계, 정신계, 기타 신체기관에 대한 대상자의 임상적 상태를 근거로 이학적검사를 시행한다.

12.1.4. 활력징후***(***혈압***,*** 맥박***),*** 체온

연구대상자에게 10분 이상의 안정상태를 유지시킨 후 자동혈압계 EASY X 800(자원메디칼, Korea)을 이용하여 혈압, 맥박을 방문1, 3, 4에 측정한다. 각 방문에서 동일한 장비를 이용

하여 동일한 시간대에 동일한 연구 담당자가 동일한 팔을 측정할 수 있도록 한다. 담당자가 다른 경우 담당자를 동일하게 교육하여 편차를 최소화한다.
이상반응 점검을 위해 매 방문 시마다 비접촉체온계 써모파인더 FS-300(HuBDIC, Korea)를 이용하여 눈썹 위 이마로부터 2~3cm 떨어져 체온을 측정한다.

12.1.5. 임상병리검사

방문1, 4(또는 중도탈락 방문)에서 임상병리검사를 실시하여 연구대상자의 전신적인 건강상태를 평가한다. 채혈은 연세대학교 임상영양유전연구실로 방문하여 담당 연구병리사를 통해 이루어진다. 연구자는 연구대상자로 하여금 검사 전 12시간 금식상태로 내원하도록 지시하며, 연구대상자의 인체유래물 동의여부에 따라 수집된 검체는 본 연구 외 다른 학술 연구에 이용 될 수 있으며, 분석 후 남은 검체는 연세대학교 관련 규정 절차에 따라 폐기한다.

- 혈액학적 검사(7종) : WBC, RBC, Hemoglobin, Hematocrit, Platelet count, Lymphocyte, Monocyte
- 혈액화학적 검사(10종) : AST(GOT), ALT(GPT), Total protein, Glucose, Total cholesterol, BUN, Creatinine, Uric acid, Ca, P
- 소변검사(10종) : SG, pH, WBC(Leukocyte), Nitrite, Protein, Glucose, Ketone, Urobilinogen, Bilirubin, Erythrocyte
- 임신반응검사 : Urine HCG(방문1과 방문4에서 가임기 여성만 해당)

주목 할 만한 임상병리학적 비정상치 기준을 벗어나는 임상병리검사 결과는 대상자의 증례기록서(CRF) 연구자 의견(Comments)란에 평가를 기재하고, 연구자가 적절하다고 판단하는 경우 추가 평가를 실시하여야 한다. 임상병리학적 비정상치로 인하여 임상적 징후/증상이 야기되거나 치료 중재술이 필요한 경우 대상자의 증례기록서(CRF)의 ‘이상반응’ 페이지에 진단 또는 의학적 상태를 입력하여야 한다.

12.1.6. 식이조사

연구대상자로 적합하다고 판정될 경우, 연구기간 동안 대상자에게 평소 섭취하던 일반적인 식사형태, 식이섭취량을 유지하도록 권고한다.

방문2에는 연구대상자의 평상 시 식이조사를 위해 하루 전날 섭취한 식사를 24시간 회상법으로 연구담당자가 자세히 조사하여 작성한다.

연구 기간 동안의 대상자의 식이를 조사하기 위해 방문2, 3에 대상자에게 식이조사지를 배부한다. 대상자는 식이조사지에 다음 방문일 전 최근 일주일 중 3일 동안(가능하다면 주말 1일 포함) 평소의 식사와 유사한 날의 식사를 기록한다. 방문3, 4에 연구 담당자는 작성된

대상자의 식이조사지를 확인한다.

연구 기간 동안 작성된 식이조사지는 한국영양학회의 CAN Pro 3.0 (한국영양학회(사))을 이용하여 식품 및 영양소 섭취량을 분석한다.

12.1.7. SRI(Stress Response Inventory)

방문2 에 실시하며, SRI(Stress Response Inventory, 스트레스 반응 척도)는 정상인들을 대상으로 감정, 신체, 인지, 행동 영역의 반응들을 모두 포함하는 스트레스 반응 척도를 평가하기 위해 개발되었다[46]. 전체 39문항, 7개 하위척도로 구성되었다. 하위척도는 '긴장' 6개 문항, '공격성' 4개 문항, '신체화' 3개 문항, '분노' 6개 문항, '우울' 8개 문항, '피로' 5개 문항, '좌절' 7개 문항으로 각각 이루어졌다.
2번, 16번, 17번, 30번, 31번, 37번이 '긴장' 6개 문항이고, 18번, 28번, 29번, 33번이 '공격성' 4개 문항이고, 6번, 7번, 10번이 '신체화' 3개 문항이고, 4번, 24번, 25번, 26번, 27번, 34번이 '분노' 6개 문항이고, 5번, 15번, 19번, 22번, 23번, 35번, 38번, 39번이 '우울' 8개 문항이고, 1번, 12번, 13번, 14번, 36번이 '피로' 5개 문항이고, 3번, 8번, 9번, 11번, 20번, 21번, 32번이 '좌절' 7개 문항이다.
본 척도는 '전혀 그렇지 않다'(0점), '약간 그렇다'(1점), '웬만큼 그렇다'(2점), '상당히 그렇다'(3점), '아주 그렇다'(4점)으로 기록되며, 점수 범위는 0점에서 156점까지로 점수가 높을수록 스트레스가 높음을 의미한다.

본 척도는 각 하위척도 및 척도 전체 점수를 고경봉 등이 연구를 통해 신뢰도 및 타당도를 검증하였다.

본 인체적용시험에서는 방문2(Baseline Visit, week 0)에서 SRI 설문을 실시하여 대상자에게 평상시의 스트레스 자각 정도에 관하여 평가한다.

12.1.8. 신체계측

방문1에서 신장, 방문1, 3, 4에서 체중을 측정하여 기록한다. 각 방문에서 동일한 장비(GL-150(Tech 지테크인터내셔날, Korea), Body fat analyzer TBF-105(Tanita Co., Japan))를 이용하여 측정 한다. 가벼운 옷차림 상태에서 신장은 0.1 cm, 체중은 0.1 kg 단위까지 반올림하여 측정한다.

12.1.9. 백혈구 수 *(White Blood Cell* 수*)*

백혈구 수는 말초혈액 검사의 하나로 말초혈액에서 보는 과립구계(호중구, 호산구, 호염기구), 림프계 및 단핵구계 세포의 혈액 단위 용적당 총수를 말한다. 생리적인 변동이 생기기 쉬워 운동 등의 스트레스, 저녁, 임신, 오심, 구토, 동통, 불안 시 증가한다. 정맥혈 채혈의

경우 채혈 후 6시간 이내에 측정해야 정확도가 높아지므로 가급적이면 일정한 시각(오전)에 앉은 상태에서 6시간 이내에 백혈구 검사를 시행하도록 추천한다. 말초혈중 백혈구 수의 증감을 보이는 질환 및 상태는 다음과 같다.

• 증가 (>10,000/ul) : 세균감염, 조직파괴, 폐암, 위암, 자궁암, 급성 및 만성 백혈병, 악성림프종, 비장 적출 후, 급성 출혈 후, 만성특발성 호중구증가증, 신부전, 간부전, 갑상선질환, 임신중독증, 자간, 당뇨병성 ketoacidosis, 알러지, 스트레스, 운동, 임신, 분만, 식후 등
• 감소 (<4,000/ul) : 속립결핵, 중증감염증, typhus fever, malaria, AIDS, 골수염, 암의 골수전이, 비정형백혈병, 악성림프종, 심한 철결핍성 빈혈, 수혈 후, 자가면역성호중구감소증, 교원병, 점액수종, 고 glycine 혈증, 방사선, 진통제, 항암제, 항경련약, 항갑상선약, 항 histamine약, 항생물질, 간경변, 악액질, 심한 영양불량 등

본 연구에서는 말초혈액 중 백혈구 수치가 4000cells/u 이상~8000cells/ul 미만인 성인을 연구대상자의 선정기준으로 하였다.

12.1.10. NK cell activity (Natural Killer cell 활성, 자연살해세포 활성)

NK cell(자연살해세포)는 림프구 중의 하나로 면역력을 나타내는 지표이다. 종양세포 또는 virus 감 염세포를 항체의 사용 없이도 선택적으로 파괴하는 작용이 있으며, 또한 자연발생 종양이나 발암물질에 의해서 발생되는 종양에 대하여 면역학적 감시기능의 역할을 담당하는 세포로 중요시 되고 있으며, NK cell 활성화 작용이 면역력을 증강시킨다고 보고되었다.
NK cell은 large granular lymphocytes(LGLs)로서 특정의 암세포나 바이러스에 감염된 세포 등을 파괴하며 종양의 형성이나 바이러스 감염에 대해 숙주방어에 관여할 뿐만 아니라 체액성 면역이나 조혈작용에도 매우 중요한 역할을 하고 있다.
특히, NK cell은 림프구의 극히 일부분이지만 면역 방어기전에 중요한 부분을 차지하는데 T림프구와 B림프구와는 달리 특별히 항원에 의한 면역이 만들어져 그에 대한 기억이 되어 있지 않아도 어떤 암세포나 바이러스에 감염된 세포들을 자발적으로 공격하는 세포독성을 나타내 표적세포를 살해함으로써 감염이나 이형성세포의 증식으로부터 우리 몸을 보호한다. 또한 항원과의 반응을 방해하여 일차적인 면역반응을 조절할 뿐 아니라 B림프구의 면역 글로블린 생산을 조절하는 면역계의 억제 세포(suppressor cells)이다.
NK cell 활성이 약해지거나 없어지는 것은 질환을 나타내는 신호라 볼 수 있어 이는 치료 중의 호흡기 감염 등의 합병증을 방지하기 위한 면역기능을 판단하는데 예측인자가 되기도 한다.

NK cell 세포 활성도는 세포독성 활성법을 이용하여 분석한다. 공복 시 채혈한 정맥혈을 동량의 RPMI medium 1640로 희석하고 Histopaque를 이용하여 PBMC(effector cell)를 분리한 후, NK cell에 대한 감수성이 높은 K562(Target cell)와 함께 여러 비율(2.5:1, 5:1,

10:1, 50:1)로 분주한 후 세포배양용 CO2 incubator에서 4시간 이상 배양한다. 배양액을 원심분리한 후 얻은 상층액을 CytoTox 96® NON-Radioactive Cytotoxicity Assay Kit(Promega Co., WI, USA)를 사용하여 효소-기질 반응을 시킨다. Victor x5 2030 multilabel plate reader(PerkinElmer, Hopkinton, USA)로 490nm 파장에서 흡광도를 측정한 후, 다음 공식을 통해 세포 독성을 도출한다.

Cytotoxicity(%)=(Experimental – Effector Spontaneous – Target Spontaneous)
/(Target maximum - Target spontaneous)X100

12.1.11. IFN-ɣ(Interferon-ɣ)

T 세포에 작용하여 Th1 세포의 분화를 촉진시킴과 동시에 Th2 세포의 증식을 억제한다. IFN-ɣ는 대식세포를 활성화하여 대식세포의 미생물 살해능력을 증가시키며, Th1 세포의 분화를 촉진하는 IL-12를 만들어내기도 한다. 또한 B세포를 활성화시켜 IgE 항체를 생성시키게 하는 IL-4의 생성을 조절한다. IL-4와 IFN-ɣ에 의한 면역 체계의 불균형이 여러 질환을 일으키는 원인이 되기도 한다.

IFN-ɣ 농도 측정을 위해 IFN gamma High Sensitivity Human ELISA Kit(Abcam plc, Cambridge Science Park, UK)를 이용하여 plate에 부착된 monoclonal antibody와 결합시킨다. Antibody와 결합된 IFN-ɣ에 biotinylated antibody와 streptavidin-HRP를 넣고 인큐베이터에 방치시킨 후, chromagen TMB substrate를 넣어 발색시키고 450nm 흡광도에서 Victor x5 2030 multilabel plate reader(PerkinElmer, Hopkinton, USA)를 이용하여 그 값을 측정한다.

12.1.12. IL-2, 6, 12(Interleukin-6, 12)

Cytokine은 당단백, 극미량에서 작용을 발휘한다. 그 작용을 발휘하기 위해서는 세포표면에 있는 특이수용체와 결합하여 작용하며 다중성과 상승성이 있다. 하나의 cytokine이 생산되면 다음의 cytokine이 생산되는 cytokine network가 존재한다. 생체의 항상성 유지에 불가결하지만 염증 같은 질환에서는 과잉으로 생산되어 병태를 형성한다는 특징이 있다. Cytokine은 생체에서 단독으로 작용하는 것이 아니라 항상 다른 cytokine과 협조하여 작용한다. 또한 그 활성을 억제하는 물질도 체내 (특히 혈중)에서는 대량으로 존재하여 항상성을 유지한다.

▪ IL-2

T림프구에서 생산되어 T림프구 뿐만 아니라 NK세포나 Killer T 세포 활성화, B세포 증식이나 분화에 관여한다. 즉, 생체방어와 조혈에 중요한 역할을 수행한다. 각종 cytokine을 생산하는 helper T 세포 증식을 촉진하는 것으로 IL-2 측정을 통해 helper T 세포기능 뿐만 아

니라 면역응답능을 추측할 수 있다. SLE(Systemic lupus erythematosus), Rheumatoid arthritis, Hodgkin's병, 제 1형 당뇨, Condyloma acuminatum 에서 IL-2 생산능이 저하된다.

IL-2의 농도 측정을 위해 Human Interleukin-2(IL-2) ELISA Kit(Cusabio Biotech, USA)를 이용하여 plate에 부착된 antibody와 결합시킨다. Antibody와 결합된 IL-2에 ABC solution을 넣고 인큐베이터에 방치시킨 후, TMB agent를 넣어 발색시키고 450nm 흡광도에서 Victor X5 2030 multilabel plate reader(PerkinEler, Hopkinton, MA)를 이용하여 그 값을 측정한다.

■ IL-6

B세포 분화인자(BCDF)로 cloning 되는데, 실제로는 면역계, 신경계, 내분비계, 조혈계, 간 등에 작용하여 생체의 항상성 유지에 깊이 관여하고 있다. 또 한편으로 염증시에 과잉으로 생산되므로 그 병태 형성에 관계하는 것이 추측되고 있다. IL-6 transgenic mouse 에서는 고 ɣ-globulin 혈증, 형질세포종, mesangium 증식성 신염 등이 나타난다. 또한 IL-6 의 거핵구 혈소판 조혈에 대한 작용이 주목되고 있어 혈소판 감소증에의 임상 응용이 기대되고 있다.

IL-6 농도는 Bio-Plex™Reagent Kit(Bio-Rad Laboratories, Hercules, USA)를 이용하여 분석하고자 하는 cytokine에 맞는 antibody를 사용하여 측정한다. Plate에 2종의 antibody를 부착시킨 후, 혈액 샘플을 각각의 well에 주입시켜 antibody와 혈액 속의 cytokine이 결합할 수 있도록 1시간의 반응 시간을 준다. Antibody-cytokine complex에 stereptavidin를 부착시켜 발색시키고 Bio-plex(Bio-Rad Laboratories, Hercules, USA)를 사용하여 그 값을 측정한다.

■ IL-12

활성화 B세포나 단핵구, macrophage계 세포 등에서 생산된다. 사람 T세포의 IFN-ɣ유도활성이 있어서 NK활성도 증강하기 때문에 natural killer stimulatory factor (NKSF)로도 알려져 암치료의 응용이 기대되고 있다.

IL-12의 농도 측정을 위해 High Sensitivity Human IL-12(p70) ELISA kit(Genway Biotech Inc., San Diego, CA)를 이용하여 plate에 부착된 antibody와 결합시킨다. Antibody와 결합된 IL-12에 ABC solution을 넣고 인큐베이터에 방치시킨 후, TMB agent를 넣어 발색시키고 450nm 흡광도에서 Victor X5 2030 multilabel plate reader(PerkinEler, Hopkinton, MA)를 이용하여 그 값을 측정한다.

12.1.13. TNF-α(Tumor Necrosis Factor-α, 종양괴사인자*-α)*

Th1 세포로부터 생성되는 세포 활성물질로서 단핵구 및 다른 형태의 세포를 자극하여 백혈구의 보충에 한 역할을 담당하는 케모카인(chemokine)을 분비토록 하며 또한 염증성 백혈구를 활성화시켜 미생물을 죽이도록 한다. TNF-α는 호중구 활성화기능이 강하여 호산구 및 단핵식균세포에도 영향을 미친다. TNF-α가 저농도로 계속 생성되는 경우, 조직의 재배열(tissue remodeling)이 일어나며, 새로운 혈관의 형성을 유도하는 혈관생성인자(angiogenesis factor) 및 결합조직의 침착을 야기하는 섬유아세포 성장인자로서도 작용한다.

TNF-α 농도는 Bio-Plex™Reagent Kit(Bio-Rad Laboratories, Hercules, USA)를 이용하여 분석하고자 하는 cytokine에 맞는 antibody를 사용하여 측정한다. Plate에 antibody를 부착시킨 후, 혈액 샘플을 각각의 well에 주입시켜 antibody와 혈액 속의 cytokine이 결합할 수 있도록 1시간의 반응 시간을 준다. Antibody-cytokine complex에 stereptavidin를 부착시켜 발색시키고 Bio-plex(Bio-Rad Laboratories, Hercules, USA)를 사용하여 그 값을 측정한다.

12.1.14. IgG1, IgG2(Immunoglobulin G), IgM

IgG는 면역 반응을 주로 담당하는 항체이며 2차 면역 반응 과정 동안 대량 생산되고, 보체 연쇄반응을 활성화시킨다. 미생물에 옵소닌으로 작용하여 대식세포와 중성구의 식균작용을 도우며, 태반을 통과하고 모유에 분비되어 태아와 신생아의 면역 반응을 돕는다. 악성 림프종, 신부전, 당뇨병 및 미숙한 신생아에서 IgG의 감소가 나타난다. IgM은 응집작용, 세포용해 방응 및 식균작용을 통해 세균을 박멸하는 역할을 하며, IgG의 생성을 촉진시키는 동시에 항체의 효과를 높이는데 필요한 보체를 생성한다.

serum 0.5ml가 버퍼와 항체 시약과 혼합되면, 검체의 IgG는 시약내의 Antihuman IgG Antibody에 특이적으로 결합하여 용액의 혼탁도를 증가시키는 불용성의 응집물을 생성한다. 이 용액의 혼탁 정도를 광학적으로 측정하고 이것은 검체 중의 IgG의 활성과 비례하게 된다. IgG1, IgG2, IgM은 면역 비탁법을 이용한 원리로, 시약내 특이항체와 침전물을 형성하며 340nm 파장에서 혼탁도를 측정한다.

12.1.15. 자신에 의한 개선도 평가

방문3, 4에 섭취 전(방문1)과 비교하여 연구대상자 자신이 다음과 같은 5단계로 면역력 증진 개선도 평가를 실시한다.

점수	구 분	기 준
1	매우 우수	전반적으로 증상의 현저한 개선

2	우 수	전반적으로 증상의 개선
3	불 변	섭취 전과 차이 없음
4	악 화	전반적으로 증상의 악화
5	매우악화	전반적으로 증상의 현저한 악화

_12.1.16._ 면역 등과 관련한 유전자 검사

TNF-a는 주로 활성화된 대식세포 및 NK세포에서 분비되며, TNF-a G308A 유전자는 면역조절 유전자 중 하나로 다양한 염증 및 면역질환과 상관성을 가진다. 본 연구에서는 TNF-a G308A 유전자 다형성 분석을 통해 NK세포 활성도와의 상관관계를 연구함으로써 좀 더 객관적이고 과학적인 면역 조절 효능 연구를 진행하고자 샘플을 보관한다. TNF-α(SNP name : G-308A rs1800629)의 유전자다형성 분석은 single base extraction을 이용하여 SNaPshot Assay, TaqMan assay, SNP-IT assay 등의 방법으로 측정한다. ㈜ DNA Link에는 대상자의 개인식별정보가 제공되지 않고 고유번호가 부여된 자료가 제공된다.

_12.1.17._ 인체유래물 등 수집방법

인체유래물 연구동의서에 동의를 받은 후 세브란스병원 내분비내과 임상교수(신동엽)의 감독하에 연세대학교 임상영양유전연구실 소속 임상병리사(백유미)가 채혈을 진행한다. 혈액은 4회(총 75mL), 소변은 2회(총 10mL) 수집된다. 채혈과 소변검사는 연구대상자의 12시간 공복 후에 이뤄진다.

[방문1]에는 혈액 총 11mL가 혈액학적 및 혈액화학적검사(9mL)와 2차 연구(2mL)를 위해 수집되고, 소변 총 5mL가 소변검사 및 임신반응검사(3mL)와 2차 연구(2mL)를 위해 수집된다. 선정기준에 적합한 연구대상자는 [방문2]에서 NK cell activity(9mL)와 면역 항체검사 및 관련지표검사(7mL), 면역관련 유전자검사(2mL)를 위해 총 18mL가 수집된다. [방문3]에서는 말초혈액 백혈구 수치 검사(3mL), NK cell activity(9mL)와 면역 항체검사 및 관련지표검사(7mL)를 위해 총 19mL가 수집되고, [방문4] 에서는 본 연구를 위하여 혈액학적 및 혈액화학적검사(9mL), NK cell activity(9mL), 면역 항체검사 및 관련지표검사(7mL)와 2차 연구(2mL)를 위해 혈액 총 27mL와 소변 총 5mL를 소변검사 및 임신반응검사(3mL)와 2차 연구(2mL)를 위해 수집이 이뤄진다. 단, 연구대상자가 2차 연구를 위한 제공여부에 동의하지 않을 경우 연구대상자의 추가분석을 위한 샘플은 수집되지 않는다.

본 연구에 사용 후 남은 검체는 인체유래물 연구 동의서에 동의를 한 경우에만 참여자의 동의내용(연구목적, 종류와 수량, 보존기간, 2차 사용 승낙여부, 개인식별정보)에 따라서 관리된 후에 사용됩니다. 인체유래물 연구에 동의하지 않는 경우에는 본 연구를 위하여 사용된 후에, 남은 검체는 연세대학교 관련 규정 절차에 따라 폐기한다.
각 방문 시 수집되는 인체유래물의 종류, 수집량, 검사항목 및 기관에 대한 자세한 사항은 아래와 같다.

방문***1***

<table>
<tr><th>종류</th><th colspan="2">검사항목</th><th>수집량</th><th>검사기관</th></tr>
<tr><td rowspan="4">혈액
(11mL)</td><td colspan="2">혈액학적검사
WBC, RBC, Hemoglobin, Hematocrit, Platelet count, Lymphocyte, Monocyte</td><td>3mL</td><td>연세대학교
임상영양유전연구실</td></tr>
<tr><td rowspan="2">혈액화학적검사</td><td>AST(GOT), ALT(GPT), Glucose,
Total cholesterol,
BUN, Creatinine,
Uric acid</td><td>4.5mL</td><td>(재)서울의과학연구소
(SCL)</td></tr>
<tr><td>Total protein, Ca, P</td><td>1.5mL</td><td>(재)서울의과학연구소
(SCL)</td></tr>
<tr><td colspan="2">2차 연구를 위한 검체</td><td>2mL</td><td>연세대학교
임상영양유전연구실</td></tr>
<tr><td rowspan="3">소변
(5mL)</td><td colspan="2">소변검사</td><td>2mL</td><td>연세대학교
임상영양유전연구실</td></tr>
<tr><td colspan="2">임신반응검사</td><td>1mL</td><td>연세대학교
임상영양유전연구실</td></tr>
<tr><td colspan="2">2차 연구를 위한 검체</td><td>2mL</td><td>연세대학교
임상영양유전연구실</td></tr>
</table>

방문***2***

<table>
<tr><th>종류</th><th>검사항목</th><th>수집량</th><th>검사기관</th></tr>
<tr><td rowspan="4">혈액
(18mL)</td><td>면역 관련 유전자검사</td><td>2mL</td><td>(주)DNA link</td></tr>
<tr><td>NK cell activity</td><td>9mL</td><td>연세대학교
임상영양유전연구실</td></tr>
<tr><td>면역관련지표검사
IFN-γ, IL-2, IL-6, IL-12, TNF-α</td><td>5mL</td><td>연세대학교
임상영양유전연구실</td></tr>
<tr><td>면역항체검사
IgG1, IgG2, IgM</td><td>2mL</td><td>(재)서울의과학연구소
(SCL)</td></tr>
</table>

방문***3***

종류	검사항목	수집량	검사기관
혈액<br>(19mL)	백혈구 수	3mL	연세대학교<br>임상영양유전연구실
	NK cell activity	9mL	연세대학교<br>임상영양유전연구실
	면역관련지표검사<br>IFN-γ, IL-2, IL-6, IL-12, TNF-α	5mL	연세대학교<br>임상영양유전연구실
	면역항체검사<br>IgG1, IgG2, IgM	2mL	(재)서울의과학연구소<br>(SCL)

방문*4*

종류	검사항목		수집량	검사기관
혈액<br>(27mL)	혈액학적검사<br>WBC, RBC, Hemoglobin, Hematocrit, Platelet count, Lymphocyte, Monocyte		3mL	연세대학교<br>임상영양유전연구실
	혈액화학적검사	AST(GOT), ALT(GPT), Glucose, Total cholesterol, BUN, Creatinine, Uric acid	4.5mL	(재)서울의과학연구소<br>(SCL)
		Total protein, Ca, P	1.5mL	(재)서울의과학연구소<br>(SCL)
	NK cell activity		9mL	연세대학교<br>임상영양유전연구실
	면역관련지표검사<br>IFN-γ, IL-2, IL-6, IL-12, TNF-α		5mL	연세대학교<br>임상영양유전연구실
	면역항체검사<br>IgG1, IgG2, IgM		2mL	(재)서울의과학연구소<br>(SCL)
	2차 연구를 위한 검체		2mL	연세대학교<br>임상영양유전연구실
소변<br>(5mL)	소변검사		2mL	연세대학교<br>임상영양유전연구실
	임신반응검사		1mL	연세대학교<br>임상영양유전연구실
	2차 연구를 위한 검체		2mL	연세대학교<br>임상영양유전연구실

12.1.17.1. 개인식별정보 및 임상정보

개인식별정보로 이니셜, 생년월일, 나이 등을 수집한다. 연구대상자 관련 임상정보로는 기타 질환 과거력 및 수술력, 가족력 그리고 현재 복용중인 치료제 여부를 조사한다.

12.1.18. 선정기준/제외기준 확인*(*연구대상자 적합성 평가*)*

방문1에 이루어진 대상(또는 법정 대리인) 동의여부와 인구학적 조사, 병력조사, 약물투여력 조사, 이학적검사, 신체계측, 체온측정, 활력징후, 임상병리검사, WBC 수 등의 조사 결과를 종합하여 8.1 항 및 8.2 항의 선정기준/제외기준에 적합한 대상자인지를 방문1, 2에 평가하

여 기록한다.

12.1.19. 무작위배정 및 섭취

방문1, 2에서 선정기준 및 제외기준에 적합하다고 평가가 이루어진 대상자를 대상으로 시험군 또는 대조군 중 한 군으로 무작위배정하고, 무작위배정번호에 따라 시험 식품을 섭취하게 한다. 섭취방법은 시험 식품을 받은 날부터 섭취하도록 하며, 다음 방문일 전날까지 섭취하도록 지도한다.

12.1.20. 이상반응 점검

이상반응에 대한 정보는 무작위배정 방문 이후부터 대상자에게 우회적으로 질문(Non- directive questioning)하여 탐색해야 한다. 또한 방문 시 또는 방문 기간 사이에 대상자가 자발적으로 보고하거나 이학적 검사, 임상병리검사 또는 기타 평가를 통하여 확인될 수 있다. 이상반응 조사에는 발현일 및 소실일, 이상반응의 정도 및 결과, 시험 식품과 관련하여 취해진 조치 및 시험 식품과의 인과관계, 시험 식품 이외 의심되는 약제명, 이상반응에 대한 치료 여부 및 내용 등이 포함된다.

이상반응으로 분류되는 대표적인 증상조사에 대하여 아래에 기술하였다.

(1) 발열에 대한 점검

발열은 체온을 기록하고 발현 정도를 이상반응 평가기준에 따라 기록하며, 약제투여 및 시험 식품과의 인과관계를 평가 한다.

(2) 자타각적 증상에 대한 점검

자타각적 증상은 연구자의 진료를 통하여 이상반응 발현 여부를 조사하고, 평가 기준에 따라 발현 정도를 기록하며, 약제 투여 및 시험 식품과의 인과관계도 평가한다.

(3) 임상병리검사에 대한 점검

임상병리검사에서 어떤 비정상적인 변화는 발현 증상에 대한 예상원인과 임상병리검사 시검사내용에 지장을 줄 수 있는 요인(심각한 피로 등)이 있었는지 면담을 통하여 기록한다. 약제투여 및 시험 식품과의 인과관계도 평가 한다.

12.1.21. 순응도 확인

시험용/대조용 식품의 섭취상황에 대하여 방문2, 3에 시험용/대조용 식품 처방 후 연구담당자가 기록하고, 방문3, 4 연구대상자가 지참하고 온 시험용/대조용 식품의 잔량을 확인하여 연구담당자가 점검한다. ‘시험용/대조용 식품 처방 당일 1일치의 양 섭취~이번 방문일 하루 전날’ 섭취함을 원칙으로 하며, 남은 시험 식품은 연구담당자에게 반납하도록 한다. 순응도

확인 시 섭취율(%)은 반올림하여 소수점 1자리까지 표기한다.

• 섭취 순응도(%) = 실제 섭취한 식품 수 / 섭취해야 하는 식품 수 x 100

12.2. 관찰 검사 방법 (방문별)

12.2***.1.1***차 방문***(Screening visit, week - 2*** 이내***)***

본 연구에 참가하는 대상자는 연구에 대한 설명을 듣고, 다음 평가를 받는다.

① 연구 참여 전에 연구 과정을 설명하고, 연구대상자에게 서면 동의서를 받는다.
② 연구대상자는 순서대로 스크리닝 번호를 지정 받는다.
③ 연구대상자의 인구학적 정보, 병력(외과적 수술력을 포함한 과거력 및 현병력) 및 약물 투여력을 조사한다.
④ 활력징후(혈압, 맥박) 및 체온, 신체계측(신장, 체중)을 측정한다.
⑤ 이학적검사를 실시한다.
⑥ 임상병리 검사를 실시한다.
⑦ 가임기 여성 연구대상자의 경우 임신반응 검사를 실시한다.
⑧ 유효성 평가(WBC 수)를 실시한다.
⑨ 상기도 감염 발생여부를 조사한다.
⑩ 선정/제외 기준에 대해 1차적으로 적합성을 판정한다.
⑪ 연구대상자의 다음 방문일을 지정한다.

12.2***.2. 2***차 방문***(Randomization visit, week 0)***

이 방문은 최초 방문일 이후 14일 이내에 이루어지고, 다음 평가를 받는다.

① 지난 방문과 비교하여 병력/약물 투여력의 변화여부를 확인한다.
② 체온을 측정한다.
③ 이제까지의 모든 검사 및 평가결과를 종합하여 선정기준/제외기준의 적합성을 평가한다.
④ 유효성 평가(NK cell activity, IFN-γ, IL-2, 6, 12, TNF-α, IgG1, IgG2, IgM)를 실시한다.
⑤ 면역 관련 유전자 검사를 실시한다.
⑥ SRI설문을 실시한다.
⑦ 무작위배정하여 배정번호를 부여한다.
⑧ 식이조사를 실시하고, 다음 방문 시 제출할 식사일지를 배부한다.
⑨ 1차 시험/대조 식품을 처방하고, 섭취 방법에 대하여 교육한다.

⑩ 대상자의 다음 방문일을 지정한다.

12.2.***3. 3***차 방문***(Interim visit, Week 4)***

이 방문은 방문2 이후 28일(±7일)에 이루어지고, 다음 평가를 받는다.

① 이상반응 유무를 확인한다.
② 지난 방문과 비교하여 병용약물 및 병용요법을 확인한다.
③ 활력징후(혈압, 맥박) 및 체온, 체중을 측정한다.
④ 이학적검사를 실시한다.
⑤ 유효성평가(WBC 수, NK cell activity, IFN-ɣ, IL-2, 6, 12, TNF-α, IgG1, IgG2, IgM, 연구대상자 자신에 의한 개선도 평가)를 실시한다.
⑥ 식이조사를 실시하고, 다음 방문 시 제출할 식사일지를 배부한다.
⑦ 1차 시험/대조 식품 반납을 확인하고 섭취 및 순응도를 확인한다.
⑧ 2차 시험/대조 식품을 처방하고, 섭취방법을 다시 한 번 설명한다.
⑨ 연구대상자의 다음 방문일을 지정한다.

12.2.***4. 4***차 방문***(Closing visit, Week 8)***

이 방문은 방문2 이후 56일(±5일)에 이루어지고, 다음 평가를 받는다.

① 이상반응 유무를 확인한다.
② 지난 방문과 비교하여 병용약물 및 병용요법을 확인한다.
③ 활력징후(혈압, 맥박) 및 체온, 체중을 측정한다.
④ 이학적검사를 실시한다.
⑤ 임상병리 검사를 실시한다.
⑥ 가임기 여성 연구대상자의 경우 임신반응 검사를 실시한다.
⑦ 유효성평가(WBC 수, NK cell activity, IFN-ɣ, IL-2, 6, 12, TNF-α, IgG1, IgG2, IgM, 연구대상자 자신에 의한 개선도 평가)를 실시한다.
⑧ 식이조사를 실시한다.
⑨ 2차 시험/대조용 식품 반납을 확인하고 섭취 및 순응도를 확인한다.

12.2.***5.*** 추가방문 ***(***필요시***)***

인체적용시험 진행 중 필요한 경우, 예를 들어 이상반응의 발생 여부가 의심되거나 이상반응 발생 후 추적조사가 필요한 경우, 중도탈락 등 연구대상자는 인체적용시험계획서에서 계획된 방문과는 별도로 추가 방문을 할 수 있다. 연구자는 대상자에게 정규방문 시 별도 추가 방문의 필요성을 주지시키고, 이상반응 발생 시, 연구자에게 즉각적으로 연락할 수 있도록 설명한다. 추가 방문에 대한 자료는 증례기록서와 근거문서 모두에 기록하여야 하며, 추

가 방문으로 인하여 시험계획의 일정이 변경되어서는 안 된다.

13. 효과 평가 기준 및 방법

13.1. 유효성 평가변수

1) NK cell activity (2.5:1, 5:1, 10:1, 50:1)
2) WBC 수
3) IFN-ɣ
4) IL-2, 6, 12
5) TNF-α
6) IgG1, IgG2, IgM
7) 연구대상자 자신에 의한 개선도 평가

13.2. 유효성 평가 방법

섭취 전(방문1, 2: week -2, 0)과 섭취 후(방문3, 4 : week 4, 8) 평가한 NK cell activity, 백혈구(WBC) 수, IFN-ɣ, IL-2, 6, 12, TNF-α, IgG1, IgG2, IgM, 연구대상자 자신에 의한 개선도 평가의 평균을 비교하고, 시험군과 대조군의 개선 정도를 분석, 비교하여 통계적으로 유의한 차이가 있는지 평가한다.

14. 안전성 평가 기준 및 평가 방법

연구책임자는 안전점검의 책임자로서 연구대상자의 안전을 위해 본 연구계획서에 명시된 이상반응 및 주의사항에 대해 사전에 숙지하고, 방문 시 또는 방문기간 사이에 연구대상자가 자발적으로 보고하거나, 이학적 검사, 임상병리검사 등을 통해 이상반응을 확인한다.
연구책임자는 연구기간 동안 관찰되거나 보고된 모든 이상반응을 보고할 책임이 있으며, 자세한 안전성 평가방법 및 보고방법은 아래 항목을 참조하여 시행한다.

14.1. 안전성 평가변수

1) 이상반응
2) 활력징후 (혈압, 맥박, 체온), 체중
3) 임상병리검사(혈액학적/혈액화학적 검사, 요검사)

14.2. 안전성 평가방법

이상반응, 활력징후(혈압, 맥박, 체온), 체중, 임상병리검사를 실시하여 섭취 전후 통계적으

로 유의한 차이가 있는지 평가한다. 또한, 섭취 군간 통계적으로 유의한 차이가 있는지 분석한다.

14.3. 이상반응을 포함한 안전성 평가방법, 평가기준 및 보고방법

14.3.1. 안전성 관련 용어의 정의

(1) 이상반응 (Adverse Event, AE)

시험 식품을 투여한 대상자에게 발생한 모든 유해하고 의도하지 않은 증후(症候, Sign, 실험실 실험 결과의 이상 등을 포함한다), 증상(症狀, Symptom) 또는 질병을 말하며, 해당 시험 식품과 반드시 인과관계를 가져야 하는 것은 아니다.

(2) 약물이상반응 (Adverse Drug Reaction, ADR)

시험 식품의 임의 용량에서 발생한 모든 유해하고 의도하지 않은 반응으로서 시험 식품과의 인과관계를 부정할 수 없는 경우를 말한다.

(3) 중대한 이상반응 • 약물이상반응 (Serious AE • ADR)

시험 식품의 임의 용량에서 발생한 이상반응 또는 약물이상반응 중에서 다음 어느 하나에 해당하는 경우를 말한다.

- 사망
- 생명을 위협
- 입원 또는 입원기간의 연장
- 지속적 또는 의미 있는 불구나 기능저하 초래
- 선천성 기형 또는 이상 초래
- 기타 의학적으로 중요한 상황

(4) 예상하지 못한 약물이상반응 (Unexpected Adverse Drug Reaction)

임상시험자자료집 또는 의약품의 첨부문서 등 이용 가능한 의약품 관련 정보에 비추어 약물이상반응의 양상이나 위해의 정도에서 차이가 나는 것을 말한다.

위에서 열거한 상황이 아니더라도 의학적으로 연구대상자의 안위와 건강상태에 중대한 영향을 미칠 것으로 사료되는 상황이 발생한 경우, 담당의사 및 관련 전문가의 의학적 판단에 따라 중대한 이상반응으로 간주할 것인가의 여부를 결정하고 이에 따라 적절한 조치를 취한다.

14.3.2. 평가 방법

- 본 연구에서 이상반응 평가는 연구 시작 전에 관찰되지 않은 증상이 섭취기간 중에 새로

이 나타난 증상으로서 시험 식품과의 인과관계에 상관없이 의도하지 않았던 증후(임상병리검사상 비정상치 포함) 및 증상과 시험 식품 사용과 관련된 일시적인 현상 등을 총칭한다.

- 시험 식품의 이상반응으로 예상되는 현상(증세 및 증상, 시작일, 지속기간 등)은 이상반응 조사서에 빠짐없이 기록되어야 하며, 기록되지 않은 것은 주관적인 증상으로 분류된다.
- 이상반응 정도에 대한 평가는 연구자가 평가기준을 참고하여 증상의 경중에 따라 단계별로 평가하는 것을 원칙으로 한다.

14.3.3. 평가 기준

14.3.3.1. 발열

발열은 체온을 측정하며, 다음과 같은 평가기준에 따라 단계별로 평가한다.

등급기준	Grade 0	Grade 1	Grade 2	Grade 3	Grade 4
발열(℃)	none	< 38.7	38.7 ~ 40	> 40.0	Fever with hypotension

14.3.3.2. 자/타각적 증상

자・타각적 증상의 정도는 연구자의 진료를 통하여 검사항목의 이상에 대한 기준이 있는 경우(WHO 이상반응 평가기준 등) 이들 기준에 따라 그 경중을 단계별로 나누어 평가하고, 기준 적용이 적절치 않을 경우는 Spilker 등의 3단계 분류법 및 다음의 일반 기준 분류법을 적용하여 평가한다. 그리고 기록상의 혼선을 방지하기 위하여 이상반응의 정도에 대한 분류는 다음과 같이 통일하도록 한다.

일반기준 분류법	없음(0)	경증(1)	중등도(2)	중증(3)	SAE(4) *
WHO	Grade 0	Grade 1	Grade 2	Grade 3	Grade 4
Spilker 분류법	None	Mild	Moderate	Severe	

*SAE(serious adverse event): 중대한 이상반응

- WHO 5 단계 분류법

Grade 0 :	어떤 구속 없이 정상적인 활동을 수행할 수 있는 상태
Grade 1 :	보행 및 일을 수행할 수 있으나 육체적으로 격한 활동이 제한되는 상태
Grade 2 :	보행 및 자기보호가 가능하나 깨어 있는 시간의 50% 이상 어떤 일을 수행할 수 없는 상태
Grade 3 :	오직 제한된 자기 보호가 가능하고 깨어 있는 시간의 50% 이상을 침대나 의자에 의존하는 상태
Grade 4 :	자기 보호가 불가능하고 의자에 전적으로 의존하고 상태

- Spilker 등의 3단계 분류법

Mild(1) : 처치가 필요치 않고 대상자의 정상생활(기능)을 크게 저해치 않는 경우

Moderate(2) : 대상자의 정상생활(기능)을 유의하게 저해하며, 처치가 필요할 수도 있으며 처치 후 회복되는 경우

Severe(3) : 심한 이상반응으로 고도의 처치가 필요하며, 후유증이 남는 경우

- 일반기준 분류법

Mild(1) : 쉽게 견딜 수 있는 이상반응

Moderate(2) : 일상 생활에 상당한 지장을 주는 이상반응

Severe(3) : 정상적인 일상 생활을 할 수 없을 정도의 이상반응

14.3.4. 시험 식품과의 인과관계

이상반응 발현 시 시험 식품과의 연관성 여부는 담당의사가 다음과 같이 분류하고, 필요 시 연구자의 견해를 부가한다.

(부록 2. 시험 식품과의 인과관계 판정기준 참조)

1. 명확히 관련이 있음
2. 관련이 있다고 생각됨
3. 관련이 있을 가능성이 있음
4. 관련이 없다고 생각됨
5. 명확히 관련이 없다고 생각됨
6. 불명

14.3.5. 보고방법

14.3.5.1. 이상반응의 보고

- 연구책임자는 연구담당자 및 연구대상자에게 시험용/대조용 식품 섭취 후 나타날 수 있는 모든 이상반응에 대하여 교육을 실시하고 섭취 후 나타나는 모든 현상에 대하여 보고하도록 교육을 시행한다.

- 시험용/대조용 식품 사용 후 전신적 또는 임상병리학적으로 나타나는 제반 증상에 대하여 종류, 발생시간, 정도, 처치, 치료약제, 경과, 시험용/대조용 식품과의 인과관계 등에 대한 기록 및 보관은 의약품 임상시험 관리기준에 준하도록 연구대상자 증례기록서에 기입한다.

• 연구책임자는 연구 결과보고시 연구 기간 중 발생된 모든 증상에 대하여 서술하고 평가를 실시하며, 연구 기간 중 “중대한 이상반응(Serious adverse event)” 발생 시에는 생명윤리위원회(IRB)에 보고하여 연구의 지속 또는 중단 여부를 결정하고, 신속 보고가 필요한 경우 의뢰자를 통해서 각 항에서 정한 기간 내에 신속히 IRB에 보고하도록 한다.

• 연구자에 의해 명확히 본제와의 관련성이 없음으로 판단된 이상반응의 추적조사 기간은 해당 대상자의 마지막 방문 후 14일(2주)로 정한다.

• 본제와의 인과관계가 있다고 판단된 추가적인 안전성 정보는 주기적으로 해당 약물이상반응이 종결(해당 약물이상반응의 소실 또는 추적조사의 불가 등)될 때까지 보고하여야 한다.

• 연구책임자는 연구를 시행함에 있어서 모든 제반 사항을 Declaration of Helsinki(부록 1)에 준하여 시행한다.

14.3.5.2. 신속보고

(1) 목적

연구자 및 기타 관계자로 하여금 중대한 약물이상반응에 대한 새롭고 중요한 정보를 알게 하기 위함이다.

(2) 보고 대상

① 중대하고 예상하지 못한 약물이상반응의 단일 증례

② 신속한 의사소통이 필요한 경우

각각의 상황에서는 적절한 의학적, 과학적 판단이 이루어져야 한다. 일반적으로 건강기능식품의 위험편익 평가에 영향을 줄 수 있는 정보, 건강기능식품 섭취에 변화를 줄 수 있는 정보 또는 아래에 예로 들은 상황들이 이에 해당한다.

a. 예상된 중대한 유해반응의 경우 임상적으로 중요하게 판단되는 발현률의 증가

b. 대상자에게 심각한 해를 끼칠 수 있는 경우

c. 새로 시행된 동물실험에서 중요한 안전성 정보를 알게 된 경우 (예 : 발암성)

(3) 보고 시기

연구 기간 중 중대하고 예상하지 못한 약물이상반응 등이 발생한 경우 연구책임자는 생명윤리위원회(IRB)에 보고하여 연구의 지속 또는 중단 여부를 결정하도록 한다.

① 사망을 초래하거나 또는 생명을 위협하는 예상하지 못한 약물이상반응은 신속히 보고하여야 한다. 연구책임자는 전화, 팩스, 문서를 통해 IRB의 규정에 따라 신속/정기 보고하고, 완전한 보고서를 제출하여야 한다.
② 다른 모든 중대하고 예상하지 못한 약물이상반응의 경우에는 이 사실을 보고 받거나 알게 된 날로부터 IRB의 규정에 따라 보고한다.

(4) 보고를 위한 최소기준
① 이상반응의 보고를 위한 최종 기술 및 평가에 대한 정보는 상기 보고시기 내에 얻지 못할 수 있다. 그러나 정해진 상기 보고시기 내에 초회 보고를 하여야 한다.
② 추적 조사 정보는 상세히 조사, 제출되어 활동될 수 있도록 한다.

(5) 보고 방법
- 이상반응 신속 보고 시에는 IRB의 중대한 이상반응 보고서 양식을 이용하며, 신속보고 시점에서 모든 정보를 얻을 수는 없더라도 가능한 많은 정보를 얻기 위해 최대한 노력을 기울여야 한다.

14.3.5.3. 예상하지 못한 중대한 약물이상반응 발생 시 조치 사항

본 연구 기간 중 연구책임자, 연구담당자는 연구대상자의 안전에 만전을 기하여야 하며, 예상하지 못한 중대한 약물이상반응 발생 시에는 연구를 중지하고 신속하고 적절한 조치를 취하여 이상반응을 최소화하여야 한다.
연구 중 “예상하지 못한 중대한 약물이상반응” 발생 시 각 담당자의 의무는 다음과 같다.

(1) 연구책임자의 의무
연구책임자는 연구 중 예상하지 못한 중대한 약물이상반응이 발생한 때에는 즉시 생명윤리위원회(IRB) 및 의뢰자에게 보고하고 별도의 지시가 있을 때까지 해당 시험 식품에 대한 연구의 일부 또는 전부를 중지하여야 한다.

(2) 연구담당자의 의무
연구담당자는 연구 실시 중에 예상하지 못한 중대한 약물이상반응 등이 발생한 경우에는 즉시 연구책임자 및 의뢰자에게 보고하여야 한다.

(3) 생명윤리위원회(IRB)의 의무
생명윤리위원회(IRB)는 예상하지 못한 중대한 약물이상반응이 나타난 경우에는 연구의 일부 또는 전부에 대하여 중지명령 등 필요한 조치를 연구책임자에게 하여야 한다.

(4) 의뢰자의 의무

의뢰자는 연구책임자, 연구담당자로부터 예상하지 못한 중대한 약물이상반응 보고를 받은 경우 이상반응보고서에 연구책임자, 연구담당자로부터 제출 받은 보고서 사본을 식품의약품안전처장에게 제출하여야 한다.

사망을 초래하거나 생명을 위협하는 경우에는 의뢰자가 이 사실을 보고받거나 알게 된 날로부터 7일 이내에 보고하고, 상세한 정보를 최초 보고일로부터 8일 이내에 추가로 보고하여야 한다. 그 밖의 중대하고 예상하지 못한 약물이상반응의 경우 의뢰자가 해당 사실을 보고받거나 알게 된 날부터 15일 이내 제출하여야 한다.

15. 자료분석과 통계적 방법

15.1. 결과분석의 일반적 원칙

본 연구의 대상자로부터 얻어진 자료는 크게 Safety Set 분석, FAS(Full Analysis Set) 분석과 PPS(Per Protocol Set) 분석의 세 가지 형태로 이루어진다.

Safety Set 분석은 시험 식품을 한번이라도 섭취한 대상자 집단을 대상으로 이루어진다.

본 연구에 무작위 배정을 받은 모든 대상자를 분석 대상으로 하는 원칙(ITT)에 따라 FAS 분석은 시험 식품을 1회 이상 섭취한 후 유효성 평가를 1회 이상 시행한 대상자며 주요 선정기준 위반에 해당되지 않는 집단을 대상으로 이루어진다.

PPS 분석은 FAS 중에서 연구를 종료하고, 연구 결과에 영향을 미치는 중대한 위반사항(선정/제외기준 위반 등)이 없는 대상자 집단을 대상으로 이루어진다.

유효성에 대한 자료는 PPS 분석을 주 분석으로 하고, FAS 분석을 추가적으로 실시한다. 영양분석 및 인구학적에 대한 자료는 PPS 분석을 주 분석으로 하고 안전성에 대한 자료는 Safety Set 분석을 주 분석으로 실시한다.

결측치가 발생한 경우, 결측치가 발생한 시점을 기준으로 가장 최근에 얻어진 자료를 이용하여 보정(LOCF)하여 분석한다.

본 연구에서 얻은 자료는 적당한 기술통계량으로 평균(Mean)과 표준편차(Standard deviation)를 산출하여 제시하며, 차이(Difference)에 대한 유의성은 양측검정으로 $p<0.05$ 수준에서 검증한다.

유효성 평가변수에 대한 분석방법으로 평가변수와 연관이 있는 대상자의 기저특성(공변량,

대상자가 시험 식품을 투여하기 전에 관찰되거나 측정된 일차 유효성 평가변수에 영향을 미칠 것으로 예상되는 변수)를 고려하여 분석하는 ANCOVA 분석을 실시 할 수 있다.
Sub-set(group) analysis(소집단 분석)은 무작위배정이전 또는 치료시작 전의 대상자의 초기 특성(예. 스트레스 반응척도(SRI), 기타 기저특성(baseline characteristics))으로 범주화하여 추가분석을 실시할 수 있다.

연속형 자료의 경우 분포에서 비정상적으로 벗어난 값에 대해 이상치로 정의하고, 분석에서 제외될 수 있다.

15.2. 인구통계학적 기초자료

연구대상자의 인구통계학적 혹은 임상학적 특성을 파악하기 위하여 연구 전 연구대상자의 특성에 대하여 기술한다. 인구통계학적 자료 및 건강 상태에 관하여 자료에 따라서 적당한 기술통계량들을 구하고, 연속형 자료는 군별 평균과 표준편차를 제시한다. 군별 비교를 위하여 Two-sample t-test를 실시하고 유의확률 값을 제시하였다. 범주형 자료는 각 수준별로 빈도와 비율을 제시하고 독립성검정을 위한 Chi-square test 혹은 Fisher's exact test를 실시하고 유의확률 값을 제시한다.

15.3. 유효성 변수에 대한 분석

WBC 수, NK cell activity(2.5:1, 5:1, 10:1, 50:1), IFN-ɣ, IL-2, IL-6, IL-12, TNF-α, IgG1, IgG2, IgM 연구대상자 자신에 의한 개선도 평가에 대한 섭취 전후 변화의 정도는 Paired t-test를 실시하고, 각 시점에서의 군간 변화의 정도는 ANCOVA와 Two sample t-test를 실시하여 통계적으로 유의한 차이가 있는지 평가한다. 단, 정규성이 심각하게 위배되는 경우 로그변환방법(logarithm transformation) 등의 변수변환을 실시한 후 검정하거나 비모수검정법인 Wilcoxon rank sum test를 실시할 수 있다.

15.4. 안전성 변수에 대한 분석

15.4.1. 이상반응

시험 식품 섭취 후 발생한 이상반응(treatment-emergent adverse events, TEAEs)은 MedDRA preferred term에 따라 Coding하며, 시험 식품 섭취 후 발생한 모든 이상반응을 도표화 한 후 발생률을 산출하여 평가한다. 각 군간 이상반응이 발생한 연구대상자의 비율을 계산하고 카이제곱검정(Chi-square test) 또는 피셔의 정확검정(Fisher's exact test)을 이용하여 비교 분석한다.

15.4.2. 임상병리검사

섭취 전(방문1: week - 2)과 섭취 후(방문4: week 8)의 임상병리검사 결과를 비교하여 어떤 변화가 있는지 관찰된 결과치를 제시한다.

혈액학적 및 혈액화학적 검사치와 같이 연속형(Continuous type)자료의 군내 비교는 Paired t- test를 이용하고, 군간 비교는 two- sample t- test를 이용하여 분석한다. 요검사의 일부 측정 변수의 경우는 정상과 비정상으로 나누어 McNemar test를 실시하여 군내 차이를 비교한다.

15.4.3. 활력징후***(***맥박***,*** 혈압***)*** 및 체온***,*** 체중

활력징후(맥박, 혈압) 및 체온, 체중 검사치에 대하여 섭취 전과 후의 차이에 대한 군내 비교는 Paired t- test를 이용하고, 군간 비교는 Two sample t- test를 이용하여 분석한다.

15.5. 중간분석

본 연구에서는 적용하지 않는다.

15.6. 기타 관찰항목 분석

연구대상자의 섭취 전, 중, 후에 얻어지는 관찰항목(식이조사 등)은 적당한 기술통계량들을 구하고, 연속형 자료는 군별 평균과 표준편차를 제시한다. 군별 비교를 위하여 Two- sample t- test를 실시하고 유의확률 값을 제시한다. 범주형 자료는 각 수준별로 빈도와 비율을 제시하고 독립성검정을 위한 Chi- square test 혹은 Fisher’s exact test를 실시하고 유의확률 값을 제시한다.

16. 예측 부작용 및 주의사항과 조치

바이오게르마늄 효모 1.2g을 2개월간 섭취시킨 이전 인체적용시험결과에서 혈액학적 검사 및 혈액 화학적 검사에서 특별한 이상반응이나 부작용은 발생하지 않았다. 특히, 신장과 간의 기능과 관련된 ALT, AST, ALP, BUN, 크레아틴, 총빌리루빈 및 빈혈지표인 헤모글로빈, 헤마토크릿, 적혈구에도 어떠한 변화가 나타나지 않는 것으로 보아 바이오게르마늄 효모의 1일 1.2g의 섭취는 안전한 것으로 사료된다.[12] 또한 본 원료는 1990년대부터 판매되어 온 제품으로 20년 넘게 유통되었고 크게 문제된 사례가 없던 것으로 알려져 있다. 다만, 천연물 소재의 추출물 복용 시 있을 수 있는 경미한 소화불량이나 복통이 발생할 수 있으며, 체질에 따라 발효균주인 효모에 알레르기가 유발될 수 있으므로 주의하여야 한다. 연구대상자에게 이상반응이 발생할 경우 연구자 또는 연구담당자에게 보고하도록 교육을 시행한다.
바이오게르마늄 효모의 인체적용시험은 임상적으로 이상이 없는 성인을 대상으로 시험하였기 때문에 임산부와 12세 이하 어린이에 대한 섭취 시 주의가 요구된다. 또한, 유아의 손에 닿지 않는 곳에 보관하고, 오용 및 남용을 피하고 품질을 유지하기 위해 다른 용기에 넣지 말아야 한다.

17. 중지 및 탈락기준

연구에 참여하는 모든 연구대상자의 연구 완료 여부를 기록하고, 시험 식품 섭취나 관찰이 중단된 경우에는 그 이유를 기록한다. 연구가 진행 중인 대상자에 대하여 연구를 중단할 수 있는 경우는 다음과 같다.

① 선정기준/제외기준에 위배된 경우
② 면역 관련 건강기능 식품 및 의약품을 섭취하는 경우
③ 중대한 이상반응(Serious Adverse Events) 혹은 이상반응(Adverse Events)으로 인하여 연구대상자가 연구 중단을 요구하는 경우
④ 섭취 전 검사에서 발견치 못한 전신질환이 발견된 대상자
⑤ 연구 기간 중 만족스럽지 못한 치료 효과로 인하여 대상자(또는 법정 대리인)가 시험 중단을 요구하는 경우
⑥ 연구대상자(또는 법정 대리인)가 연구 참가 동의를 철회한 경우
⑦ 연구대상자의 추적이 안되는 경우
⑧ 연구대상자에게 시험 식품을 섭취하는데 문제가 있는 경우
⑨ 연구 기간 동안 담당의사의 지시 없이 연구 결과 판정에 영향을 미칠 수 있는 약물 및 식품 등을 투여한 경우
⑩ 연구자의 판단에 의해 연구의 진행이 적합하지 못하다고 판단되는 경우
⑪ 임신이 확인되는 경우

연구자가 어떤 사유로든 연구대상자에게 최선이라고 결론을 내린 경우에는 언제든지 연구대상자를 탈락시켜야 한다. 계획서 위반이 대상자의 안전성에 유의한 위험이 되지 않으면, 계획서 위반 때문에 대상자를 탈락시키지 말아야 한다.

연구대상자는 언제든지 어떤 사유로든 시험을 자발적으로 중단할 수 있다. 연구대상자가 중단할 의사를 표현한 경우, 방문을 하지 않은 경우, 또는 어떤 사유로든 추적조사에 실패한 경우 탈락으로 간주된다.

조기 탈락이 발생하게 되면, 연구자는 연구대상자가 시험에서 조기 탈락하게 된 일차 사유를 결정하여 이 정보를 증례기록서 '시험종결'에 기재하여야 한다.

연구대상자의 정해진 방문일의 window period 3배수까지 추적을 진행하여 추적에 실패한 연구대상자는 탈락시킬 수 있다. 추적조사에 실패한 연구대상자(즉, 중단 의사를 표시하지 않고 연구 방문에 나타나지 않아서 대상자의 상태가 불분명한 대상자)에 대해서는 연구대상자에게 연락하기 위해 취한 조치(예: 전화한 날짜, 등기 우편 등)를 근거 문서에 기록함으로써 "책임을 다했음"을 보여야 한다.

17.1. 순응도와 연구계획서 위반에 대한 처리

본 연구의 연구책임자와 연구담당자는 연구계획서의 위반이 발생하지 않도록 계획서에 대해 충분히 숙지하고 철저히 이행하여야 한다. 본 연구에서 연구대상자는 처방된 시험 식품을 지시된 사용방법을 준수하여 사용하도록 교육한다. 시험 식품의 정확한 사용과 방문 일정 준수를 위하여 연구자는 연구계획서에서 정한 방문 계획에 따라 방문할 수 있도록 적절한 조치, 예컨대 차기 방문시간에 대한 서면 통보나 전화 모니터링 등을 필히 실시해야 한다. 반면, 부득이하게 발생된 계획서 위반 사항에 대해서는 다음에 따라 처리한다.

중대한 시험계획서 위반의 경우, 예컨대, 대상자 중지/탈락 기준에 해당하거나, 대상자 선정/제외기준 위반, 동의서 미취득, 섭취하지 않은 경우 등 대상자의 안전과 연구결과에 중대한 영향을 미칠 수 있는 위반의 경우 해당 대상자를 연구자와 협의 후 연구에서 탈락처리(Drop-out) 할 수 있다.

기타 경미한 위반 사항은 위반 또는 지연 정도와 사유를 정확히 기재하고 결과분석 시 연구에 영향을 주었는지 고찰한다.

18. 연구대상자의 위험과 이득

본 연구는 일반인을 대상으로 시행되는 연구로 위험은 높지 않을 것으로 생각된다. 본 시험용 식품은 인체에 안전한 원료이지만 섭취했을 경우 개인의 건강상태에 따라 소화불량 또는 알레르기 등의 예측하지 못한 이상반응이 나타날 수 있으나, 이는 일반적으로 건강기능식품 섭취 시 개인의 건강 상태에 따라 경험 할 수 있는 수준의 위험이다. 이와 같이 본 연구 진행과 관련되어 발생할 수 있는 위험은 경미하거나 예측 가능한 것으로 판단된다. 연구대상자가 시험 식품 섭취에 따라 위와 같은 증상을 보이거나 불편함을 호소할 경우 연구대상자는 즉시 시험 식품 섭취를 중단하고 본 연구 담당 의사에게 진료를 받고 담당의사의 판단에 의해 섭취 중단 외에 추가적인 치료가 필요한 경우 '피해자보상에 관한 규약' 및 본 연구를 위해 가입한 임상연구 보험 증권에 따라 보상을 받게 된다. 연구대상자가 연구에 참여하는데 있어서 직접적인 이득은 없다. 다만 연구대상자가 연구에 참여하는데 있어서 일정 금액의 교통비를 제공한다. 연구 참여를 완료하면 교통비 등의 실비로 연구대상자에게 총 200,000원이 지급된다. 중도 포기 및 탈락의 경우 방문(방문1~4)한 횟수(방문 당 50,000원)에 따라 교통비를 지급한다. 연구대상자가 제공하는 정보는 바이오게르마늄 효모의 면역력 증진 효과에 대한 이해를 증진하는데 도움이 될 것이다.

19. 연구대상자 안전대책 및 개인정보보호대책

19.1. 연구 실시기관

연구 실시기관의 장은 각 연구 단계별로 해당 연구의 실시에 필요한 실험실 및 전문인력을 갖추고, 해당 연구를 적정하게 실시할 수 있도록 준비에 완벽을 기해야 한다.

19.2. 연구계획서의 승인 및 수정

연구의 승인을 얻거나 승인 받은 연구를 변경하여 실시하고자 하는 경우, 연구 단계별로 계획서 또는 변경 계획서에 대하여 생명윤리위원회(IRB)의 승인을 받는다. 승인 이전에 대상자를 연구에 참여 시킬 수 없다.

19.3. 연구계획서의 숙지

본 연구는 헬싱키 선언에 입각하여 대상자의 권리와 복지를 염두에 두고 준비된 것으로서, 연구책임자, 연구자가 연구계획을 정확히 분석 및 숙지하고, 연구대상자의 문제점을 적극적으로 대응한다.

19.4. 연구의 동의

연구에 들어가기 전에 연구대상자에게 연구내용 및 시험용 식품의 효과, 이상반응 및 안전성에 대한 모든 사항을 설명한 후 연구대상자 자신이 자발적으로 본 연구에 참여하겠다는 동의서를 받고 연구에 들어간다.

19.5. 적합한 연구대상자의 선정

연구실시기관의 직원(교내 및 원내 직원)이 본 연구에 자발적으로 참여를 원하는 경우, 교내 및 병원 직원을 취약한 환경의 대상자(Vulnerable subject)로 규정하고 취약한 대상자 보호대책 방안에 따라 본 연구에 의해 어떠한 불이익도 받지 않도록 한다. 본 연구 참여에 앞서 취약한 대상자(교내 및 원내 직원)에게 취약한 대상자 보호대책 방안에 대해 충분히 설명한 후 취약한 대상자(교내 및 원내 직원)의 동의를 얻는다. 취약한 대상자 보호대책 방안은 다음과 같다.

① 대상자 모집 목적으로 연구자가 취약한 대상자와 직접적으로 상호작용을 하지 않는다.
② 연구 모집이나 동의 과정은 취약한 대상자의 상사가 없는 상태에서 진행하도록 한다.
③ 취약한 대상자가 연구에 참여하지 않기로 선택할 수 있고, 그러한 결정이 고용이나 직무 평가에 영향을 미치지 않음을 보장한다.
④ 취약한 대상자가 부당한 영향이나 강제를 받지 않을 것이고, 취약한 대상자의 사생활이 존중될 것이라는 점을 보장한다.
⑤ 연구 참여를 거절했다는 사실을 취약한 대상자의 상사에게 알리지 않는다.

19.6. 연구의 진행 점검

연구책임자는 의뢰자에게 주기적으로 이상반응, 연구진행, 상황, 결과 등에 대하여 보고하며, 의뢰자는 주기적으로 연구 진행 상황에 대하여 점검을 시행할 수 있다.

19.7. 연구 실시기관의 모니터링

연구대상자의 권리와 복지 보호, 보고된 연구 관련 자료가 근거문서와 대조하여 정확하고, 완전하며, 검증이 가능한지 여부 확인, 연구의 승인된 계획서, 의약품 임상시험관리기준 및 의약품 등 안전에 관한 규칙 제30조의 규정에 따라 수행되는지의 여부 확인을 위하여 모니터링을 시행한다.

연구에 대한 모니터링은 게란티제약㈜으로부터 연구 업무를 수탁받은 네오뉴트라㈜ 모니터 요원의 정기적인 연구기관 방문과 전화를 통해서 이루어 질 것이다. 방문 시 모니터요원은 기본적으로 연구대상자기록 원본, 시험 식품 관리 기록 및 기본문서 등을 확인한다. 또한, 모니터요원은 연구 진행과정을 잘 살피고, 문제가 있을 경우 연구자와 상의한다.

이들 방문의 적절한 시간은 연구자와 모니터요원과 협의하여 시행될 것이다. 연구자는 ICH- GCP에 정의된 것과 같이, 모니터요원이 증례기록서(CRF)에 기입된 자료들을 확인할 수 있는 연구대상자의 원 자료들(Source documents: 병원 또는 개인 차트, 실험실 결과 기록, 예약 기록 등)을 볼 수 있도록 해주어야 한다.

19.8. 연구대상자의 개인정보와 비밀유지

연구대상자의 신원을 파악할 수 있는 기록은 비밀로 보장될 것이며, 연구의 결과가 출판될 경우에도 대상자의 신원을 비밀상태로 유지한다. 구체적인 내용은 다음과 같다.

본 연구에 관련된 의뢰자, 모니터 및 점검자는 본 연구의 모니터링과 점검 및 진행사항 관리를 위한 목적으로 대상자의 기록을 열람할 수 있다. 연구자는 본 연구의 계약이 체결됨과 함께 의뢰자 또는 연구 수탁기관의 모니터요원 및 점검자가 근거문서와 증례기록서 기록을 검증하기 위하여 해당 문서를 검토하거나 복사할 수도 있음을 숙지하여야 한다. 이러한 정보들은 기밀로 보관되어야 하며, 증례기록서 등 연구와 관련된 모든 서류에는 연구대상자의 이름이 아닌 연구대상자 식별코드(일반적으로 대상자 이니셜)로 기록하고 구분한다. 또한 본 연구에서 수집되는 개인정보는 타 연구나 타 연구자에게 제공하지 않는다. 연구책임자인 연세대학교 식품영양학과 이종호 교수는 연구와 관련된 각종 자료 및 기록에 대한 정보들을 위조, 변조 방지하기 위해 삼성관 616호의 잠금장치가 있는 캐비닛에서 안전하게 보관한다. 연구문서는 결과보고서가 작성 완료되어 제출된 후 5년 동안 보관하도록 한다.

개인 정보관련: 대상자가 별도로 제공한 통장사본, 신분증사본(개인정보제공 및 활용동의서 내 서명 포함)은 연구를 위한 목적으로 사용되지 않는다. 이 정보는 실험 참여보수 지급을 위한 것으로 봉투에 바로 밀봉하여 연세대학교 산학 협력단에 원본이 제공되며 본 연구팀에서는 보관하지 않는다. 산학협력단에 제공된 정보는 관련 법률에 의해 보관될 것이다. 연구팀이 입금확인을 위해 이름 및 전화번호 등을 보관할 수는 있으나, 입금 확인 후 바로 폐기한다.

19.9. 시험 식품 관리

- 시험 식품은 연구책임자의 지시(처방) 없이는 사용할 수 없도록 한다.
- 의뢰자는 시험 식품 등을 연구책임자와 협의하여 연구책임자 또는 연구담당자에게 교부하여야 하며 인수증을 받아 보존하여야 한다. 이 때 시험 식품에는 "인체적용시험용"이라는 표시가 있어야 한다.
- 연구자는 시험 식품 등이 연구 이외의 목적으로 사용되지 않도록 보관, 관리하여야 한다.
- 의뢰자는 연구 실시 중 시험 식품 등의 수량 및 보관상태 등을 확인하고 연구가 적절히 진행될 수 있도록 조치하여야 한다.
- 의뢰자는 연구의 중지 및 종료 또는 연구자가 계획서에 따라 연구를 실시하지 않는 경우 미사용 시험 식품 등을 회수하여 폐기하도록 한다. 이 때 연구책임자 또는 연구담당자는 연구책임자와 협의 후, 미사용 시험 식품을 의뢰자에게 반납하고 그 반납증을 보존하여야 한다.

19.10. 인체유래물 보관 및 폐기 방법

본 연구에서 수집되는 혈액 총 75mL(방문1 11mL, 방문2 18mL, 방문3 19mL, 방문4 27mL)는 전혈, 혈장, 혈청, 적혈구로 분리하여 –80도에 보관하고, 소변 총 10mL(방문1 5mL, 방문4 5mL)는 - 20도에 보관을 한다. 연구의 목적으로 수집된 검체는 직접적으로 신원을 확인할 수 없는 방법으로 보관되고, 연구 과정에서 획득된 임상연구용 검체는 향후 본 연구 이외의 다른 학술연구에 이용될 수 있다. 본 연구 분석에 사용 후 남은 혈액은 인체유래물 동의서에 의해 연구대상자가 지정한 기간까지 보관할 예정이며, 본 연구 직후 폐기를 원하는 경우 또는 지정기간이 경과한 이후에는 혈액 의료 폐기물로 분류 후 의료 폐기물 전용 용기에 담아서 폐기한다.

19.11. 이상반응 발생 시 조치

이상반응 발생 또는 의학적인 문제가 있을 시 본 연구 참여연구원인 세브란스병원 내분비내과 임상교수(신동엽)로부터 필요검사 및 치료를 받을 수 있도록 관리한다. 중대한 약물이상반응 발생시는 시험을 중지하고, 15.3.5 항의 이상반응 발생 시 조치사항에 따라 신속하고 적절한 조치를 취한다.

20. 연구수행 일정표

Period		Screening[1]	Active Treatment		
Visit		1	2	3	4
Week		- 2	0	4	8
Window period (day)[1]				± 7	± 5
서면동의서		√			
인구학적 조사[2]		√			
병력/약물 투여력 조사[3]		√	√		
활력징후(혈압, 맥박) 측정		√		√	√
체온 측정		√	√	√	√
이학적검사		√		√	√
임상병리검사[4]		√			√
임신반응검사[4]		√			√
식이조사[5]			√	√	√
스트레스 반응척도(SRI)[6]			√		
상기도 감염 발생여부 조사		√			
면역 관련 유전자 검사[7]			√		
신체계측	신장	√			
	체중	√		√	√
유효성 평가	NK cell activity[9]		√	√	√
	WBC 수[9]	√		√	√
	IFN- γ[8][9]		√	√	√
	IL- 2, 6, 12[8][9]		√	√	√
	TNF- α[8][9]		√	√	√
	IgG1, IgG2, IgM[8][9]		√	√	√
	연구대상자 자신에 의한 개선도 평가			√	√
연구대상자 적합성 평가		√	√		
무작위배정			√		
시험/대조 식품 처방			√	√	
병용약물 및 병용요법 확인				√	√
이상반응 확인				√	√
순응도 확인				√	√

1) 스크리닝 후 2주일 이내 방문2가 시행되어야 한다. 방문3의 방문일은 지정된 날짜 전·후 7일을 허용하며, 방문4의 방문일은 지정된 날짜 전·후 5일을 허용한다.

2) 방문1에 연구대상자의 이니셜, 성별, 생년월일, 연령, 면역질환 관련 증상 가족력, 음주여부, 흡연

여부, 운동여부 등을 조사하여 기록한다.

3) 방문1에 스크리닝 시점을 기준으로 3개월 이내의 외과적 수술력을 포함한 병력과 4주 이내의 약물 투여력을 조사하여 기록한다.

4) 연구대상자는 채혈하기 전날 12시간 금식상태로 내원하여 다음의 항목을 검사한다. 연구자의 판단에 따라 비정상적인 결과에 대한 재검사를 시행할 수 있다.

- 혈액학적 검사(7종) : WBC, RBC, Hemoglobin, Hematocrit, Platelet count, Lymphocyte, Monocyte
- 혈액화학적 검사(10종): AST(GOT), ALT(GPT), Total protein, Glucose, Total cholesterol, BUN, Creatinine, Uric acid, Ca, P
- 소변검사(10종): SG, pH, WBC(Leukocyte), Nitrite, Protein, Glucose, Ketone, Urobilinogen, Bilirubin, Erythrocyte
- 임신반응검사: urine HCG(방문1과 방문4에서 가임기 여성만 해당)

5) 연구대상자는 방문2에서는 하루 전날 섭취한 식사를 24시간 회상법으로 연구담당자가 기록하며, 방문2, 3에 연구대상자에게 식이조사지를 배부하여 방문3, 4에 회수하여 확인한다.

6) 방문2에 스트레스 반응 척도(SRI)를 이용하여 연구대상자에게 평상시의 스트레스 자각 정도에 관하여 평가한다.

7) TNF-a G308A 유전자 다형성 분석을 위해 방문2에 약 2ml을 채혈한다.

8) 혈액 채취 후 혈청을 분리하여 분석 전까지 -80℃에 냉동보관 후 분석한다.

9) 연구대상자는 채혈하기 전날 12시간 금식상태로 내원하여 검사한다.

21. 참고문헌

1) Jao S-W, Lee W, Ho Y-S. Effect of germanium on 1, 2-dimethylhydrazine-induced intestinal cancer in rats. Diseases of the Colon & Rectum. 1990;33:99-104.
2) Yang F, Jin H, Pi J, Jiang J-h, Liu L, Bai H-h, et al. Anti-tumor activity evaluation of novel chrysin–organogermanium (IV) complex in MCF-7 cells. Bioorganic & Medicinal Chemistry Letters. 2013;23:5544-51.
3) Yang MK, Kim YG. Protective role of germanium-132 against paraquat-induced oxidative stress in the livers of senescence-accelerated mice. Journal of Toxicology and Environmental Health Part A. 1999;58:289-97.
4) Wu Z, Chen X, Yang K, Xia T. Studies on the hydroxyl free radical-scavenging effect of combined selenium and germanium. Wei Sheng Yan Jiu (Journal of Hygiene Research). 2001;30:208-10.
5) Aso H, Suzuki F, Ebina T, Ishida N. Antiviral activity of carboxyethylgermanium sesquioxide (Ge-132) in mice infected with influenza virus. Journal of Biological Response Modifiers. 1989;8:180-9.
6) DiMartino M, Lee J, Badger A, Muirhead K, Mirabelli C, Hanna N. Antiarthritic and immunoregulatory activity of spirogermanium. Journal of Pharmacology and Experimental Therapeutics. 1986;236:103-10.
7) Lee J, Kim K, Yoon M, Lee J, Kim C, Sim S. Anti-inflammatory effect of germanium-concentrated yeast against paw oedema is related to the inhibition of arachidonic acid release and prostaglandin E2 production in RBL 2H3 cells. Autonomic and Autacoid Pharmacology. 2005;25:129-34.
8) Suzuki F, Brutkiewicz R, Pollard R. Importance of T-cells and macrophages in the antitumor activity of carboxyethylgermanium sesquioxide (Ge-132). Anticancer Research. 1985;5:479-83.
9) Badger AM, Mirabelli CK, DiMartino M. Generation of suppressor cells in normal rats by treatment with spirogermanium, a novel heterocyclic anticancer drug. Immunopharmacology. 1985;10:201-7.
10) Nakamura T, Takeda T, Tokuji Y. The oral intake of organic germanium, Ge-132, elevates α-Tocopherol levels in the Plas-ma and modulates hepatic gene expression profiles to promote immune activation in mice. International Journal for Vitamin and Nutrition Research. 2015;84:0183-95.
11) Hirayama C, Suzuki H, Ito M, Okumura M, Oda T. Propagermanium: a nonspecific immune modulator for chronic hepatitis B. Journal of Gastroenterology. 2003;38:525-32.
12) 박재현, 김형수, 정세진, 유미현, 이종권, 엄준호, 변정아, 오혜영. 기능성식품이 면역계에 미치는 영향에 관한 연구. 식품의약품안전청보고서 제6권 The Annual Report of KFDA. 2002;Vol.6:355-364.
13) 박재현, 김형수, 정세진, 유미현, 이종권, 엄준호, 변정아, 오혜영. 기능성식품이 면역계에 미치는 영향에 관한 연구(II). 식품의약품안전청보고서 제7권 The Annual Report of

KFDA. 2003;Vol.7:258-262.
14) Aso H, Suzuki F, Yamaguchi T, Hayashi Y, Ebina T, Ishida N. Induction of interferon and activation of NK cells and macrophages in mice by oral administration of Ge-132, an organic germanium compound. Microbiol Immunol. 1985;29(1):65-74.
15) Aso H, Suzuki F, Ebina T, Ishida N. Antiviral activity of carboxyethylgermanium sesquioxide (Ge-132) in mice infected with influenza virus. Journal of Biological Response Modifiers. 1989;8:180-9.
16) Lee S-H, Oh S-W, Rho S-N, Lee B-H, Lee H-J, Jin D-K. Effects of Germanium-fortified Yeast on the Serum Lipids and Immune Cell Subset. Journal of the Korean Society of Food Science and Nutrition. 2006;35:683-9.
17) Lee S-H, Rho S-N, Sohn T-U. Efficacy study of activation on macrophage in germanium-fortified Yeast. Applied Biological Chemistry. 2005;48:246-51.
18) Joo SS, Won TJ, Lee YJ, Kim MJ, Park S-Y, Lee SH, et al. Effect of Geranti Bio-Ge Yeast, a Dried Yeast Containing Biogermanium, on the Production of Antibodies by B Cells. Immune Network. 2006;6:86-92.
19) Ishiwata Y, Suzuki E, Yokochi S, Otsuka T, Tasaka F, Usuda H, Mitani T. Studies on the antiviral activity of propagermanium with immunostimulating action. Arzneimittel-Forschung. 1994 Mar;44(3):357-61.
20) 강종구, 강신석, 이종성, 정재황, 이혜영, 이상철. 유기게르마늄을 함유한 효모 게란티 (Geranti)가 항암작용 및 면역증강에 미치는 영향. 충북대학교 보고서, 1995.
21) 바이오톡스텍. 게란티-골드: 대식세포활성화효과에 대한 연구. 바이오톡스텍 보고서 (시험번호: B03180), 2003.
22) 바이오톡스텍. 게란티-골드: 면역활성시험. 바이오톡스텍 보고서 (시험번호: B02154-1), 2003.
23) Sung-Hee Lee, Kyeong-Nam Oh, Sook-Nyung Rho, Bok-Hee Lee, Hyun-Joo Lee, Dong-Kyu Jin. Efficacy study of activation on macrophage in Germanium-fortified Yeast. J. Korean Soc. Appl. Chem. 2005;48(3):246-251.
24) 주성수, 원태준, 이용진, 김민정, 박소영, 이성희, 황광우, 이도익. B세포의 항체 생산에 대한 게란티 바이오게르마늄 효모의 영향. Immune network(대한면역학회지). 2006;6(2):86-92.
25) 백대헌, 정진욱, 손창욱, 강종구. 게르마늄 강화효모의 마우스에서의 암세포 억제 및 대식세포, NK 세포, B 세포의 활성화에 관한 연구. Kor. J. Microbiol. Biotechnol. 2007;35(2):118-127.
26) 바이오톡스텍. 게란티-골드: Sacrcoma-180 cell에 의해 유도된 고형암에 대한 성장 억제 효과 시험. 바이오톡스텍 보고서 (시험번호: B03138), 2003.
27) 바이오톡스텍. 게란티-골드: 용혈반형성세포수측정시험. 바이오톡스텍 보고서 (시험번호: B03138), 2003.
28) 주성수, 원태준, 이용진, 김민정, 박소영, 이성희, 황광우, 이도익. B세포의 항체 생산에 대한 게란티 바이오게르마늄 효모의 영향. Immune network(대한면역학회지). 2006;6(2):86-92.

29) Sung-Hee Lee, Kyeong-Nam Oh, Sook-Nyung Rho, Bok-Hee Lee, Hyun-Joo Lee, Dong-Kyu Jin. A study on effects of Germanium-fortified Yeast on serum lipids and Immune cell subset : 게르마늄강화효모가 혈청지질 및 면역세포에 미치는 영향. 한국식품영양과학회지. 2006;35(6):683-689.
30) Tanaka N, Ohida J, Ono M, Yoshiwara H, Beika T, Terasawa A, Yamada J, Morioka S, Mannami T, Orita K. Augmentation of NK activity in peripheral blood lymphocytes of cancer patients by intermittent Ge-132 administration. Gan to kagaku ryoho (Cancer & Chemotherapy). 1984;11(6):1303-6.
31) Takahashi I, Fukumoto M, Inagaki N, Ueda I, Nishimura M, Aoyama S, Oda Y, Ohmoto E, Takaoka K, Lai M, et al. Effects of interferon and its inducers on neutrophil chemiluminescence. Gan to kagaku ryoho (Cancer & Chemotherapy). 1984;11(7):1439-43.
32) Sung Hee Lee, Sangdoo Ahn, Sook-Nyung Rho, Tsang-Uk Sohn. A study on preparation and binding properties of germanium-fortified yeast. The Korean Society for Applied Biological Chemistry(한국응용생명화학회지). 2005;48(4):382-387.
33) Sung-Hee Lee, Sang Kwang Lee, Hyun Joo Lee, Yong Sub Yi, Eun Woo Park. Study on Identification and Purification of Germanium-fortified Yeast. The Korean Society for Applied Biological Chemistry(한국응용생명화학회지). 2006;49(1):55-59.
34) So-Yeun Kim, Myoung-Hee Kim, Hee-Gweon Woo, Bo-Hye Kim, Tsang-Uk Sohn, Jin-Wook Jung, Dae-Heoun Baek. Qualitative Analysis of GeO2 in Germanium-Fortified Yeast. Korean journal of microbiology and biotechnology(생명공학회지). 2007;35(2):163-72.
35) Jong-Sung Lee, Jong-Il Park, Sun-Hee Kim, Sun-Hee Park, Seong-kwi Kang, Cheol-Beom Park, Tsang-Uk Sohn, Ja Young Jang, Jong-Koo Kang, Yun-Bae Kim. Oral single and repeated dose toxicity studies on Geranti Bio-Ge Yeast, organic germanium fortified yeasts, in rats. The journal of toxicological sciences. 2004;29(5);541-553.
36) Jong-Sung Lee, Jong-Il Park, Sung-Hee Kim, Hye-Young Lee, Zai-Zhi Hwang, Cheol-Beom Park, Tsang-Uk Sohn, Sunhee Shin, Jong-Koo Kang, Yun-Bae Kim. Oral single and repeated dose toxicity studies on Geranti Bio-Ge Yeast, organic germanium fortified yeasts, in dogs. The journal of toxicological sciences. 2004;29(5);555-569.
37) 바이오톡스텍. 게란티 효모 G: 랫드를 이용한 경구 단회투여 독성시험. 바이오톡스텍 보고서 (시험번호: B02147), 2002.
38) 바이오톡스텍. 게란티 효모 G: 비글견을 이용한 경구 단회투여 독성시험. 바이오톡스텍 보고서 (시험번호: B02148), 2002.
39) 바이오톡스텍. 게란티 효모 G: 랫드를 이용한 13주 경구 반복투여 독성시험. 바이오톡스텍 보고서 (시험번호: B02149), 2002.
40) 바이오톡스텍. 게란티 효모 G: 세균을 이용한 복귀돌연변이시험. 바이오톡스텍 보고서 (시험번호: B02151), 2003.

41) 바이오톡스텍. 게란티 효모 G: Chinese Hamster Lung (CHL) 배양세포를 이용한 염색체이상시험. 바이오톡스텍 보고서 (시험번호: B02152), 2003.
42) 바이오톡스텍. 게란티 효모 G: 마우스를 이용한 소핵시험. 바이오톡스텍 보고서 (시험번호: B02153), 2003.
43) Sung-Hee Lee, Kyeong-Nam Oh, Sook-Nyung Rho, Bok-Hee Lee, Hyun-Joo Lee. Oral repeated-dose toxicity studies especially in the liver & kidney of rats administered with Organic Germanium-fortified Yeast., Journal Food Science and Nutrition 11:115-119, 2006.
44) Goodman S. Therapeutic effects of organic germanium. Medical hypotheses. 1988;26(3):207-15.
45) 엄상용, 장연위, 김남식, 강종원, 한윤수, 신경섭, 송형근, 박순영, 김정수, 김헌, 김용대. 금사상황버섯 추출물이 사람의 natural killer 세포 활성에 미치는 영향. 한국식품과학회지. 2006;38(5):717-19.
46) 고경봉, 김찬형, 박중규. 스트레스반응척도의 개발. 신경정신의학. 2000;39(4):707-719.

22. 연구책임자 경력사항 (별첨 1. 참고)

연구책임자 경력사항

(1) 인적사항

<table>
<tr><td>성 명</td><td colspan="7">이 종 호</td></tr>
<tr><td rowspan="5">직 장</td><td>기관명</td><td colspan="4">연세대학교</td><td>전 화</td><td>02-2123-3122</td></tr>
<tr><td>전 공</td><td colspan="4">임상영양</td><td>F A X</td><td>02-364-9605</td></tr>
<tr><td>부 서</td><td colspan="2">식품영양학과</td><td>직위</td><td>교수</td><td>휴대전화</td><td>010-5066-8795</td></tr>
<tr><td rowspan="2">주 소</td><td>우편번호</td><td colspan="3">03722</td><td rowspan="2">E-mail</td><td rowspan="2">jhleeb@yonsei.ac.kr</td></tr>
<tr><td colspan="4">서울시 서대문구 연세로 50 삼성관 525호</td></tr>
</table>

(2) 주요 경력

연 도		근무기관	직위(직명)	비고
부터	까지			
2007	2009	과학기술부 자체평가위원회	위원	
2007	현재	한국과학기술한림원	정회원	
2008	2009	한국영양학회	대의원	
2008	2012	연세대학교 노화과학연구소	소장	
2008	2014	Nutrition, Metabolism & Cardiovascular Diseases	Editorial Board Member	
2009	2010	2010년도 한국인영양섭취기준 개정위원회	검토위원(식이섬유)	
2009	2011	질병관리본부 한국인유전체역학조사사업 자문위원회 영양분과위원회	위원	
2009	2012	한국지질동맥경화학회	재무이사	
2011	2012	대한골다공증학회 영양위원회	위원	
2011	2013	한국과학기술한림원 출판홍보위원회	위원	
2011	현재	Journal of Metabolic Syndrome	Editorial Board Member	
2011	현재	World Journal of Hypertension	Editorial Board Member	
2012	현재	한국임상영양학회	학술이사	
2013	2013	한국지질동맥경화학회	회장	
2013	2015	Molecular Nutrition & Food Research	Editorial Board Member	
2013	2016	연세대학교	언더우드 특훈교수	
2014	2014	국립축산과학원 한우 분자표지 SBP 결정 사업	기술평가(심사)위원	
2014	2017	World Journal of Biological Chemistry	Editorial Board Member	
2014	현재	Journal of Biolchemistry and Molecular Biology Research	Editorial Board Member	

(3) 주요 연구실적 (지난 3년간) 및 논문 발표실적

저자명	논문명	학술지명	***IF***
Kim D-I, Yang HI, Park J-H, Lee MK, Kang D-W, Chae JS, Lee JH, Jeon JY	The Association between Resting Heart Rate and Type 2 Diabetes and Hypertension in Korean adults	Heart (Cardiac & Cardiovascular system 관련 분야 상위 11.4% 이내,	5.595

		14/123)	
Lee M-H, Kim M, Kim M, Kwak JH, Chang DH, Yu WK, Lee S-H, Lee JH	Consumption of dairy yogurt with rhamnogalacturonan in Korea citrus Hallabong peel polysaccharide enhances immune function and attenuates inflammatory response	Food & Function (Food Science & Technology 관련 분야 상위 13.8% 이내, 17/123)	2.791
Kim M, Lee S-H, Lee JH	Global metabolic profiling of plasma shows that three-year mild-caloric restriction lessens an age-related increase in sphingomyelin and reduces L-leucine and L-phenylalanine in overweight and obese subjects	Aging and Disease (Geriatrics & Gerontology 관련 분야 상위 38.0% 이내, 19/50)	3.070
Cha TW, Kim M, Kim M, Chae JS, Lee JH	Blood pressure-lowering effect of Korean red ginseng associated with decreased circulating Lp-PLA$_2$ activity and lysophosphatidylcholines and increased dihydrobiopterin level in prehypertensive subjects	Hypertension Research (Peripheral Vascular Disease 관련 분야 상위 50.0%이내, 30/60)	2.658
Lee JG, Kim YS, Lee YJ, Ahn HY, Kim M, Kim M, Cho MJ, Cho Y, Lee JH	Effect of immune-enhancing enteral nutrition enriched with or without beta-glucan on immunomodulation in critically ill patients: a randomized placebo-controlled trial	Nutrients (Nutrition & Dietetics 관련 분야 상위 27.3% 이내, 21/77)	3.270
Kim SY, Kim NK, Baik SH, Min BS, Hur H, Lee J, Noh HY, Lee JH, Koo BN	Effects of postoperative pain management on immune function after laparoscopic resection of colorectal cancer: a randomized study	Medicine (Medicine, General & Internal 관련 분야 상위 9.7% 이내, 15/154)	5.723
Kim M, Kim M, Lee YJ, Lee SP, Kim TS, Yang HJ, Kwon DY, Lee S-H, Lee JH	Effects of α-linolenic acid supplementation on collagen-epinephrine closure time, activated partial thromboplastin time and Lp-PLA$_2$ activities in nondiabetic and hypercholesterolemic subjects	Journal of Functional Foods (Food science & Technology 관련 분야 상위 5.7% 이내, 7/123)	3.574
Jun YJ, Lee J, Hwang S, Kwak JH, Ahn HY, Bak YK, Koh J, Lee JH	Beneficial effect of xylose consumption on postprandial hyperglycemia in Korean: a randomized double-blind crossover design	Trials (Medicine, Research & Experimental 관련 분야 상위 65.9% 이내, 81/123)	1.731
Kim M, Song G, Kang M, Yoo HJ, Jeong T-S, Lee S-H, Lee JH	Replacing carbohydrate with protein and fat in prediabetes or type-2 diabetes: greater effect on metabolites in PBMC than plasma	Nutrition & Metabolism (Nutrition & Dietetics 관련 분야 상위 26.6% 이내, 22/77)	3.258
Jung S, Lee YJ, Kim M, Kim M, Kwak JH, Lee J-W, Ahn Y-T, Sim J-H, Lee JH	Supplementation with two probiotic strains, *Lactobacillus curvatus* HY7601 and *Lactobacillus Plantarum* KY1032, reduced body adiposity and Lp-PLA$_2$ activity in overweight subjects	Journal of Functional Foods (Food science & Technology 관련 분야 상위 5.7%	3.574

		이내, 7/123)	
Park SE, Kim O-H, Kwak JH, Lee K-H, Kwon Y-I, Chung KH, <u>Lee JH</u>	Antihyperglycemic effect of short-term Arginyl-fructose supplementation in subjects with pre-diabetes and newly diagnosed type 2 diabetes: randomized, double-blinded, placebo-controlled trial	Trials (Medicine, Research & Experimental 관련 분야 상위 65.9% 이내, 81/123)	1.731
Jung S, Kim M, Lee YJ, Lee S-H, <u>Lee JH</u>	Associations between metabolomic-identified changes of biomarkers and arterial stiffness in subjects progressing to impaired fasting glucose: a nested case-control study	Clinical Endocrinology (Endocrinology & Metabolism 관련 분야 상위 37.5% 이내, 48/128)	3.457
Ahn HY, Kim M, Ahn Y-T, Sim J-H, Choi I-D, Lee S-H, <u>Lee JH</u>	The triglyceride-lowering effect of supplementation with dual probiotic strains, *Lactobacillus curvatus* HY7601 and *Lactobacillus plantarum* KY1032: reduction of fasting plasma lysophosphatidylcholies in nondiabetic and hypertriglyceridemic subjects	Nutrition, Metabolism & Cardiovascular Diseases (Nutrition & Dietetics 관련 분야 상위 26.0% 이내, 20/77)	3.323
Ahn HY, Kim M, Chae JS, Ahn Y-T, Sim J-H, Choi I-D Lee S-H, <u>Lee JH</u>	Supplementation with two probiotic strains, *Lactobacillus curvatus* HY7601 and *Lactobacillus plantarum* KY 1032, reduces fasting triglycerides and enhances apolipoprotein A-V levels in non-diabetic subjects with hypertriglyceridemia (nutraingredients-usa.com, 2015.07.27. 게재)	Atherosclerosis (Peripheral Vascular Disease 관련 분야 상위 18.3% 이내, 11/60)	3.994
Lee SY, Kim M, Jung S, Lee S-H, <u>Lee JH</u>	Altered plasma lysophosphatidylcholines and amides in non-obese and non-diabetic subjects with borderline-to-moderate hypertriglyceridemia	PLoS One (Multidisciplinary Sciences 관련 분야 상위 14.3% 이내, 8/56)	3.234
Paik JK, Kim M, Kim M, Yen Y, Ahn HY, Lee S-H, <u>Lee JH</u>	Circulating Lp-PLA_2 activity correlates with oxidative stress and cytokines in overweight/obese postmenopausal women not using hormone-replacement therapy	AGE: Journal of the American Aging Association (Geriatrics & Gerontology 관련 분야 상위 28.0% 이내, 14/50)	3.390
Kim M, Jung S, Lee S-H, <u>Lee JH</u>	Association between arterial stiffness and serum L-octanoylcarnitine and lactosylceramide in overweight middle-aged subjects: 3-year follow-up study	PLoS One (Multidisciplinary Sciences 관련 분야 상위 14.3% 이내, 8/56)	3.234
Kim K-C, Chun H, Lai C, Parnell LD, Jang Y, <u>Lee JH,</u> JM Ordovas	The association between genetic variants of *RUNX2*, *ADIPOQ* and vertebral fracture in Korean postmenopausal women	Journal of Bone and Mineral Metabolism (Medicine, Research & Experimental 관련 분야 상위 50.4%	2.460

		이내, 64/122)	
Jung S, Kim M, Ryu HJ, Chae JS, Lee S-H, Lee JH	Age-related increase in LDL-cholesterol is associated with enhanced oxidative stress and disturbed sphingolipid metabolism	Metabolomics (Endocrinology & Metabolism 관련 분야 상위 32.0%이내, 41/128)	3.855
Jung S, Kim M, Chae JS, Lee S-H, Joo J, Lee JH	Carriage of the V279F homozygous genotype, a rare allele, within the gene encoding *Lp-PLA*$_2$ leads to changes in circulating intermediate metabolites in individuals without metabolic syndrome	Journal of Atherosclerosis and Thrombosis (Nutrition & Dietetics 관련 분야 상위 46.7%이내, 28/60)	2.733
Kim HJ, Ahn HY, Kwak JH, Shin DY, Kwon Y-I, Oh C-G, Lee JH	The effects of Chitosan oligosaccharide (GO2KA1) supplementation on glucose control in subjects with prediabetes	Food & Function (Food science & Technology 관련 분야 상위 13.8%이내, 17/123)	2.791
Chae JS, Kwak JH, Kim M, Shin KH, Lee S-H, Jeong T-S, Lee JH	Effects of A379V variant of the *Lp-PLA*$_2$ gene on Lp-PLA$_2$ activity and markers of oxidative stress and endothelial function in Koreans	Journal of Thrombosis and Thrombolysis (Hematology 관련 분야 상위 63.2% 이내, 43/68)	2.169
Lim HH, Choi M, Kim JY, Lee JH, Kim OY	Increased risk of obesity related to total energy intake with the *APOA5*-1131T>C polymorphism in Korean premenopausal women	Nutrition Research (Nutrition & Dietetics 관련 분야 상위 48.1%이내, 33/78)	2.472
Cho JS, Baek SH, Kim JY, Lee JH, Kim OY	Serum phospholipid monounsaturated fatty acid composition and Δ-9-desaturase activity are associated with early alteration of fasting glycemic status	Nutrition Research (Nutrition & Dietetics 관련 분야 상위 48.1%이내, 33/78)	2.472
Kim M, Jeung SR, Jeong T-S, Lee S-H, Lee JH	Replacing with whole grains and legumes reduces Lp-PLA2 activities in plasma and PBMCs in patients with prediabetes or T2D	Journal of Lipid Research (Biochemistry & Molecular biology 관련 분야 상위 22.5%이내, 65/289)	4.421
Jung S, Kim OY, Kim M, Song J, Lee S-H, Lee JH	Age-related increase in alanine aminotransferase correlates with elevated levels of plasma amino	Journal of Proteome Research	4.245

	acids, decanoylcarnitine, Lp-PLA$_2$ activity, oxidative stress, and arterial stiffness	(Biochemical research methods 관련분야 상위 17.7%이내, 14/79)	
Kim YK, Kim Y, Hwang MY, Shimokawa K, Won S, Kato N, Tabara Y, Yokota M, Han B-G, Lee JH, Kim B-J	Identification of a genetic variant at 2q12.1 associated with blood pressure in East-Asians by genome-wide scan including gene-environment interactions	BMC Medical Genetics (Genetics & Heredity 관련분야 상위 65.9% 이내, 110/167)	2.083
Kim M, Jung S, Kim S, Lee S-H, Lee JH	Prehypertension associated elevation in circulating lysophosphatidylcholines, Lp-PLA$_2$ activity and oxidative stress<br>(ThermoFisher, 2014.08.20. 게재)	PLoS One (Multidisciplinary Sciences 관련분야 상위 14.3% 이내, 8/56)	3.234
Kang R, Kim M, Chae JS, Lee S-H, Lee JH	Consumption of whole grains and legumes modulates the genetic effect of the *APOA5* -1131C variant on changes in triglyceride and apolipoprotein A-V concentrations in patients with impaired fasting glucose or newly diagnosed type 2 diabetes<br>(MDLinx.com, 2014.04.23. 선정, 소개)	Trials (Medicine, Research & Experimental 관련분야 상위 65.9%이내, 81/123)	1.731
Kim M, Chae JS, Kim M, Lee S-H, Lee JH	Effects of a 3-year dietary intervention on age-related changes in triglyceride and apolipoprotein A-V levels in patients with impaired fasting glucose or new-onset type 2 diabetes as a function of the *APOA5* -1131T>C polymorphism	Nutrition Journal (Nutrition & Dietetics 관련분야 상위 46.8%이내, 36/77)	2.597
Song J, Kwon N, Lee M-H, Ko Y-G, Lee JH, Kim OY	Association of serum phospholipid PUFAs with cardiometabolic risk: Beneficial effect of DHA on the suppression of vascular proliferation/inflammation	Clinical Biochemistry (Medical laboratory technology 관련분야 상위 43.3이내, 13/30)	2.275
Lee M-H, Kwak JH, Jeon G, Lee J-W, Seo J-H, Lee H-S, Lee JH	Red ginseng relieves the effects of alcohol consumption and hangover symptoms in healthy men: a randomized crossover study	Food & Function (Food science & Technology 관련분야 상위 13.8%이내, 17/123)	2.791
Bang H, Kwak JH, Ahn HY, Shin DY, Lee JH	Korean red ginseng improves glucose control in subjects with impaired fasting glucose, impaired glucose tolerance or newly diagnosed type 2 Diabetes Mellitus	Journal of Medicinal Food (Food Science & Technology 관련분야 상위 41.5%이내, 51/123)	1.626
Kwak JH, Kim M, Lee E, Lee S-H, Ahn C-W, Lee JH	Effects of black soy peptide supplementation on blood pressure and oxidative stress: A randomized controlled trial	Hypertension Research (Peripheral	2.658

	(MDLinx.com, 2013.08.14. 선정, 소개)	Vascular Disease 관련 분야 상위 50.0%이내, 30/60)	
Paik JK, Chae JS, Kang R, Kwon N, Lee S-H, Lee JH	Effect of age on atherogenecity of LDL and inflammatory markers in healthy women	Nutrition, Metabolism & Cardiovascular Diseases (Nutrition & Dietetics 관련 분야 상위 19.2%이내, 15/78)	3.323
Kim OY, Paik JK, Lee JY, Lee S-H, Lee JH	Follow-ups of metabolic, inflammatory and oxidative stress markers and brachial-ankle pulse wave velocity in middle-aged subjects without metabolic syndrome	Clinical and Experimental Hypertension (Pharmacology & Pharmacy 관련 분야 상위 77.2%이내, 196/254)	1.234
Kim MJ, Yang HJ, Kim JH, Ahn C-W, Lee JH, Kim KS, Kwon DY	Obesity Related Metabolomic Analysis of Human Subject in the Diet Intervention Study by Ultra Performance Liquid Chromatography and Quadrupole-Time-of- Flight Mass Spectrometry	Journal of Obesity	-
Kim JY, Kim OY, Paik JK, Kwon DY, Kim HJ, Lee JH	Association of age-related changes in intermediary lipid metabolites, inflammatory and oxidative stress markers, and arterial stiffness in middle-aged men	AGE: Journal of the American Aging Association (Geriatrics & Gerontology 관련 분야 상위 28.0% 이내, 14/50)	3.390
Park S, Kim M, Paik JK, Jang Y-J, Lee S-H, Lee JH	Oxidative stress is associated with C-reactive protein in non-diabetic postmenopausal women, independent of obesity and insulin resistance (MDLinx.com, 2013.01.22. 선정, 소개)	Clinical Endocrinology (Endocrinology & Metabolism 관련 분야 상위 37.5% 이내, 48/128)	3.457
Kwak JH, Kim MS, Lee JH, Yang YJ, Lee KH, Kim OY, Lee JH	Beneficial effect of tagatose consumption on postprandial hyperglycemia in Koreans: Double-blinded crossover designed study	Food & Function (Food science & Technology 관련 분야 상위 13.8%이내, 17/123)	2.791

23. 연구비 소요내역서 (별첨 2. 참고)

연구비 소요내역서

<table>
<tr><td colspan="3">과제명</td><td colspan="3">바이오게르마늄의 면역기능증진 효과 및 면역세포 활성화 효과 평가를 위한 인체적용시험</td></tr>
<tr><td colspan="3">연구책임자(성명/소속)</td><td colspan="3">이종호 / 생활과학대학 식품영양학과</td></tr>
<tr><td colspan="3">연구비 지원기관</td><td colspan="3">게란티제약(주)</td></tr>
<tr><td colspan="3">연구기간</td><td colspan="3">IRB 승인일로부터 2019년 03월 31일까지 (18개월)</td></tr>
<tr><td colspan="3">총 연구비</td><td colspan="3"></td></tr>
<tr><td colspan="3">항목</td><td>금액(원)</td><td>백분율(%)</td><td>비고</td></tr>
<tr><td rowspan="4">연구비</td><td>인건비</td><td>연구원</td><td></td><td></td><td>.</td></tr>
<tr><td rowspan="3">직접비</td><td>기술정보 활동비</td><td></td><td></td><td>대상자 보험가입비,
대상자 교통비,
회의비 등</td></tr>
<tr><td>재료 및 전산 처리비</td><td></td><td></td><td>.</td></tr>
<tr><td>수용비 및 수수료</td><td></td><td></td><td>.</td></tr>
<tr><td colspan="3">간접비</td><td></td><td></td><td>.</td></tr>
<tr><td colspan="3">총 연구비 (합계)</td><td></td><td></td><td>※ VAT 별도</td></tr>
</table>

24. 피해자 보상에 대한 규약

피해자 보상에 대한 규약

1. 원칙

(1) 연구대상자의 신체적인 손상 (사망 포함)에 대해 보상한다.
(2) 손상의 원인이 인체적용시험용 제품 (시험용 제품 및 대조용 제품)의 섭취로 인해 발생하였을 때 연구대상자에게 보상한다.
(3) 지속적이고 불구가 될 수 있을 정도의 손상뿐만 아니라 일시적 통증 또는 쉽게 치료될 수 있는 손상에 대해서도 보상한다.
(4) 인체적용시험용 제품에 의해 생긴 이상반응 또는 발현된 이상반응의 교정처치 과정에서 발생된 손상의 경우, 인체적용시험용 제품이 직접적인 원인이 된 손상에 대하여 보상한다.

2. 다음 경우에는 보상하지 아니한다.

(1) 제공된 인체적용시험용 제품 이외의 원인으로 발생한 이상반응에 의한 손상
(2) 인체적용시험용 제품 적용중에 대한 효과 또는 혜택을 제공하지 못한데 대한 보상
(3) 서로 합의한 프로토콜에서 이탈함으로써 야기된 손상
(4) 연구대상자 또는 보호자의 부주의에 의하여 초래된 손상

3. 보상평가 기준

(1) 보상수준은 손상의 본질, 그 정도, 지속성 여부 등에 적절한 액수여야 하며, 한국의 법정에 의해 유사 손상들에 대해 일반적으로 지급되는 것과 동일해야 한다.
(2) 보상수준에 대해서 이견이 있을 경우, 양자가 수용할 수 있는 전문가의 자문을 구하여야 한다.

앞에서 언급한 여러 제반 내용을 참고하여, 연구대상자가 본 인체적용시험에 의해 어떠한 불이익이라도 받지 않도록 주의하며, 만약 본 인체적용시험에 의해 문제점이 발생할 경우 피해자 보상규약에 의거하여 책임을 질 것을 서약합니다.

2017 년 10 월 11 일

㈜게란티제약 대표이사 손 창 욱 (인)

25. 보험 증서

KB금융그룹 KB손해보험

No Fault Compensation Insurance

ORIGINAL

[1/1 페이지]

- **Policyholder** YONSEI UNIVERSITY (110-82-10500)
- **Named Insured** YONSEI UNIVERSITY (110-82-10500)
- **Policy Number** 2017-5208444 (000)
- **Policy Period** 2017-11-20 (00:00) ~ 2018-07-19 (24:00) (242 Days)
- **Covered Business/Items** No(1) 임상시험배상책임
- **Premium Basis** No(1) Persons 130 명
- **Terms & Conditions** No(1) Human Clinical Trial

1) No Fault Compensation Policy
2) Legal Liability Extension
3) A.I.D.S. and HEPATITIS Exclusion Clause
4) Extended Discovery Period Extension for 2months
5) Costs & Expenses Endorsement
6) Sanction Limitation and Exclusion Clause
7) Main contents of Clinical Trial : 바이오게르마늄의 면역기능증진 효과 및 면역세포 활성화 효과 평가를 위한 인체적용시험
8) Policy Territory : KOREA
9) Retroactive Date : 2017-11-20
10) Human Clinical Trial to Infants or younger people under 3 years : 아니오
11) Name of drug to be tested : 게르마늄
12) Diseases for whitch medicine if efficacious : 면역기능증진 및 면역세포 활성화

- **Premium** WON 2,089,000

- **L.O.L & Ded**

No(1) Human Clinical Trial

	Limit	Cur	Limit of Liability			Deductible
			any one person	any one claim	aggregate	any one claim
No Fault Compensation Policy		WON	100,000,000	100,000,000	100,000,000	1,000,000
Policy Aggregate	WON 100,000,000					

- **Premium Installments**

	Due Date	Portion	Installment Premium			Due Date	Portion	Installment Premium	
1	2017-11-20	100 %	WON	2,089,000					

- **Co-insurance [Leader : KB]**

KB												
100.00%												

IN WITNESS WHERE OF KB Insurance Co., Ltd. SEOUL, KOREA has caused this policy to be signed by its President or authorized representative, and countersigned on the Declarations page by a duly authorized representative.

KB Insurance Co.,Ltd

Yang Jong Hee
President & CEO

206492720171116145004

PDF created with pdfFactory Pro trial version www.pdffactory.com

Full Version in Korean

1. Trial Study Protocol for IRB's Review
2. **Case Report Form Designed for Clinical Trial**
3. Written Explanation to Participants
4. Consent Form for Participation
5. Consent Form for Human Biologic Materials
6. Survey for Stress Response Inventory (SRI)
7. Survey for Nutrition Intake
8. Self-Assessment Form
9. Advertisement for Recruitment

Case Report Form

바이오게르마늄의 면역기능증진 효과 및 면역세포 활성화 효과 평가를 위한 인체적용시험

CRF Version No. : 1.1

Version Date : 2017.11.13

**Sponsor**	게란티제약㈜<br>서울시 강남구 테헤란로 7길 22
연구기관	연세대학교<br>서울시 서대문구 연세로 50

스크리닝 번호	**Y** **G** (기관코드) **-S** ___ (Screening No.)
시험책임자 성명	이 종 호 교수
시험대상자 이니셜	___
무작위배정 번호	**Y** **G** (기관코드) **-R** ___ (Random No.)

연구기관	기관코드
연세대학교	**YG**

Confidential

본 인체적용시험 증례기록서에 포함되어 있는 모든 정보는 시험책임자 및 시험담당자를 위해 제공된 것으로 시험에 사용되는 식품을 섭취하는 사람에게 시험참가에 대한 서면 동의를 받기 위한 경우를 제외하고는 게란티제약(주)의 사전 서면동의 없이 제 3자에게 공개될 수 없습니다.

증례기록서 작성 지침서

1. 모든 입력사항은 검정색 볼펜을 사용하여 정자로 깨끗하고 판독이 가능하도록 명확하게 기록하여 주십시오.
2. 가능하면 약어나 머리글자 사용을 삼가하여 주십시오. 만일 약어 사용시, 의학적으로 분명하고 표준화된 약어만 사용하여 주십시오.
3. 증례기록서의 각 페이지 상단의 제공된 공간에 대상자 번호와 이니셜을 기록해 주십시오.
4. 증례기록서에 지시된 단위 내에서 모든 수치를 기록해 주십시오.
5. 빈칸이 없도록 모든 항목을 기입해 주십시오. 빈칸은 최대 길이의 답에 알맞게 주어집니다. 숫자로 답해야 하는 경우 그 수치가 한자리라면, 예를 들어 3일 경우, 다음과 같이 기록되어야 합니다.

   예) 생년월일이 1968년 3월 10일이라면, 다음과 같이 기록되어야 합니다.

1	9	6	8	0	3	1	0
년 YYYY				월 MM		일 DD	

6. 누락된 정보에는 다음의 약어를 사용하여 주십시오.
   - ND(Not Done) = 시행하지 않음
   - NA(Not Available/ Not Applicable) = 유효하지 않음/ 적용되지 않음
   - UK(Unknown) = 알지 못함

   예) 병력 부분의 특정 항목에서 날짜가 정확하지 않을 경우 다음과 같이 기록되어야 합니다.

1	9	6	8	0	8	U	K
년 YYYY				월 MM		일 DD	

7. 기재사항을 수정할 경우에는 한 줄을 그은 후, 틀린 입력 자료 바로 옆에 수정할 자료, 날짜 그리고 수정한 사람의 서명을 기록하여 주십시오. 수정액 또는 수정 테이프는 사용하지 않아야 합니다.

   예) **X Y Z**
   ~~A B C~~ 2017.10.10 *서명*

   기록되었던 이전 정보를 지우지 마십시오. 이전 기록은 읽을 수 있어야 합니다.

8. 문구나 문장이 필요한 경우에는 철자가 분명하게 또박또박 기록하여 주십시오. 특히 이상반응을 기술하는 경우 등에 있어서 잘 알려진 의학용어를 사용하여야 합니다.
9. 약물명은 성분명으로 정확히 기록하여 주십시오. 널리 사용되고 있는 복합제의 경우에는 제품명을 기록할 수 있습니다.
10. 대상자가 연구가 중단되거나 종료된 경우 종결 Sheet 항목에 대한 기록이 완료되어야 합니다.
11. 임상병리검사의 검사일자는 채혈한 일자로 기록해 주시기 바랍니다.
12. 소실되지 않은 AE의 연구 중단일/종료일은 AE 소실을 확인하여 알게 된 날짜입니다.

◎ 인체적용시험 진행 일정표

Period		Screening	Active Treatment		
Visit		1	2	3	4
Week		-2	0	4	8
Window period (day)				±7	±5
서면동의서		✓			
인구학적 조사		✓			
병력/약물 투여력 조사		✓	✓		
활력징후(혈압, 맥박) 측정		✓		✓	✓
체온측정		✓	✓	✓	✓
이학적 검사		✓		✓	✓
임상병리검사		✓			✓
임신반응검사		✓			✓
식이조사			✓	✓	✓
스트레스 반응척도(SRI)			✓		
상기도 감염 발생여부 조사		✓			
면역 관련 유전자 검사			✓		
신체계측	신장	✓			
	체중	✓		✓	✓
유효성 평가	NK cell activity		✓	✓	✓
	WBC 수	✓		✓	✓
	IFN- γ		✓	✓	✓
	IL-2, 6, 12		✓	✓	✓
	TNF-α		✓	✓	✓
	IgG1, IgG2, IgM		✓	✓	✓
	시험대상자 자신에 의한 개선도 평가			✓	✓
시험대상자 적합성 평가		✓	✓		
무작위배정			✓		
시험식품 및 대조식품 처방			✓	✓	
병용약물 및 병용요법 확인				✓	✓
이상반응 확인				✓	✓
순응도 확인				✓	✓

방문 1
(Screening Visit, Week-2)

Protocol No.: GRT_Bio_Ge

Screening No. Y G -S

방문일

방문일을 기록하여 주십시오. .... 2 0 | 년 YYYY | 월 MM | 일 DD

서면동의서

시험대상자에게 본 인체적용시험에 관하여 자세히 설명하고 서면동의서를 받았습니까?

☐ 예 .... 2 0 | 년 YYYY | 월 MM | 일 DD

☐ 아니오 ☞ 시험에서 제외

* 인체적용시험 시작을 위해 가장 먼저 수행되어야 하며, 동의 일자는 동의서 원본과 동일하여야 합니다.

* 시험대상자가 서명한 동의서의 사본을 대상자에게 배부하여 주십시오.

인구학적 조사

시험대상자 이니셜 : ....

* (예: 홍길동→ H K D)

성별 : .... ☐ 남 ☐ 여

생 년 월 일 : .... 년 YYYY | 월 MM | 일 DD

* 주민등록번호 앞의 6자리와 동일하여야 합니다.

연 령 : .... 만 세

* (동의년도) - (출생년도) = ○○세 (동의일 기준 생일이 지나지 않은 경우 –1세)

* 만 25세 이상 만 75세 이하인 사람만 시험 참여 가능

인구학적 조사

면역질환 관련 증상 가족력 ☐ 1. 있음 ☐ 2. 없음

흡연여부
- ☐ 1. 아니오 (Non-Smoker)
- ☐ 2. Ex-Smoker (6개월 이상 금연)
- ☐ 3. Smoker (흡연량 : _____개비/1일), (흡연기간 : _____년)

음주여부
- ☐ 1. 아니오
- ☐ 2. 끊었다
- ☐ 3. 1병 미만/일주일
- ☐ 4. 1병~3병/일주일
- ☐ 5. 4병 이상/일주일

운동여부
- ☐ 1. 아니오
- ☐ 2. 3회 미만/주 (운동량:______분/1회)
- ☐ 3. 3회 이상/주 (운동량:______분/1회)

약물투여력 조사

스크리닝 방문 기준 4주 이내에 투여한 약물이 있습니까?

☐ 예 ☞ 약물투여력 조사 페이지(p.27)를 작성하여 주십시오.

☐ 아니오

방문 1
(Screening Visit, Week-2)

Protocol No.: GRT_Bio_Ge

Screening No. Y G -S

동반질환 및 병력 조사

☐ 없음

☞ 스크리닝 3개월 전부터 현재까지 동반질환 및 병력이 있는 경우 분류번호를 참고하여 기술하십시오.

No.	분류 번호*	진단명	과거	현재	진단일 (년YYYY / 월MM)	임상적 의미	등록 가능#)
1			☐	☐	/	☐있음 ☐없음	☐예 ☐아니오
2			☐	☐	/	☐있음 ☐없음	☐예 ☐아니오
3			☐	☐	/	☐있음 ☐없음	☐예 ☐아니오
4			☐	☐	/	☐있음 ☐없음	☐예 ☐아니오
5			☐	☐	/	☐있음 ☐없음	☐예 ☐아니오

* 다음에 해당되는 자는 본 인체적용시험에 참여하실 수 없습니다.
- 임상적으로 유의한 급성 또는 만성 심뇌혈관계, 면역계, 호흡기계, 간담도계, 신장 및 비뇨기계, 신경계, 근골격계질환, 정신성, 감염성 및 혈액 종양성 질환 등으로 현재 치료 중이거나 과거력이 있는 자
  (단, 대상자의 상태를 고려하여 시험책임자의 판단에 따라 연구에 참여 가능하다)
- 속쓰림, 소화불량 등의 심한 위장관 증상을 호소하는 자
- 기타 사유로 시험자가 본 연구에 부적절하다고 판단되는 사람

임상적 의미 "있음"으로 표시된 경우에는 본 인체적용시험에 "등록 가능" 여부를 반드시 체크해 주십시오.

수술력 조사

☐ 없음

☞ 스크리닝 3개월 전부터 현재까지 수술력이 있는 경우 분류번호를 참고하여 기술하십시오.

No.	분류 번호*	수술명	수술일 (년YYYY / 월MM)	퇴원일 (년YYYY / 월MM)	임상적 의미	등록 가능#
1			/	/	☐있음 ☐없음	☐예 ☐아니오
2			/	/	☐있음 ☐없음	☐예 ☐아니오
3			/	/	☐있음 ☐없음	☐예 ☐아니오

임상적 의미 "있음"으로 표시된 경우에는 본 인체적용시험에 "등록 가능" 여부를 반드시 체크해 주십시오.

*분류번호

01	Blood and lymphatic system disorders	02	Cardiac disorders	03	Congenital, familial and genetic disorders
04	Ear and labyrinth disorders	05	Endocrine disorders	06	Eye disorders
07	Gastrointestinal disorders	08	General disorders and administration site conditions	09	Hepatobiliary disorders
10	Immune system disorders	11	Infections and infestations	12	Injury, poisoning and procedural complications
13	Investigations	14	Metabolism and nutrition disorders	15	Musculoskeletal and connective tissue disorders
16	Neoplasms benign, malignant and unspecified (incl cysts and polyps)	17	Nervous system disorders	18	Pregnancy, puerperium and perinatal conditions
19	Product issues	20	Psychiatric disorders	21	Renal and urinary disorders
22	Reproductive system and breast disorders	23	Respiratory, thoracic and mediastinal disorders	24	Skin and subcutaneous tissue disorders
25	Social circumstances	26	Surgical and medical procedures	27	Vascular disorders

☐ 추가 페이지 있음

방문 1
(Screening Visit, Week-2)

Protocol No.: GRT_Bio_Ge

Screening No. Y G -S

활력징후	☐ 미실시 (사유: ________________________)
검사일:	☐ 방문일과 동일<br>☐ 방문일과 상이할 경우 체크 후, 우측에 날짜 기재 년 YYYY 월 MM 일 DD

혈압 (mmHg)	Systolic / Diastolic	맥박 (회/min)	

*조절되지 않는 고혈압 환자(140/90mmHg 이상, 10분 안정 후 측정기준)는 본 인체적용시험에 참여할수 없습니다.

체온 측정	☐ 미실시 (사유: ________________________)
검사일:	☐ 방문일과 동일<br>☐ 방문일과 상이할 경우 체크 후, 우측에 날짜 기재 년 YYYY 월 MM 일 DD
체온 (℃)	.

신체계측	☐ 미실시 (사유: ________________________)
검사일:	☐ 방문일과 동일<br>☐ 방문일과 상이할 경우 체크 후, 우측에 날짜 기재 년 YYYY 월 MM 일 DD
신장 (cm)	.
체중 (kg)	.

☞ 신장은 0.1 cm, 체중은 0.1 kg 단위까지 반올림하여 기록해 주십시오.

방문 1
(Screening Visit, Week-2)

Protocol No.: GRT_Bio_Ge

Screening No. Y G -S

유효성 검사	☐ 미실시 (사유:)
검사일: ☐ 방문일과 동일<br>☐ 방문일과 상이할 경우 체크 후, 우측에 날짜 기재 (년 YYYY 월 MM 일 DD)	
항목	스크리닝 결과
**WBC 수 (cell/ul)**	**X 10^3**
* 혈액검사에서 말초혈액 백혈구 수치가 $4X10^3$ cells/ul 이상 ~ $8X10^3$ cells/ul 미만인 사람(대상자 스크리닝 당시 기준)이 본 연구에 참여할 수 있습니다.<br>시험대상자 선정기준 중 백혈구 수에 대한 평가 결과, 이 지원자는 시험에 참여하기 적합합니까?<br>☐ 예 ☐ 아니오	

상기도 감염 발생여부 조사	☐ 미실시 (사유:)
스크리닝 기준 1년 이내에 상기도감염이 2회 이상 발생하였는가?<br>☐ 예 (☐편도염 ☐인두염 ☐후두염 ☐부비강염 ☐중이염 ☐비염 ☐기타( ))<br>☐ 아니오	

이학적 검사	☐ 미실시 (사유 : ______)	
신체기관명	정상여부	비정상인 경우 의학적 의견 기술
심혈관계	☐ 정상 ☐ 비정상	1. ______<br>2. ______
폐 및 호흡기계	☐ 정상 ☐ 비정상	1. ______<br>2. ______
위장관/간 및 담도계	☐ 정상 ☐ 비정상	1. ______<br>2. ______
대사/내분비계	☐ 정상 ☐ 비정상	1. ______<br>2. ______
신장/요로계	☐ 정상 ☐ 비정상	1. ______<br>2. ______
생식기계	☐ 정상 ☐ 비정상	1. ______<br>2. ______
근골격계	☐ 정상 ☐ 비정상	1. ______<br>2. ______
피부 및 결합조직	☐ 정상 ☐ 비정상	1. ______<br>2. ______
신경계	☐ 정상 ☐ 비정상	1. ______<br>2. ______
정신계	☐ 정상 ☐ 비정상	1. ______<br>2. ______
기타	☐ 정상 ☐ 비정상	1. ______<br>2. ______

방문 1
(Screening Visit, Week-2)

Protocol No.: GRT_Bio_Ge

Screening No. Y G -S

임상병리검사

☐ 미실시 (사유: ______________________)

항목			검사치	재검치[1)]	정상		임상적 의미		소견
검사일자(YYYY/MM/DD)			/ /	/ /	예	아니오[2)]	예[3)]	아니오	
혈액학적검사	WBC	x10³/uL			☐	☐	☐	☐	
	RBC	x10³/uL			☐	☐	☐	☐	
	Hb	g/dL			☐	☐	☐	☐	
	Hct	%			☐	☐	☐	☐	
	Platelet	$10^3/mm^3$			☐	☐	☐	☐	
	Lymphocyte	$10^3/mm^3$			☐	☐	☐	☐	
	Monocyte	$10^3/mm^3$			☐	☐	☐	☐	
혈액화학적검사	AST(GOT)[4)]	U/L			☐	☐	☐	☐	
	ALT(GPT)[4)]	U/L			☐	☐	☐	☐	
	Total Protein	g/dL			☐	☐	☐	☐	
	Glucose[5)]	mg/dL			☐	☐	☐	☐	
	Total cholesterol	mg/dL			☐	☐	☐	☐	
	BUN	mg/dL			☐	☐	☐	☐	
	Creatinine[6)]	mg/dL			☐	☐	☐	☐	
	Uric acid	mg/dL			☐	☐	☐	☐	
	Ca	mg/dL			☐	☐	☐	☐	
	P	mg/dL			☐	☐	☐	☐	
소변검사	SG	-			☐	☐	☐	☐	
	pH	-			☐	☐	☐	☐	
	Protein	-			☐	☐	☐	☐	
	Glucose	-			☐	☐	☐	☐	
	Ketone	-			☐	☐	☐	☐	
	Bilirubin	-			☐	☐	☐	☐	
	Urobilinogen	-			☐	☐	☐	☐	
	Nitrite	-			☐	☐	☐	☐	
	WBC(Leukocyte)	-			☐	☐	☐	☐	
	Erythrocyte	-			☐	☐	☐	☐	

방문 1
(Screening Visit, Week-2)

Protocol No.: GRT_Bio_Ge

Screening No. Y G -S

1) 재검치가 기록된 경우 재검치에 의해 정상여부를 표기하고 임상적 의미를 판단하여 표기하십시오.
2) 정상여부 '아니오'인 경우 임상적 의미 여부에 표기하십시오.
3) 임상적의미 '예'인 경우 소견란에 기재하십시오.
4) AST 또는 ALT 혈중치가 120 IU/L 초과한 자는 본 인체적용시험에 참여할 수 없습니다
5) 공복 혈당이 126 mg/dl 이상인 자는 본 인체적용시험에 참여할 수 없습니다.
6) Creatinine 혈중치가 남자 2.4 mg/dL, 여자 1.8 mg/dL 초과한 자는 본 인체적용시험에 참여하실 수 없습니다.
* 누락된 항목의 경우 검사결과란에 ND(Not Done)로 적고, 그 사유를 소견란에 기재하십시오.
** 임상적 의미가 있어 새롭게 진단받은 질환이 있는 경우 동반질환 및 병력 페이지(p.6)에 기록하여 주십시오.

임신반응검사*

* 임신가능성이 있는 가임기 여성의 경우에 한하여 실시합니다.

☐ 미실시 ☞ 사유 : ☐ 불임수술 ☐ 폐경 ☐ 남성 ☐ 기타 (____________________)

검사일: ☐ 방문일과 동일

☐ 방문일과 상이할 경우 체크 후, 우측에 날짜 기재 [| | |] 년 YYYY [|] 월 MM [|] 일 DD

검사결과 ……… ☐ 음성 ☐ 양성 ☞ "양성"인 경우 본 인체적용시험에 참여할 수 없습니다.

선정/제외기준 확인

선정/제외기준을 확인한 결과, 본 시험대상자가 인체적용시험에 참여하기에 적합합니까?

☐ 예

☐ 아니오 ☞ 사유 : ☐ 선정/제외기준 위반 ☞ ☐ 선정(No.) /☐ 제외(No.)

☐ 동의철회

☐ 기타

☞ 시험종결 페이지(p.29)를 작성하여 주십시오.

인체적용시험을 지속한다면, 다음 방문일(14일 이내)을 지정하여 주십시오.

방문 2
(Baseline Visit, Week 0)

Protocol No.: GRT_Bio_Ge

Screening No. Y G - S _ _ _

방문일

방문일을 기록하여 주십시오 .... 2 0 _ _ / _ _ / _ _
년 YYYY 월 MM 일 DD

동반질환, 병력, 수술력 변화 확인

방문1 이후 새로운 의학적인 문제 또는 질병이 발생하였습니까?

☐ 예 ☞ 동반질환 / 기타 병력 및 수술력 조사 페이지(p.6)를 작성하여 주십시오.

☐ 아니오

약물투여력 변화

방문1 이후 새로운 약물을 투여, 복용하였습니까?

☐ 예 ☞ 약물 투여력 조사 페이지(p. 27)를 작성하여 주십시오.

☐ 아니오

체온 측정

☐ 미실시 (사유: ____________________)

검사일: ☐ 방문일과 동일

☐ 방문일과 상이할 경우 체크 후, 우측에 날짜 기재 _ _ _ _ / _ _ / _ _
년 YYYY 월 MM 일 DD

체온 (℃)	_ _ . _

스트레스 반응척도(SRI)

☐ 미실시 (사유:)

검사일 ☐ 방문일과 동일

☐ 방문일과 상이할 경우 체크 후, 우측에 날짜 기재 _ _ _ _ / _ _ / _ _
년 YYYY 월 MM 일 DD

점수	/ 156

방문 2
(Baseline Visit, Week 0)

Protocol No.: GRT_Bio_Ge

Screening No. Y G - S

유효성 검사

☐ 미실시 (사유:)

검사일 ☐ 방문일과 동일

☐ 방문일과 상이할 경우 체크 후, 우측에 날짜 기재 년 YYYY 월 MM 일 DD

항목	결과	항목	결과
**NK cell activity (%, 2.5:1)**		**IgM(mg/dL)**	
**NK cell activity (%, 5:1)**		**IL-2 (pg/ml)**	
**NK cell activity (%, 10:1)**		**IL-6 (pg/ml)**	
**NK cell activity (%, 50:1)**		**IL-12 (pg/ml)**	
**IgG1(mg/dL)**		**IFN-γ (pg/ml)**	
**IgG2(mg/dL)**		**TNF-α (pg/ml)**	

면역 관련 유전자 검사

면역 등과 관련한 유전자 검사를 시행 하였습니까?

□ 예 □ 아니오 (사유: ____)

식이조사

24시간 회상법 작성을 확인하고, 식이조사지를 배부하였습니까?

□ 예 □ 아니오 (사유: ____)

시험대상자 적합성 평가

☐ 미실시 (사유: ____)

방문 2
(Baseline Visit, Week 0)

Protocol No.: GRT_Bio_Ge

Screening No. Y G - S

선정기준 (Inclusion Criteria)	
1. 만 25세 이상 ~ 만 75세 이하의 남, 여	☐ 예 ☐ 아니오
2. 스크리닝 검사에서 측정한 말초혈액 백혈구 수치가 $4X10^3$ cells/ul 이상 ~ $8X10^3$ cells/ul 미만인 자	☐ 예 ☐ 아니오
3. 본 연구에 참여할 것을 동의하고 서면 동의서에 연구대상자(또는 법정 대리인)가 자의로 서명한 자	☐ 예 ☐ 아니오
* 상기 사항이 모두 "예"이면 이 지원자는 인체적용시험에 참여할 수 있습니다.	

제외기준 (Exclusion Criteria)	
1. 임상적으로 유의한 급성 또는 만성 심뇌혈관계, 면역계, 호흡기계, 간담도계, 신장 및 비뇨기계, 신경계, 근골격계 질환, 정신성, 감염성 및 혈액·종양성 질환 등으로 현재 치료 중인 자<br>(단, 대상자의 상태를 고려하여 시험책임자의 판단에 따라 연구에 참여 가능하다)	☐ 예 ☐ 아니오
2. 조절되지 않는 고혈압이 있는 자 (140/90mmHg 이상, 시험대상자 10분 안정 후 측정기준)	☐ 예 ☐ 아니오
3. 조절되지 않는 당뇨병 환자 (공복혈당 126mg/dl 이상 또는 3개월 이내에 당뇨로 인해 약제를 새로 시작하는 경우)	☐ 예 ☐ 아니오
4. 스크리닝 전 3개월 이내에 예방접종을 실시한 자	☐ 예 ☐ 아니오
5. AST(GOT) 또는 ALT(GPT) 혈중치가 120 IU/L 초과한 자	☐ 예 ☐ 아니오
6. Creatinine 혈중치가 남자 2.4 mg/dL, 여자 1.8 mg/dL 초과한 자	☐ 예 ☐ 아니오
7. 스크리닝 전 2주 이내에 면역에 영향을 미칠 수 있는 건강기능식품을 섭취했거나 섭취중인 자	☐ 예 ☐ 아니오
8. 속쓰림, 소화불량 등의 심한 위장관 증상을 호소하는 자	☐ 예 ☐ 아니오
9. 임신, 수유 중이거나 본 연구 기간 동안 임신 계획이 있는 자	☐ 예 ☐ 아니오
10. 시험 식품 관련 식품에 민감하거나 알레르기가 있는 자	☐ 예 ☐ 아니오
11. 본 연구 중에 다른 연구에 참가할 계획이 있는 자	☐ 예 ☐ 아니오
12. 본 연구 시작 4주 이내에 다른 인체적용시험에 참여했던 자	☐ 예 ☐ 아니오
13. 연구자가 본 인체적용시험에 부적합하다고 판단하는 자	☐ 예 ☐ 아니오
* 상기 사항이 모두 "아니오"이면 이 지원자는 인체적용시험에 참여할 수 있습니다.	

시험대상자 선정기준 및 제외기준에 대한 평가 결과

이 지원자는 본 인체적용시험에 참여하기 적합합니까?

☐ 예 ☐ 아니오

방문 2
(Baseline Visit, Week 0)

Protocol No.: GRT_Bio_Ge

Screening No. Y G - S

평가일자 : 2 0 년 YYYY 월 MM 일 DD

연구자 서명 :

무작위배정 및 시험/대조식품 처방 (1차)		
배정번호	Y G (기관코드)	- R (Random No.)

인체적용시험을 지속한다면, 방문2를 기준으로 다음 방문일(28일±7일 이내)을 지정하여 주십시오.
해당 번호의 시험식품/대조식품(4주 + 여유분)을 처방하였습니까?

☐ 예 ☞ 시험/대조식품을 처방일부터 섭취하도록 교육하십시오.

☐ 아니오 ☞ 사유 : ☐ 중도탈락

☐ 기타 (______________________________)

☞ 시험종결 페이지(p.29)를 작성하여 주십시오.

방문 3
(Interim Visit, Week 4)

Protocol No.: GRT_Bio_Ge

Screening No. Y G - S

방문일

방문일을 기록하여 주십시오 .... 2 0 __ __ 년 YYYY __ 월 MM __ 일 DD

이상반응 확인

방문2 이후 새로운 의학적인 문제 또는 질병이 발생하였습니까?

☐ 예 ☞ 이상반응 페이지(p.28)를 작성하여 주십시오.

☐ 아니오

병용약물 및 병용요법 확인

방문2 이후 새로운 약물을 투여, 복용하였습니까?

☐ 예 ☞ 병용약물 및 병용요법 페이지(p.27)을 작성하여 주십시오.

☐ 아니오

활력징후

☐ 미실시 (사유: ______________________)

검사일: ☐ 방문일과 동일

☐ 방문일과 상이할 경우 체크 후, 우측에 날짜 기재 __ __ __ __ 년 YYYY __ 월 MM __ 일 DD

혈압 (mmHg)	Systolic / Diastolic	맥박 (회/min)	

방문 3
(Interim Visit, Week 4)

Protocol No.: GRT_Bio_Ge

Screening No. Y G - S

체온 측정

☐ 미실시 (사유: ________________)

검사일:	☐ 방문일과 동일<br>☐ 방문일과 상이할 경우 체크 후, 우측에 날짜 기재 년 YYYY 월 MM 일 DD
체온 (℃)	.

신체계측

☐ 미실시 (사유: ________________)

검사일:	☐ 방문일과 동일<br>☐ 방문일과 상이할 경우 체크 후, 우측에 날짜 기재 년 YYYY 월 MM 일 DD
체중 (kg)	.

☞ 체중은 0.1 kg 단위까지 반올림하여 기록해 주십시오.

이학적 검사

☐ 미실시 (사유 : ________________)

신체기관명	정상여부	비정상인 경우 의학적 의견 기술
심혈관계	☐ 정상 ☐ 비정상	1. ______<br>2. ______
폐 및 호흡기계	☐ 정상 ☐ 비정상	1. ______<br>2. ______
위장관/간 및 담도계	☐ 정상 ☐ 비정상	1. ______<br>2. ______
대사/내분비계	☐ 정상 ☐ 비정상	1. ______<br>2. ______
신장/요로계	☐ 정상 ☐ 비정상	1. ______<br>2. ______
생식기계	☐ 정상 ☐ 비정상	1. ______<br>2. ______

방문 3
(Interim Visit, Week 4)

Protocol No.: GRT_Bio_Ge

Screening No. Y G - S

근골격계	☐ 정상 ☐ 비정상	1. ____<br>2. ____
피부 및 결합조직	☐ 정상 ☐ 비정상	1. ____<br>2. ____
신경계	☐ 정상 ☐ 비정상	1. ____<br>2. ____
정신계	☐ 정상 ☐ 비정상	1. ____<br>2. ____
기타	☐ 정상 ☐ 비정상	1. ____<br>2. ____

* 새로운 증상이 나타나거나 질환이 악화된 경우 이상반응 페이지(p.28)를 작성하여 주십시오.

유효성 검사

☐ 미실시 (사유:)

검사일 ☐ 방문일과 동일

☐ 방문일과 상이할 경우 체크 후, 우측에 날짜 기재 년 YYYY 월 MM 일 DD

항목	결과	항목	결과
**NK cell activity (%, 2.5:1)**		**IgG1(mg/dL)**	
**NK cell activity (%, 5:1)**		**IgG2(mg/dL)**	
**NK cell activity (%, 10:1)**		**IgM(mg/dL)**	
**NK cell activity (%, 50:1)**		**IFN-γ (pg/ml)**	
**IL-2 (pg/ml)**		**TNF-α (pg/ml)**	
**IL-6 (pg/ml)**		**WBC 수(x10^3 cells/uL)**	

방문 3
(Interim Visit, Week 4)

Protocol No.: GRT_Bio_Ge

Screening No. Y G - S

**IL-12 (pg/ml)**			

시험대상자 자신에 의한 개선도 평가		☐ 미실시 (사유:)		
검사일 ☐ 방문일과 동일<br>☐ 방문일과 상이할 경우 체크 후, 우측에 날짜 기재			년 YYYY 월 MM 일 DD	
**1.** 매우 우수	**2.** 우수	**3.** 불변	**4.** 악화	**5.** 매우악화

식이조사
식이조사지 작성을 확인하고 식이조사지를 배부하였습니까?<br>☐ 예 ☐ 아니오 (사유: ____)

순응도 확인

배부된 1차 시험/대조 식품이 반납되었습니까? ☐ 예 ☐ 아니오(사유:)

섭취기간 기준은 '인체적용시험용 식품 처방날 ~ 방문3 전날' 입니다.

처방 식품 수	**4**주분 **+** 여유분	비 고
섭취해야 할 식품 수 [a]	____________________ 캡슐	
섭취한 식품 수 [b]	____________________ 캡슐	
남은 식품 수	____________________ 캡슐	
분실된 식품 수	____________________ 캡슐	
섭취율**(%) (= b/a *100)**	____________________ %	반올림하여 소수점 1자리까지 표기

방문 3
(Interim Visit, Week 4)

Protocol No.: GRT_Bio_Ge

Screening No. Y G - S

시험/대조식품 처방 (2차)
해당 시험대상자에게 배정된 시험식품/대조식품(4주 + 여유분)을 처방하였습니까?

☐ 예 ☞ 시험/대조식품을 처방일부터 섭취하도록 교육하십시오.

☐ 아니오 ☞ 사유 : ☐ 중도탈락

☐ 기타 (______________________________)

☞ 시험종결 페이지(p.29)를 작성하여 주십시오.

인체적용시험을 지속한다면, 방문 2를 기준으로 다음 방문일(56일±5일 이내)을 지정하여 주십시오.

방문 4
(Closing Visit, Week 8)

Protocol No.: GRT_Bio_Ge

Screening No. Y G - S

방문일

방문일을 기록하여 주십시오 .... 2 0 _ _ 년 YYYY _ _ 월 MM _ _ 일 DD

이상반응 확인

방문3 이후 새로운 의학적인 문제 또는 질병이 발생하였습니까?

☐ 예 ☞ 이상반응 페이지(p.28)를 작성하여 주십시오.

☐ 아니오

병용약물 및 병용요법 확인

방문3 이후 새로운 약물을 투여, 복용하였습니까?

☐ 예 ☞ 병용약물 및 병용요법 페이지(p.27)을 작성하여 주십시오.

☐ 아니오

활력징후

☐ 미실시 (사유: ______________________________)

검사일: ☐ 방문일과 동일

☐ 방문일과 상이할 경우 체크 후, 우측에 날짜 기재 _ _ _ _ 년 YYYY _ _ 월 MM _ _ 일 DD

혈압 (mmHg)	_ _ _ Systolic / _ _ _ Diastolic	맥박 (회/min)	_ _ _

Protocol No.: GRT_Bio_Ge

방문 4
(Closing Visit, Week 8)

Screening No. Y G - S

신체계측	☐ 미실시 (사유: ______________)
검사일:	☐ 방문일과 동일<br>☐ 방문일과 상이할 경우 체크 후, 우측에 날짜 기재 ____년 YYYY __월 MM __일 DD
체중 (kg)	___ . _
☞ 체중은 0.1 kg 단위까지 반올림하여 기록해 주십시오.	

체온측정	☐ 미실시 (사유: ______________)
검사일:	☐ 방문일과 동일<br>☐ 방문일과 상이할 경우 체크 후, 우측에 날짜 기재 ____년 YYYY __월 MM __일 DD
체온 (℃)	__ . _

이학적 검사 ☐ 미실시 (사유 : ______________)

신체기관명	정상여부	비정상인 경우 의학적 의견 기술
심혈관계	☐ 정상 ☐ 비정상	1. ______<br>2. ______
폐 및 호흡기계	☐ 정상 ☐ 비정상	1. ______<br>2. ______
위장관/간 및 담도계	☐ 정상 ☐ 비정상	1. ______<br>2. ______
대사/내분비계	☐ 정상 ☐ 비정상	1. ______<br>2. ______
신장/요로계	☐ 정상 ☐ 비정상	1. ______<br>2. ______

Protocol No.: GRT_Bio_Ge

방문 4
(Closing Visit, Week 8)

Screening No.
Y G - S

생식기계	☐ 정상 ☐ 비정상	1. ______<br>2. ______
근골격계	☐ 정상 ☐ 비정상	1. ______<br>2. ______
피부 및 결합조직	☐ 정상 ☐ 비정상	1. ______<br>2. ______
신경계	☐ 정상 ☐ 비정상	1. ______<br>2. ______
정신계	☐ 정상 ☐ 비정상	1. ______<br>2. ______
기타	☐ 정상 ☐ 비정상	1. ______<br>2. ______
* 새로운 증상이 나타나거나 질환이 악화된 경우 이상반응 페이지(p.29)를 작성하여 주십시오.		

방문 4
(Closing Visit, Week 8)

Protocol No.: GRT_Bio_Ge

Screening No. Y G - S

임상병리검사	☐ 미실시 (사유: ______________________)

항목			검사치	재검치[1)]	정상		임상적 의미		소견
검사일자(YYYY/MM/DD)			/ /	/ /	예	아니오[2)]	예[3)]	아니오	
혈액학적검사	WBC	$x10^3$/uL			☐	☐	☐	☐	
	RBC	$x10^3$/uL			☐	☐	☐	☐	
	Hb	g/dL			☐	☐	☐	☐	
	Hct	%			☐	☐	☐	☐	
	Platelet	10^3/mm^3			☐	☐	☐	☐	
	Lymphocyte	10^3/mm^3			☐	☐	☐	☐	
	Monocyte	10^3/mm^3			☐	☐	☐	☐	
혈액화학적검사	AST(GOT)	U/L			☐	☐	☐	☐	
	ALT(GPT)	U/L			☐	☐	☐	☐	
	Total Protein	g/dL			☐	☐	☐	☐	
	Glucose	mg/dL			☐	☐	☐	☐	
	Total cholesterol	mg/dL			☐	☐	☐	☐	
	BUN	mg/dL			☐	☐	☐	☐	
	Creatinine[6)]	mg/dL			☐	☐	☐	☐	
	Uric acid	mg/dL			☐	☐	☐	☐	
	Ca	mg/dL			☐	☐	☐	☐	
	P	mg/dL			☐	☐	☐	☐	
소변검사	SG	-			☐	☐	☐	☐	
	pH	-			☐	☐	☐	☐	
	Protein	-			☐	☐	☐	☐	
	Glucose	-			☐	☐	☐	☐	
	Ketone	-			☐	☐	☐	☐	
	Bilirubin	-			☐	☐	☐	☐	
	Urobilinogen	-			☐	☐	☐	☐	
	Nitrite	-			☐	☐	☐	☐	
	WBC(Leukocyte)	-			☐	☐	☐	☐	
	Erythrocyte	-			☐	☐	☐	☐	

Protocol No.: GRT_Bio_Ge

방문 4
(Closing Visit, Week 8)

Screening No.
Y G - S

1) 재검치가 기록된 경우 재검치에 의해 정상여부를 표기하고 임상적 의미를 판단하여 표기하십시오.
2) 정상여부 '아니오'인 경우 임상적 의미 여부에 표기하십시오.
3) 임상적의미 '예'인 경우 소견란에 기재하십시오
* 누락된 항목의 경우 검사결과란에 ND(Not Done)로 적고, 그 사유를 소견란에 기재하십시오.

임신반응검사*

* 임신가능성이 있는 가임기 여성의 경우에 한하여 실시합니다.

☐ 미실시 ☞ 사유 : ☐ 불임수술 ☐ 폐경 ☐ 남성 ☐ 기타 (____________________)

검사일: ☐ 방문일과 동일

☐ 방문일과 상이할 경우 체크 후, 우측에 날짜 기재 년 YYYY 월 MM 일 DD

검사결과 .... ☐ 음성 ☐ 양성

유효성 검사

☐ 미실시 (사유:)

검사일 ☐ 방문일과 동일

☐ 방문일과 상이할 경우 체크 후, 우측에 날짜 기재 년 YYYY 월 MM 일 DD

항목	결과	항목	결과
**NK cell activity (%, 2.5:1)**		**IgG1(mg/dL)**	
**NK cell activity (%, 5:1)**		**IgG2(mg/dL)**	
**NK cell activity (%, 10:1)**		**IgM(mg/dL)**	
**NK cell activity (%, 50:1)**		**IFN-γ (pg/ml)**	
**IL-2 (pg/ml)**		**TNF-α (pg/ml)**	
**IL-6 (pg/ml)**		**WBC 수(x10^3 cells/uL)**	
**IL-12 (pg/ml)**			

방문 4
(Closing Visit, Week 8)

Protocol No.: GRT_Bio_Ge

Screening No. Y G - S

시험대상자 자신에 의한 개선도 평가

☐ 미실시 (사유:)

검사일 ☐ 방문일과 동일

☐ 방문일과 상이할 경우 체크 후, 우측에 날짜 기재

년 YYYY 월 MM 일 DD

**1.** 매우 우수	**2.** 우수	**3.** 불변	**4.** 악화	**5.** 매우악화

식이조사

식이조사지 작성을 확인하였습니까?

□ 예 □ 아니오 (사유: ____)

순응도 확인

배부된 2차 시험/대조 식품이 반납되었습니까? ☐ 예 ☐ 아니오(사유:)

섭취기간 기준은 '인체적용시험용 식품 처방날 ~ 방문4 전날' 입니다.

처방 식품 수	**4**주분 **+** 여유분	비 고
섭취해야 할 식품 수 [a]	________________ 캡슐	
섭취한 식품 수 [b]	________________ 캡슐	
남은 식품 수	________________ 캡슐	
분실된 식품 수	________________ 캡슐	
섭취율**(%) (= b/a *100)**	________________ %	반올림하여<br>소수점 1자리까지 표기

시험종결 페이지**(p.29)**를 작성하여 주십시오.

약물투여력/병용약물
(Drugs/Concomitant drugs)

Protocol No.: GRT_Bio_Ge

Screening No. Y G - S

약물 투여력 및 병용약물 조사

☞ 스크리닝 4주 이내~인체적용시험 기간 동안 복용한 약물 ☐ 없음

No.	약물명	1일 용량	단위 1)	경로 2)	투여시작일 (YYYY/MM/DD)		투여종료일 (YYYY/MM/DD)		지속 여부	투여목적
1					/ /		/ /		☐	
					V2이전 ☐	V2이후 ☐	V2이전 ☐	V2이후 ☐		
2					/ /		/ /		☐	
					V2이전 ☐	V2이후 ☐	V2이전 ☐	V2이후 ☐		
3					/ /		/ /		☐	
					V2이전 ☐	V2이후 ☐	V2이전 ☐	V2이후 ☐		
4					/ /		/ /		☐	
					V2이전 ☐	V2이후 ☐	V2이전 ☐	V2이후 ☐		
5					/ /		/ /		☐	
					V2이전 ☐	V2이후 ☐	V2이전 ☐	V2이후 ☐		
6					/ /		/ /		☐	
					V2이전 ☐	V2이후 ☐	V2이전 ☐	V2이후 ☐		
7					/ /		/ /		☐	
					V2이전 ☐	V2이후 ☐	V2이전 ☐	V2이후 ☐		
8					/ /		/ /		☐	
					V2이전 ☐	V2이후 ☐	V2이전 ☐	V2이후 ☐		
9					/ /		/ /		☐	
					V2이전 ☐	V2이후 ☐	V2이전 ☐	V2이후 ☐		

* 다음에 해당되는 자는 본 연구에 참여하실 수 없습니다.
- 스크리닝 전 3개월 이내에 당뇨로 인해 약제를 새로 시작하는 자
- 스크리닝 전 3개월 이내에 예방접종을 실시한 자
- 스크리닝 전 2주 이내에 면역에 영향을 미칠 수 있는 건강기능식품을 섭취했거나 섭취중인 자

* 병용금지약물/식품 및 시술
- 면역관련 건강기능식품 및 식품 (예: 홍삼, 인삼, 클로렐라, 알콕시글리세롤 함유 상어간유, 알로에 겔, 한약 등)
- 면역 관련된 예방접종 (예: Influenza 등)

약물투여력/병용약물 확인 기재 방법

1) 약물명은 단일성분인 경우에는 반드시 성분명으로 기록하여 주시고, 복합제인 경우 제품명을 기록하여 주십시오.
2) 투여경로 및 단위의 경우 아래 분류번호를 참조하여 기록해 주시고, 1일 용량, 단위, 투여기간, 투여목적 등을 정확히 기록하여 주십시오.
3) 투여일에서 정확한 날짜를 알 수 없는 경우에는 UK로 표시하여 주십시오. (예:2016/09/UK)
4) 건강기능식품까지 모두 기재하여 주십시오. (예: 비타민제, 글루코사민 등)

*분류번호 1) 단위

1	g (gram)	2	mg (milligram)	3	cap (capsule)	4	ml (milliliter)	5	T (Tab)	6	IU (International unit)	7	기타 (직접기록)

*분류번호 2) 경로

01	경구(PO)	02	설하(SL)	03	근육주사(IM)	04	정맥주사(IV)	05	피하주사(SC)
06	흡입(Inhalation)	07	피부도포	08	점막도포	09	직장투여	10	기타(직접기록)

☐ 추가 약물투여력/병용약물 페이지 있음

이상반응 (Adverse events)

Protocol No.: GRT_Bio_Ge

Screening No. Y G - S

이상반응 (Adverse events) ☐ 없음

	이상반응 1	이상반응 2	이상반응 3	이상반응 4
진 단				
면역관련 여 부	☐ 아니오<br>☐ 예	☐ 아니오<br>☐ 예	☐ 아니오<br>☐ 예	☐ 아니오<br>☐ 예
발 현 일	년 YYYY 월 MM 일 DD	년 YYYY 월 MM 일 DD	년 YYYY 월 MM 일 DD	년 YYYY 월 MM 일 DD
소 실 일	년 YYYY 월 MM 일 DD	년 YYYY 월 MM 일 DD	년 YYYY 월 MM 일 DD	년 YYYY 월 MM 일 DD
이상반응 정 도	☐ 경도 (Mild)<br>☐ 중등도 (Moderate)<br>☐ 중증 (Severe)	☐ 경도 (Mild)<br>☐ 중등도 (Moderate)<br>☐ 중증 (Severe)	☐ 경도 (Mild)<br>☐ 중등도 (Moderate)<br>☐ 중증 (Severe)	☐ 경도 (Mild)<br>☐ 중등도 (Moderate)<br>☐ 중증 (Severe)
중 대 한 이상반응 여 부	☐ 아니오<br>☐ 예	☐ 아니오<br>☐ 예	☐ 아니오<br>☐ 예	☐ 아니오<br>☐ 예
이상반응 결 과	☐ 완전치유(후유증 없음)<br>☐ 치유(후유증 있음)<br>☐ 진행중<br>☐ 영구적 손상<br>☐ 사망	☐ 완전치유(후유증 없음)<br>☐ 치유(후유증 있음)<br>☐ 진행중<br>☐ 영구적 손상<br>☐ 사망	☐ 완전치유(후유증 없음)<br>☐ 치유(후유증 있음)<br>☐ 진행중<br>☐ 영구적 손상<br>☐ 사망	☐ 완전치유(후유증 없음)<br>☐ 치유(후유증 있음)<br>☐ 진행중<br>☐ 영구적 손상<br>☐ 사망
본제와의 인과관계	☐ 명확히 관련 있음 (Definitely related)<br>☐ 관련 있다고 생각됨 (Probably related)<br>☐ 관련 있을 가능성 있음 (Possibly related)<br>☐ 관련이 없다고 생각됨 (Probably not related)<br>☐ 명확히 관련이 없음 (Definitely not related)<br>☐ 불명(Unknown)	☐ 명확히 관련 있음 (Definitely related)<br>☐ 관련 있다고 생각됨 (Probably related)<br>☐ 관련 있을 가능성 있음 (Possibly related)<br>☐ 관련이 없다고 생각됨 (Probably not related)<br>☐ 명확히 관련 없음 (Definitely not related)<br>☐ 불명(Unknown)	☐ 명확히 관련 있음 (Definitely related)<br>☐ 관련 있다고 생각됨 (Probably related)<br>☐ 관련 있을 가능성 있음 (Possibly related)<br>☐ 관련이 없다고 생각됨 (Probably not related)<br>☐ 명확히 관련 없음 (Definitely not related)<br>☐ 불명(Unknown)	☐ 명확히 관련 있음 (Definitely related)<br>☐ 관련 있다고 생각됨 (Probably related)<br>☐ 관련 있을 가능성 있음 (Possibly related)<br>☐ 관련이 없다고 생각됨 (Probably not related)<br>☐ 명확히 관련 없음 (Definitely not related)<br>☐ 불명(Unknown)
본 제 관련조치	☐ 없음<br>☐ 감량<br>☐ 일시중단 후 재투여<br>☐ 투여중단	☐ 없음<br>☐ 감량<br>☐ 일시중단 후 재투여<br>☐ 투여중단	☐ 없음<br>☐ 감량<br>☐ 일시중단 후 재투여<br>☐ 투여중단	☐ 없음<br>☐ 감량<br>☐ 일시중단 후 재투여<br>☐ 투여중단
교정치료 여부/내용	☐ 예↓ ☐ 아니오<br>________<br>________	☐ 예↓ ☐ 아니오<br>________<br>________	☐ 예↓ ☐ 아니오<br>________<br>________	☐ 예↓ ☐ 아니오<br>________<br>________

☐ 추가 이상반응 페이지 있음

시험종결
(Conclusion)

Screening No.

Y G - S

Protocol No.: GRT_ Bio_Ge

인체적용시험 기간

인체적용시험 시작일... 년 YYYY 월 MM 일 DD

인체적용시험 중단일/종료일... 년 YYYY 월 MM 일 DD

인체적용시험 종료 현황

☐ 1.스크리닝 탈락*

☐ 2.중도탈락*

☐ 3.시험종료

☞ 스크리닝/ 중도 탈락의 경우, 아래의 '탈락/위반 사유' 중 주요 사유 1개만 기재하여 주십시오.

탈락/위반 사유

☐ 1. 선정/제외기준 위반

☐ 2. 동의철회

☐ 3. 이상반응/ 중대한 이상반응 ☞ SAE 보고서 작성 ☐ 1.예 ☐ 2.아니오*

☐ 4. 추적실패

☐ 5. 시험책임자가 종료지시

☐ 6. 병용금지 약물 복용

☐ 7. 기타 ( )

눈가림해제 여부

본 인체적용시험대상자의 방문 기간 중 눈가림해제가 발생하였습니까?

☐ 1. 예 ☐ 2. 아니오

시험책임자 확인

본 인체적용시험대상자는 인체적용시험 기간 중 본인의 감독 하에 있었으며, 본 증례기록서에 포함된 정보와 자료는 완벽하며 정확합니다.

Comments: ______________________ 날 짜 : ______ 년 ______ 월 ______ 일

______________________ 서 명 : ______________________

추가방문
(Additional Visit)

Protocol No.: GRT_Bio_Ge

Screening No. Y G -S

방문일

방문일을 기록하여 주십시오. .... 2 0 __ __ / __ __ / __ __ (년 YYYY 월 MM 일 DD)

추가방문 사유

☐ 이상반응

☐ 중도탈락 ☞ 시험종결 페이지(p.29)를 작성하여 주십시오.

☐ 기타 ☞ 사유 ________________________________

이상반응 확인

지난번 방문 이후 새로운 의학적인 문제 또는 질병이 발생하였습니까?

☐ 예 ☞ 이상반응 페이지(p.28)를 작성하여 주십시오.

☐ 아니오

병용약물 및 병용요법 변화 확인

지난번 방문 이후 새로운 약물을 투여, 복용하였습니까?

☐ 예 ☞ 병용약물 및 병용요법 페이지(p. 27)를 작성하여 주십시오.

☐ 아니오

활력징후

☐ 미실시 (사유: ____________________)

검사일: ☐ 방문일과 동일

☐ 방문일과 상이할 경우 체크 후, 우측에 날짜 기재 __ __ __ __ / __ __ / __ __ (년 YYYY 월 MM 일 DD)

혈압 (mmHg)	___ Systolic / ___ Diastolic	맥박 (회/min)	___

추가방문
(Additional Visit)

Screening No.
Y G -S

Protocol No.: GRT_Bio_Ge

체온 측정

☐ 미실시 (사유: ____________________)

검사일: ☐ 방문일과 동일

☐ 방문일과 상이할 경우 체크 후, 우측에 날짜 기재 년 YYYY 월 MM 일 DD

체온 (℃) ___ . _

신체계측

☐ 미실시 (사유: ____________________)

검사일: ☐ 방문일과 동일

☐ 방문일과 상이할 경우 체크 후, 우측에 날짜 기재 년 YYYY 월 MM 일 DD

체중 (kg) ___ . _

☞ 체중은 0.1 kg 단위까지 반올림하여 기록해 주십시오.

유효성 검사

☐ 미실시 (사유:)

검사일 ☐ 방문일과 동일

☐ 방문일과 상이할 경우 체크 후, 우측에 날짜 기재 2 0 년 YYYY 월 MM 일 DD

항목	결과	항목	결과
**NK cell activity (%, 2.5:1)**		**IgG1(mg/dL)**	
**NK cell activity (%, 5:1)**		**IgG2(mg/dL)**	
**NK cell activity (%, 10:1)**		**IgM(mg/dL)**	
**NK cell activity (%, 50:1)**		**IFN-γ (pg/ml)**	
**IL-2 (pg/ml)**		**TNF-α (pg/ml)**	

추가방문
(Additional Visit)

Protocol No.: GRT_Bio_Ge

Screening No. Y G -S

**IL-6 (pg/ml)**		**WBC 수(x10^3 cells/uL)**	
**IL-12 (pg/ml)**			

시험대상자 자신에 의한 개선도 평가		☐ 미실시 (사유:		)
검사일 ☐ 방문일과 동일<br>☐ 방문일과 상이할 경우 체크 후, 우측에 날짜 기재			2 0 _ _ 년 YYYY _ _ 월 MM _ _ 일 DD	
1. 매우 우수	2. 우수	3. 불변	4. 악화	5. 매우악화

이학적 검사	☐ 미실시 (사유 : ______)	
신체기관명	정상여부	비정상인 경우 의학적 의견 기술
심혈관계	☐ 정상 ☐ 비정상	1. ______<br>2. ______
폐 및 호흡기계	☐ 정상 ☐ 비정상	1. ______<br>2. ______
위장관/간 및 담도계	☐ 정상 ☐ 비정상	1. ______<br>2. ______
대사/내분비계	☐ 정상 ☐ 비정상	1. ______<br>2. ______
신장/요로계	☐ 정상 ☐ 비정상	1. ______<br>2. ______
생식기계	☐ 정상 ☐ 비정상	1. ______<br>2. ______
근골격계	☐ 정상 ☐ 비정상	1. ______<br>2. ______

추가방문
(Additional Visit)

Protocol No.: GRT_Bio_Ge

Screening No. Y G -S

피부 및 결합조직	☐ 정상 ☐ 비정상	1. ______<br>2. ______
신경계	☐ 정상 ☐ 비정상	1. ______<br>2. ______
정신계	☐ 정상 ☐ 비정상	1. ______<br>2. ______
기타	☐ 정상 ☐ 비정상	1. ______<br>2. ______

* 새로운 증상이 나타나거나 질환이 악화된 경우 이상반응 페이지(p.29)를 작성하여 주십시오.

임신반응검사* * 임신가능성이 있는 가임기 여성의 경우에 한하여 실시합니다.

☐ 미실시 ☞ 사유 : ☐ 불임수술 ☐ 폐경 ☐ 남성 ☐ 기타 (______)

검사일: ☐ 방문일과 동일

☐ 방문일과 상이할 경우 체크 후, 우측에 날짜 기재 년 YYYY 월 MM 일 DD

검사결과 .... ☐ 음성 ☐ 양성 ☞ "양성"인 경우 본 인체적용시험에 참여할 수 없습니다.

추가방문
(Additional Visit)

Protocol No.: GRT_Bio_Ge

Screening No. Y G -S

임상병리검사

☐ 미실시 (사유: ____________________)

항목			검사치	재검치[1]	정상		임상적 의미		소견
검사일자(YYYY/MM/DD)			/ /	/ /	예	아니오[2]	예[3]	아니오	
혈액학적검사	WBC	x10³/uL			☐	☐	☐	☐	
	RBC	x10³/uL			☐	☐	☐	☐	
	Hb	g/dL			☐	☐	☐	☐	
	Hct	%			☐	☐	☐	☐	
	Platelet	10³/mm³			☐	☐	☐	☐	
	Lymphocyte	%			☐	☐	☐	☐	
	Monocyte	%			☐	☐	☐	☐	
혈액화학적검사	AST(GOT)	U/L			☐	☐	☐	☐	
	ALT(GPT)	U/L			☐	☐	☐	☐	
	Total Protein	g/dL			☐	☐	☐	☐	
	Glucose	mg/dL			☐	☐	☐	☐	
	Total cholesterol	mg/dL			☐	☐	☐	☐	
	BUN	mg/dL			☐	☐	☐	☐	
	Creatinine[6]	mg/dL			☐	☐	☐	☐	
	Uric acid	mg/dL			☐	☐	☐	☐	
	Ca	mg/dL			☐	☐	☐	☐	
	P	mg/dL			☐	☐	☐	☐	
소변검사	SG	-			☐	☐	☐	☐	
	pH	-			☐	☐	☐	☐	
	Protein	-			☐	☐	☐	☐	
	Glucose	-			☐	☐	☐	☐	
	Ketone	-			☐	☐	☐	☐	
	Bilirubin	-			☐	☐	☐	☐	
	Urobilinogen	-			☐	☐	☐	☐	
	Nitrite	-			☐	☐	☐	☐	
	WBC(Leukocyte)	-			☐	☐	☐	☐	
	Erythrocyte	-			☐	☐	☐	☐	

추가방문
(Additional Visit)

Protocol No.: GRT_Bio_Ge

Screening No. Y G -S

	1) 재검치가 기록된 경우 재검치에 의해 정상여부를 표기하고 임상적 의미를 판단하여 표기하십시오.<br>2) 정상여부 '아니오'인 경우 임상적 의미 여부에 표기하십시오.<br>3) 임상적의미 '예'인 경우 소견란에 기재하십시오<br>* 누락된 항목의 경우 검사결과란에 ND(Not Done)로 적고, 그 사유를 소견란에 기재하십시오.

식이조사

식이조사지 작성을 확인하였습니까?

☐ 예 ☐ 아니오 (사유: ____)

순응도 확인

지난 방문 시 배부된 시험/대조 식품이 반납되었습니까? ☐ 예 ☐ 아니오(사유:)

섭취기간 기준은 ' 인체적용시험용 식품 처방날 ~ 방문 전날' 입니다.

처방 식품 수	**4주분 + 여유분**	비 고
섭취해야 할 식품 수 [a]	____________________ 캡슐	
섭취한 식품 수 [b]	____________________ 캡슐	
남은 식품 수	____________________ 캡슐	
분실된 식품 수	____________________ 캡슐	
**섭취율(%) (= b/a *100)**	____________________ %	반올림하여 소수점 1자리까지 표기

시험을 지속한다면, 다음 방문일을 지정하여 주시고,
종결된다면 시험종결 페이지**(p.29)**를 작성해 주십시오.

중대한 이상반응 보고서

■ 의뢰사명	
■ 연구계획서 번호	
■ 연구용 식품	
■ 보고분류	초회보고□ 추적보고□ #____ (추적보고인 경우 초회/최근 보고일자: 년 월 일)

◈ 대상자 정보

■ 대상자 스크리닝번호		■ 대상자 배정번호		■ 대상자 이니셜	
■ 성별	■ 생년월일	■ 나이	■ 키 (cm)	■ 몸무게 (kg)	■ 진료구분
남자□ 여자□	(년/월/일)	만 세			입원□ 외래□ 기타 ( )
■ 임신여부(기간: 주)	마지막 월경일자(년 월 일)			해당없음□	

◈ 중대한 이상반응

이상반응명	날짜①		중등도②	연구용 식품과의 인과관계③	연구용 식품에 대한 처치④	관찰 결과⑤	이상반응 중대성 (Seriousness)⑥
	시작일	종료일					

① 필요시 지속기간, 의심약물 투여시작부터 반응 발현시작까지의 시간 병기

② 1.경도 2.중등도 3.중증

③ 1.명확히 관련 있음 2.관련 있다고 생각됨 3.관련 있을 가능성 있음 4.관련 없다고 생각됨 5.명확히 관련 없음 6.불명

④ 1.없음 2.감량 3.일시중단 후 재투여 4.투여중지 5.해당사항 없음

⑤ 1.회복됨 2.회복중임 3.회복되지 않음 4.회복되었으나 후유증이 남음 5.사망 6.알 수 없음

⑥ 1.사망 2.생명을 위협 3.입원 또는 입원기간의 연장 4.지속적 또는 의미있는 불구나 기능저하 초래 5.선천성 기형 또는 이상 초래 6.기타 의학적으로 중요한 상황

■ 대상자사망	예□ 아니오□ 사망일(년 월 일)	■ 보고된 사망원인	
■ 부검여부	예□ 아니오□ 알 수 없음□		

◈ 약물 정보

시험/대조 식품 (상품명/성분명)	1일 투여량	단위	투여 경로	섭취기간			사용중단/감량결과①	재섭취 결과②
				시작일	종료일	지속		
						☐		
						☐		
						☐		

병용약물 (이상반응 발생 7일 이전부터 발생시까지 투여한 약물, 이상반응 치료를 위한 약물 포함하지 않음)

☐ 없음

병용약물명	1일 투여량	단위	투여 경로	약물투여기간			투여목적	사용중단/ 감량결과 ①	재투약 결과②
				시작일	종료일	지속			
						☐			
						☐			
						☐			
						☐			
						☐			
						☐			

① 1.이상반응 소실/개선 2.소실/개선되지 않음 3.사용중단/감량하지 않음
② 1.이상반응 재발/악화 2.재발/악화되지 않음 3.재투약하지 않음

◈ 주요 병력 및 동반 질환

☐ 없음

질병/수술/기타	시작일	종료일	지속여부 (예/아니오/알수없음)	의견

◈ 이상약물반응 관련 약물 과거력

☐ 없음

약물명(보고된 대로)	투약시작일	투약종료일	적응증	이상약물반응

◈ 원인 규명을 위한 검사 결과

날짜	검사 종류	단위	정상<br>하한치	정상<br>상한치	결과	결과지 첨부 여부<br>(예/아니오)

◈ 증례기술, 추가 정보 또는 보고자의 의견

(※ 중대한 이상반응에 대해 누가, 무엇을, 언제, 어떻게, 왜 발생하였는지와 연구자의 인체적용시험용 식품과의 인과관계에 판단근거 등을 기술하시오.)

◈ 정보제공자

이 름		기관명		소속	
전 화		Fax		E-mail	

Full Version in Korean

1. Trial Study Protocol for IRB's Review
2. Case Report Form Designed for Clinical Trial
3. **Written Explanation to Participants**
4. Consent Form for Participation
5. Consent Form for Human Biologic Materials
6. Survey for Stress Response Inventory (SRI)
7. Survey for Nutrition Intake
8. Self-Assessment Form
9. Advertisement for Recruitment

연구참여자 설명문
(인간대상 연구용)
(ver. 1.1)

연구과제명 : 바이오게르마늄의 면역기능증진 효과 및 면역세포 활성화 효과 평가를 위한 인체적용시험

연구책임자(성명/소속): 이종호 / 연세대학교 생활과학대학 식품영양학과

전화번호: 02-313-8844

본 연구는 일반인을 대상으로 바이오게르마늄 효모를 섭취하였을 때 면역력 증진 효과를 검증하기 위한 연구입니다. 귀하는 본 연구에 참여할 것인지 여부를 결정하기 전에, 설명문과 동의서를 신중하게 읽어보셔야 합니다. 이 설명문에서는 이 연구가 왜 수행되며, 무엇을 수행하는지에 대해 설명하고 있습니다. 또한 언제든지 귀하가 이 연구 참여를 중단할 수 있음을 설명하고 있습니다.

이 연구를 수행하는 담당 연구원이 귀하에게 이 연구에 대해 설명해 줄 것입니다. 이 연구는 자발적으로 참여 의사를 밝히신 분에 한하여 수행될 것입니다. 다음 내용을 신중히 읽어보신 후 참여 의사를 밝혀 주시길 바라며, 필요하다면 가족이나 친구들과 의논해 보십시오.

만일 어떠한 질문이 있다면 담당 연구원이 자세하게 설명해 줄 것입니다. 귀하의 서명은 귀하가 본 연구에 대해 그리고 위험성에 대해 설명을 들었음을 의미하며, 이 문서에 대한 귀하의 서명은 귀하께서 자신(또는 법정대리인)이 본 연구에 참가를 원한다는 것을 의미합니다.

1. 연구의 배경과 목적

최근 들어 SARS나 조류독감 등과 같은 질병들로 인해 면역 기능 증진 관련 식품에 대한 관심도가 증대되었으며, 식생활의 향상과 의학의 발달로 인간의 평균 수명이 연장되고 문명의 발달에 따른 환경오염, 스트레스 및 운동부족이 원인이 되어 이전보다 더 심각한 병을 가진 채 장기간 생존하는 사람들이 많아지면서 면역력 저하 인구가 급증하고 있습니다. 게르마늄이라는 원소는 1886년 독일의 화학자 Clemen Winkler에 의해 최초로 발견되었으며 의학적인 효능이 처음 발견된 것은 1930년 프랑스와 스페인의 국경지방인 Lourdes의 샘물이 여러 가지 질병에 큰 효과가 있다고 알려져 샘물의 성분 분석 결과

게르마늄의 함량이 높아 치료 효과를 보인다고 보고되었으며 또한 여러 연구를 통해 토양, 물, 식물 그리고 동물 체내에 자연적으로 발견되는 미량 원소인 게르마늄이 항암, 항염증 및 면역 증강 기능 등 생리학적인 효능이 알려져 있습니다.
이에 세포 및 동물 실험, 임상시험을 통하여 바이오게르마늄 효모가 면역력 증진에 도움이 되는 것을 확인하였습니다. 또한 동물실험에서 바이오게르마늄 효모 2,000mg/kg을 13주간 투여하였을 때, 독성이 나타나지 않음을 확인하였습니다. 동물실험에서 안전성이 확인된 용량을 인체에게 적용하였을 때 1일 9,600mg 까지는 안전하다고 할 수 있으며, 따라서 본 연구의 바이오게르마늄 효모 일일섭취량인 1,200mg은 무해한 용량입니다.
따라서 본 연구에서는 일반인을 대상으로 바이오게르마늄 효모를 섭취하였을 때 면역력 유효성 지표(면역 관련 지표 검사, 전반적 평가)의 개선 정도를 대조식품과 비교하여 유의한 차이를 보이는지 검증하고자 합니다.

2. 연구 참여 대상

이 연구는 말초혈액 백혈구 수치 4×10^{3}cells/㎕~8×10^{3}cells/㎕에 해당하는 만 25세 이상~만 75세 이하의 성인남여 총 130명을 대상으로 시험군 65명, 대조군 65명이 참여 할 것입니다.

선정기준
① 만 25세 이상 ~ 만 75세 이하의 남, 여
② 선별 검사에서 측정한 말초혈액 백혈구 수치가 4X10^{3}이상 ~ 8X10^{3}cells/ul 미만인 자
③ 본 연구에 참여할 것을 동의하고 서면 동의서에 연구대상자(또는 법정 대리인)가 자의로 서명한 자

제외기준
① 임상적으로 유의한 급성 또는 만성 심뇌혈관계, 면역계, 호흡기계, 간담도계, 신장 및 비뇨기계, 신경계, 근골격계 질환, 정신성, 감염성 및 혈액·종양성 질환 등으로 현재 치료 중인 자 (단, 대상자의 상태를 고려하여 연구책임자의 판단에 따라 연구에 참여 가능하다.)
② 조절되지 않는 고혈압이 있는 자 (140/90mmHg 이상, 연구대상자 10분 안정 후 측정 기준)
③ 조절되지 않는 당뇨병 환자 (공복혈당 126mg/dl 이상 또는 3개월 이내에 당뇨로 인해 약제를 새로 시작하는 경우)
④ 선별검사 전 3개월 이내에 예방접종을 실시한 자
⑤ AST(GOT) 또는 ALT(GPT) 혈중치가 120 IU/L 초과한 자
⑥ Creatinine 혈중치가 남자 2.4 mg/dL, 여자 1.8 mg/dL 초과한 자
⑦ 선별검사 전 2주 이내에 면역에 영향을 미칠 수 있는 건강기능식품을 섭취했거나 섭취중인 자
⑧ 속쓰림, 소화불량 등의 심한 위장관 증상을 호소하는 자
⑨ 임신, 수유 중이거나 본 연구 기간 동안 임신 계획이 있는 자
⑩ 시험식품 관련 식품에 민감하거나 알레르기가 있는 자

⑪ 본 연구 중에 다른 연구에 참가할 계획이 있는 자
⑫ 본 연구 시작 4주 이내에 다른 연구에 참여했던 자
⑬ 연구자가 본 연구에 부적합하다고 판단하는 자

귀하가 시험대상자로 적합하다고 판정이 되실 경우에는 어느 섭취군에 배정되시는지는 컴퓨터의 프로그램으로 공정하게 무작위 (예 : 동전던지기로 앞/뒷면을 결정하는 방법)로 결정되어, 50%의 확률로 시험용 식품군 또는 대조용 식품군으로 나뉘어 배정됩니다. 즉 참여자의 절반은 대조용 식품군에 배정될 수 있다는 것입니다. 대조용 식품군은 본 연구에서 보고자 하는 시험용 식품(바이오게르마늄 효모)의 효과가 단순한 식품 섭취에 의한 것이 아니라는 것을 밝히는 과학적 연구를 위해 꼭 필요한 집단입니다. 대조용 식품도 시험용 식품과 같이 과학적 근거에 의해 인체에 무해한 식용 가능한 원료(옥수수전분, 식용색소 및 향료)로 제조되었음을 알려드립니다. 엄격한 과학적 절차를 통해 연구를 실시하기 위해 본 연구가 종료될 때까지 귀하는 물론 연구를 진행하는 연구자도 귀하가 어느 군에 속하는지 알 수 없습니다. 하지만 응급상황이 발생하면 귀하가 복용하는 식품을 파악하여 적절한 조치를 받으실 수 있습니다.

3. 연구방법

방문 시기	방문1 (선별검사)	방문2 (0주방문)	방문3 (4주방문)	방문4 (8주방문)
검사 시간	30분 이내	40분 ~ 1시간	40분	40분 ~ 1시간
검사 내용	신체계측<br>혈액학적 검사<br>실험실적 검사<br>소변 검사	면역지표 검사<br>면역 관련 유전자검사<br>식이조사 확인<br>스트레스 척도<br>설문조사 등	신체계측<br>이상반응 확인<br>면역지표 검사<br>식이조사 확인 등	신체계측<br>혈액학적 검사<br>이상반응 확인<br>면역지표 검사<br>실험실적 검사<br>소변 검사<br>식이조사 확인 등
혈액 검사	12시간 공복 상태로 방문하여 채혈	12시간 공복 상태로 방문하여 채혈	12시간 공복 상태로 방문하여 채혈	12시간 공복 상태로 방문하여 채혈

만일 귀하가 본 연구에 참여의사를 밝혀 주시면 다음과 같은 과정이 진행될 것입니다. 귀하는 본 동의설명서를 읽으시고 충분히 설명을 들으신 후 심사숙고 하셔서 동의서에 서명하시고, 이번 연구에 참여하시게 되면 약 10주의 연구 기간을 거치시게 됩니다. 연구기간 동안 귀하는 총 4회 (선별검사, 0주, 4주, 8주) 방문을 하시게 됩니다.

방문1 (선별검사)에서는 먼저 귀하가 본 연구 참여에 동의하시면 인구학적 배경 (성별, 나이 등) 및 기타 질환력과 치료력 등을 조사한 뒤 2주 이내의 선별 기간을 가지게 됩니다. 선별 기간 후 본 연구 참여에 적합한 것으로 판정되면 8주간 시험식품 또는 대조식품을 처방 받아 섭취방법에 따라 섭취하시게 됩니다. 선별방문 시 실험실적 검사, 신체계측 등이 이루어질 것이며 말초혈액 백혈구 수치 검사, 혈액학적 및 혈액화학적검사와 2차 연구를 위해 혈액 11mL, 소변 5mL가 수집될 것입니다. 단 연구대상자가 2차 연구를 위한 제공여부에 동의하지 않을 경우 연구대상자의 추가분석을 위한 샘플은 수집되지 않습니다.

방문2 (0주방문)에는 스트레스 척도 설문조사, 면역지표 검사, 면역 관련 유전자 검사 등이 이루어질 것이며 혈액 18mL가 수집될 것입니다. 시간은 40분~1시간 정도 소요될 예정입니다.

방문3 (4주방문)에는 활력징후, 이상반응 검사, 말초혈액 백혈구 수치 검사, 면역지표 검사 등이 이루어질 것이며 혈액 19mL가 수집될 것입니다. 시간은 약 40분~1시간 정도 소요될 예정입니다.

방문4 (8주방문)에는 말초혈액 백혈구 수치 검사, 혈액학적 및 혈액화학적검사, 면역지표 검사, 실험실적 검사, 2차 연구를 위한 검체 수집 및 이상반응 검사 등이 이루어질 것이며 혈액 27mL, 소변 5mL이 수집될 것이며 각각 40분~1시간 정도 소요될 예정입니다.

본 연구에서 수집하는 검체는 시험식품의 안전성을 확인하기 위해 이상반응(부작용)의 유무를 분석하는 목적과 유효성을 검증하기 위한 목적으로 실시하며, 매 방문 시 혈액검사 전 최소 12시간 금식이 필요합니다.
선별 검사를 포함하여 총 채혈 4회와 소변검사 2회가 실시됩니다. 채혈은 연세대학교 산하 임상영양유전연구실로 방문하여 담당 연구병리사를 통해 이루어질 예정이며, 채혈 방법은 팔에 있는 정맥으로부터 바늘을 이용하여 채혈할 예정입니다.

본 연구에서 수집되는 혈액 총 75mL(방문1 11mL, 방문2 18mL, 방문3 19mL, 방문4 27mL)는 전혈, 혈장, 혈청, 적혈구로 분리하여 -80℃에 보관하고, 소변 총 10mL(방문1 5mL, 방문4 5mL)는 -20℃에 보관할 예정입니다.
연구의 목적으로 수집된 검체는 직접적으로 신원을 확인할 수 없는 방법으로 보관되고, 연구 과정에서 사용 후 남은 검체는 인체유래물 연구 동의서에 동의를 한 경우에만 참여자의 동의내용(연구목적, 종류와 수량, 보존기간, 2차 사용 승낙여부, 개인식별정보)에 따라서 관리된 후에 사용됩니다. 인체유래물 연구에 동의하지 않는 경우에는 본 연구를 위하여 사용된 후에, 남은 검체는 연세대학교 관련 규정 절차에 따라 폐기합니다. (연구 참여 중도 탈락의 경우에도 위의 사항이 똑같이 적용됩니다. 단, 연구 참여 동의를 철회

한 경우에는 동의 철회 즉시 수집된 검체는 혈액 의료 폐기물로 분류 후 의료 폐기물 전용 용기에 담아서 폐기합니다.)

■ 방문 별 채혈량

항 목		방문1 (선별검사)	방문2 (0주방문)	방문3 (4주방문)	방문4 (8주방문)
임상병리 검사	혈액학검사	약 3mL	-	-	약 3mL
	혈액화학검사	약 6mL	-	-	약 6mL
유효성 검사	WBC 수	-	-	약 3mL	-
	NK cell activity	-	약 9mL	약 9mL	약 9mL
	IFN-γ, IL-2, 6, 12, TNF- α, IgG1, IgG1, IgM	-	약 7mL	약 7mL	약 7mL
면역 관련 유전자 검사		-	약 2mL	-	-
2차 연구를 위한 검사		약 2mL	-	-	약 2mL
**각 방문별 채혈량**		**약 11mL**	**약 18mL**	**약 19mL**	**약 27mL**
**총 채혈량**					**약 75mL**

연구 참여 기간 동안에 귀하는 시험식품 이외에 다른 건강기능식품이나 의약품의 복용을 중지하시고 시험용 식품 또는 대조용 식품을 섭취하여야 합니다. 섭취 후 귀하가 경험 하시게 되는 어떤 바람직하지 못한 의학적 변화는 모두 연구자 또는 연구담당자에게 알려주셔야 하며, 귀하는 4주 간격으로 정기적인 방문을 하시게 됩니다. 모든 방문 시에는 귀하가 연구기간 동안 섭취하시고 남은 시험식품을 반드시 가져 오셔야 합니다.

식이조사지는 연구 참여기간 동안 주중 이틀, 주말 하루 고르셔서 작성하시어 각각 방문 3(4방문), 방문 4(8주방문)시 가져와 주셔야합니다. 만일 연구 종료 방문 시 또는 중도탈락 방문 시까지 지속되고 해결되지 않은 이상반응이 있다면 경과 관찰을 위하여 2주 후에 다시 한 번 방문하시게 될 수 도 있습니다. 본 연구 참여를 완료하시면 혈액 및 소변 검사 등 검사 결과는 연구가 종료 된 후 1~2 달 이내에 댁으로 등기발송 해드립니다.

4. 연구 참여 기간

귀하는 본 연구를 위해 약 8주 동안 총 4회(선별검사, 0주, 4주, 8주) 방문하시게 됩니다. 매 방문 시 검사 및 대기시간을 포함하여 약 1시간~2시간 정도가 소요 될 수 있습니다. 검사 결과에 대한 확인 절차가 예정보다 늦어질 수 있으며, 연구 기간 중 부작용(이상반응) 발생 및 처치에 따르는 경과 등에 따라 별도로 추가 방문 할 수 있습니다. 추가방문은 예정된 방문 외에 인체적용시험 진행 중 필요한 경우, 예를 들어 이상반응의 발생여부가 의심되거나 이상반응 발생 후 추적조사가 필요한 경우, 중도탈락 등 인체적용시험 계획서에서 계획된 방문과는 별도로 이루어질 수 있으며, 이로 인해 계획된 연구 참여 기간보다 기간이 다소 길어지거나 짧아질 수 있습니다.

5. 연구 참여 도중 중도탈락

본 연구에 참여하는 것은 자의이며, 연구대상자 자신이 자발적으로 참여를 결정할 수 있습니다. 만일 연구에 대한 설명을 듣고 참여에 동의하지 않더라도 전혀 문제가 되지 않습니다. 또한 본 연구 기간 중 연구대상자가 원할 경우 언제라도 연구 참여 동의를 철회할 수 있으며, 이로 인하여 연구를 중단하게 되더라도 추후 어떠한 불이익도 받지 않을 것임을 보장합니다. 만일 연구대상자 자신이 연구에 참여하는 것을 그만두고 싶다면 담당 연구원이나 연구책임자에게 즉시 말씀해주십시오.
그리고 다음의 경우에는 이 연구를 계속 진행하지 못하고 귀하는 탈락하시게 됩니다.

① 선정기준/제외기준에 위배된 경우
② 면역 관련 건강기능 식품 및 의약품을 섭취하는 경우
③ 중대한 이상반응(Serious Adverse Events) 혹은 이상반응(Adverse Events)으로 인하여 연구대상자가 연구 중단을 요구하는 경우
④ 섭취 전 검사에서 발견치 못한 전신질환이 발견된 연구대상자
⑤ 연구 기간 중 만족스럽지 못한 치료 효과로 인하여 연구대상자(또는 법정 대리인)가 연구 중단을 요구하는 경우
⑥ 연구대상자(또는 법정 대리인)가 연구 참가 동의를 철회한 경우
⑦ 연구대상자의 추적이 안되는 경우
⑧ 연구대상자에게 시험식품을 섭취하는데 문제가 있는 경우
⑨ 연구 기간 동안 담당의사의 지시 없이 연구 결과 판정에 영향을 미칠 수 있는 약물 및 식품 등을 투여한 경우
⑩ 연구자의 판단에 의해 연구의 진행이 적합하지 못하다고 판단되는 경우
⑪ 임신이 확인되는 경우

6. 부작용 또는 위험요소

바이오게르마늄 효모 1.2g을 2개월간 섭취 시킨 이전 인체적용시험결과에서 혈액학적 검사 및 혈액 화학적 검사에서 특별한 이상반응이나 부작용은 발생하지 않았습니다. 특히 혈액검사 상 신장과 간의 기능과 관련된 ALT, AST, ALP, BUN, 크레아틴, 총빌리루빈 및 빈혈지표인 헤모글로빈, 헤마토크릿, 적혈구에도 어떠한 변화가 나타나지 않는 것으로 보아 바이오게르마늄 효모의 1일 1.2g의 섭취는 안전한 것으로 판단됩니다. 또한 본 원료는 1990년대부터 판매되어 온 제품으로 20년 넘게 유통되었고 크게 문제된 사례가 없던 것으로 알려져 있습니다. 다만, 본 시험식품은 천연물 소재로 경미한 소화불량이나 복통이 있을 수 있으며, 발효균주인 효모에 알레르기가 유발될 수 있습니다. 부작용은 사람에 따라 다양하며, 귀하는 이들 부작용을 모두 또는 일부를 경험하거나 전혀 경험하지 않을 수도 있습니다. 본 연구에 참여하는 동안, 귀하는 병용금지로 인한 불편 및 위험이 있을 수 있으며 예측하지 못한 부작용을 경험할 수도 있습니다. 또한 본 연구에 참여함으로써 받게 되는 어떤 시술이나 절차는 예상치 못하는 위험을 수반할 수 있습니다. 연구자는 이러한 사항에 대해 귀하에게 설명할 것입니다. 귀하가 부작용을 경험한다면, 연구자에게 알려서 귀하의 고통을 치료하거나 귀하가 경험한 불편을 해소하기 위해 다른 약물을 제공받으실 수 있습니다. 연구자는 시험식품의 투여를 일시 중단할 수 있으며, 중증 반응이 발생하는 경우, 연구자는 연구 참여를 영구적으로 중단시킬 수 있습니

다. 조기 탈락이 발생하게 되면, 종료방문(방문4)에 해당하는 평가 및 처치를 시행할 수 있습니다.
귀하의 전신적인 건강상태 및 유효성을 평가하기 위해 매 방문 시 혈액을 채취하게 되며, 혈액을 채취할 때 통증, 멍, 머리가 아찔함이 동반될 수 있습니다. 혈액 채취 후 지혈이 적절하게 되지 않은 경우는 멍이 생길 수 있으며, 저림 또는 감염으로 인한 염증이 발생할 수 있습니다. 멍은 얼음찜질로 증상을 감소시킬 수 있으나, 자연적으로 소실되므로 염려하지 않아도 됩니다. 혈액 채취로 인한 불편함을 최소화하기 위하여 연구자들은 항상 주의를 기울일 것이며, 감염으로 인한 염증이 불편함을 유발할 시에는 의료진의 처치를 받도록 할 것입니다.

7. 연구 참여에 따른 이익

본 연구 참여 시 직접적인 이익은 없습니다. 귀하는 시험식품에 대해 긍정적인 효과를 얻을 수도 있고, 그렇지 않을 수도 있습니다. 하지만 본 연구를 통해 얻어진 정보는 향후 다른 사람들을 위한 새로운 치료법이나 건강기능식품개발에 도움을 줄 수 있습니다. 그리고 보상으로 연구 참여 횟수에 따라 일정 금액의 교통비를 드립니다. 귀하께서 본 연구 참여를 완료하시면 교통비 등의 실비로 귀하에게 총 20만원이 지급될 것입니다. 중도포기 및 탈락이 되실 경우에는 교통비 전액이 아닌 방문한 횟수(회당 5만원)에 따라 교통비가 지급될 것입니다.

8. 연구에 참여하지 않을 시 불이익

귀하는 본 연구에 참여하지 않을 자유가 있습니다. 또한 귀하가 본 연구에 참여하지 않아도 귀하에게는 어떠한 불이익도 없습니다. 연구 참여 동의 철회 시, 귀하의 개인정보 등의 자료는 연구 종료 후 즉시 파쇄처리 될 것이며, 수집된 검체(혈액, 소변)는 동의 철회 즉시 혈액 의료 폐기물로 분류 후 의료 폐기물 전용 용기에 담아서 폐기합니다.

9. 상해에 대한 보상

연구 기간 중 연구자는 귀하의 안전을 지키려고 최선을 다해 노력할 것이고, 중대한 이상반응 발생 시 빠르고 적절한 조치를 취하여 가능한 이상반응을 최소화 하도록 노력할 것입니다. 이 연구에 참여하면서 시험식품 섭취와 관련된 피해가 발생할 경우에는 '피해자 보상에 관한 규약'에 의거하여 피해 보상할 것이며, 이상반응 및 질환악화의 경우에는 가능한 한 최선의 치료방법으로 치료받게 될 것입니다.

10. 개인정보와 비밀보장

본 연구에 참여하는 동안 수집되는 귀하의 개인정보는 다음과 같습니다. 성함, 성별, 생년월일, 연령, 연락처 등입니다. 연구를 위해 수집된 정보는 개인정보보호법에 따라 적절히 관리됩니다. 연구에 참여하는 동안 수집되는 귀하의 기록은 비밀로 보장되며, 연구의 결과가 보고서로 작성되거나 출판 또는 발표되는 경우에도 귀하의 신원을 파악할 수 있

는 기록은 비밀 상태로 유지될 것입니다. 귀하가 별도로 제공한 통장사본, 신분증 사본(개인정보제공 및 활용 동의서 내 서명 포함)은 연구를 위한 목적으로 사용되지 않습니다. 이 정보는 사례비 지급을 위한 것이므로 봉투에 바로 밀봉하여 연세대학교 산학협력단에 원본이 제공되며, 연구자가 보관하지는 않습니다. 산학협력단에 제공된 정보는 관련 법률에 의해 보관될 것이며, 입금확인을 위해 이름 및 전화번호 등은 일시적으로 보관할 수 있으나 입금확인 후 즉시 폐기할 것입니다. 그러나 만일 법이 요구하면 귀하의 개인정보는 제공될 수도 있습니다. 본 연구를 모니터/점검하는 자, 생명윤리심의위원회(IRB) 및 식품의약품안전처장은 귀하의 비밀 보장을 침해하지 않고 관련 규정이 정하는 범위 안에서 연구의 실시 절차와 자료의 신뢰성을 검증하기 위해 연구결과를 직접 열람할 수 있습니다. 귀하가 본 동의서에 서명하는 것은 이러한 사항에 대하여 사전에 알고 있었으며 이를 허용한다는 동의로 간주될 것입니다. 연구 종료 후 연구관련 자료(참여중지 및 철회 자료 포함)는 5년간 보관되며 이후 파쇄처리 될 것입니다. 연구 참여 동의를 철회한 경우 귀하의 개인정보 등의 자료는 연구 종료 시 즉시 파쇄처리 될 것입니다. 동의 철회 이외의 사유로 중도탈락 되시는 경우 귀하의 개인정보 등의 자료는 연구 종료 후 5년간 보관되며 이후 파쇄처리 될 것입니다.

11. 연구 문의

본 연구에 대해 질문이 있거나 연구 중간에 문제가 생길 시 다음 연구 담당자에게 언제든지 연락하십시오.

연구담당자 성명: 조정민 010.9495.8061
연구실 전화번호: 02.313.8844

연구참여자로서 귀하의 권리에 대하여 문제가 발생된 경우에는 연구자에게 말씀하시거나 다음의 번호로 문의하실 수 있습니다. 본 연구는 연세대학교 생명윤리위원회에서 윤리적, 과학적으로 승인된 연구입니다.

연세대학교 생명윤리위원회
☎ 02-2123-5143

Full Version in Korean

1. Trial Study Protocol for IRB's Review
2. Case Report Form Designed for Clinical Trial
3. Written Explanation to Participants
4. **Consent Form for Participation**
5. Consent Form for Human Biologic Materials
6. Survey for Stress Response Inventory (SRI)
7. Survey for Nutrition Intake
8. Self-Assessment Form
9. Advertisement for Recruitment

연 구 참 여 자 동 의 서

연구제목 : 바이오게르마늄의 면역기능증진 효과 및 면역세포 활성화 효과 평가를 위한 인체적용시험

아래의 내용을 읽으시고 내용을 완전히 이해하시면 네모 칸에 표시하여 주십시오.

☐ 본인은 이 설명문을 읽었고, 내용을 충분히 이해합니다.

☐ 본인은 자세하게 설명을 듣고 궁금한 사항에 대해 질문을 하였고 적절한 답변을 들었습니다.

☐ 본인은 자발적으로 이 연구에 참여합니다.

☐ 본인은 연구기간 중 언제라도 중도에 연구참여를 거부하거나 중단할 수 있습니다. 또 본인은 이 연구참여를 중단하더라도 본인에게 어떠한 불이익도 없다는 것을 알고 있습니다.

☐ 본인은 자유로운 의사에 따라 연구참여를 요청하여 설명문 및 작성된 동의서 사본 1부를 수령 합니다.

**연구참여자의 성명**	**서명**	**날짜 (년/월/일)**

**설명한 연구자의 성명**	**서명**	**날짜 (년/월/일)**

연구기간 20○○ ○○ ○○ - 20○○ ○○ ○○

YONSEI UNIVERSITY IRB

Full Version in Korean

1. Trial Study Protocol for IRB’s Review
2. Case Report Form Designed for Clinical Trial
3. Written Explanation to Participants
4. Consent Form for Participation
5. **Consent Form for Human Biologic Materials**
6. Survey for Stress Response Inventory (SRI)
7. Survey for Nutrition Intake
8. Self-Assessment Form
9. Advertisement for Recruitment

인체유래물 연구 동의서

동의서 관리번호	

(앞쪽)

인체유래물 기증자	성 명	생년월일
	주 소	
	전화번호	성별

법정대리인	성 명	관계
	전화번호	

연구책임자	성 명 이종호 (연세대학교 생활과학대학 식품영양학과 교수)
	전화번호 (02)313-8844

이 동의서는 귀하로부터 수집된 인체유래물등(인체유래물과 그로부터 얻은 유전정보를 말합니다)을 질병의 진단 및 치료법 개발 등의 연구에 활용하기 위한 것입니다. 동의는 자발적으로 이루어지므로 아래의 내용을 읽고 궁금한 사항은 상담자에게 묻고 질문할 기회를 가지고 충분히 생각한 후 결정하시기 바라며, 이 동의서에 대한 동의 여부는 귀하의 향후 검사 및 치료 등에 어떤 영향도 미치지 않습니다.

1. 인체유래물이란 인체로부터 수집하거나 채취한 조직·세포·혈액·체액 등 인체 구성물 또는 이들로부터 분리된 혈청, 혈장, 염색체, DNA, RNA, 단백질 등을 말하며, 귀하의 인체유래물을 채취하기 전에 채취 방법 및 과정에 관한 설명을 충분히 들어야 합니다.
2. 귀하가 귀하의 인체유래물등을 아래의 연구 목적에 이용하도록 동의하는 경우, 귀하의 인체유래물등의 보존기간, 다른 사람 또는 다른 연구 목적에 대한 제공 여부, 제공 시 개인정보 처리에 관한 사항 및 폐기 등을 결정할 수 있습니다. 또한 동의한 사항에 대해 언제든지 동의를 철회할 수 있습니다. 이 경우 연구의 특성에 따라 철회 전까지 수집된 귀하의 인체유래물등과 기록 및 정보 등의 처리방법이 달라질 수 있으므로 연구자로부터 별도의 설명문 등을 통해 정보를 받으실 것입니다.
3. 귀하는 이 연구 참여와 관련하여 귀하의 동의서 및 귀하의 인체유래물등의 제공 및 폐기 등에 관한 기록을 본인 또는 법정대리인을 통하여 언제든지 열람할 수 있습니다.
4. 귀하가 결정한 보존기간이 지난 인체유래물은 「폐기물관리법」 제13조에 따른 기준 및 방법에 따라 폐기되며, 해당 기관의 휴업·폐업 등 해당 연구가 비정상적으로 종료될 때에는 법에서 정한 절차에 따라 인체유래물등을 이관할 것입니다.
5. 귀하의 인체유래물등을 이용하는 연구는 「생명윤리 및 안전에 관한 법률」에 따라 연세대학교 생명윤리심의위원회의 승인 후 진행될 것이며 해당 기관 및 연구자는 귀하의 개인정보 보호를 위하여 필요한 조치를 취할 것입니다.
6. 귀하의 인체유래물등을 이용한 연구결과에 따른 새로운 약품이나 진단도구 등 상품개발 및 특허출원 등에 대해서는 귀하의 권리를 주장할 수 없으며, 귀하가 제공한 인체유래물등을 이용한 연구는 학회와 학술지에 연구자의 이름으로 발표되고 귀하의 개인정보는 드러나지 않을 것입니다.

※ 위의 모든 사항에 대해 충분한 설명을 듣고, 작성된 동의서 사본을 1부 받아야 합니다.

동의 내용	연구 목적	바이오게르마늄의 면역기능증진 효과 및 면역세포 활성화 효과 평가를 위한 인체적용시험
	인체유래물 종류 및 수량	혈액: 스크리닝 11mL, 0주 18mL, 4주 19mL, 8주 27 mL<br>소변: 스크리닝 5mL, 8주 5mL
	인체유래물 보존기간	1. 영구보존 [ ]<br>2. 동의 후 [ ] 년
	보존 기간 내 2차적 사용을 위한 제공 여부	1. 유사한 연구 범위 안에서만 제공하는 것에 동의합니다. [ ]<br>2. 포괄적 연구 목적으로 제공하는 것에 동의합니다 [ ]<br>3. 동의하지 않습니다. [ ]
	2차적 사용을 위한 제공 시 개인식별정보 포함 여부	1. 개인식별정보 포함 [ ]<br>2. 개인식별정보 불포함 [ ]

바이오게르마늄의 면역기능증진 효과 및 면역세포 활성화 효과를 평가하기 위한 연구 목적으로 수집된 인체유래물 (혈액 및 소변)은 직접적으로 신원을 확인할 수 없는 방법으로 연세대학교 식품영양학과 임상영양유전연구실에 보관될 것입니다. 연구 과정에서 획득된 인체유래물은 향후 본 연구 이외의 대사체 분석 등 다른 학술연구에 이용될 수 있습니다. 본 연구 분석 후 남은 혈액 및 소변은 인체유래물 동의서에 의해 연구대상자가 지정한 기간까지 보관할 예정이며, 본 연구 직후 폐기를 원하는 경우 또는 지정기간이 경과한 이후에는 혈액 의료 폐기물로 분류 후 의료 폐기물 전용 용기에 담아서 폐기합니다.

본인은 「생명윤리 및 안전에 관한 법률」 제37조 및 같은 법 시행규칙 제34조에 따라 해당 인체유래물연구의 목적 등 연구 참여와 관련하여 인체유래물 채취 방법 및 과정 등에 대한 동의서의 내용에 대하여 충분한 설명을 들어 이해하였으므로 위와 같이 본인의 인체유래물등을 기증하는 것에 자발적인 의사로 동의합니다.

동의서 작성일 년 월 일

인체유래물 기증자 (서명 또는 인)

법정대리인 (서명 또는 인)

상담자 (서명 또는 인)

구비서류	법정대리인의 경우 법정대리인임을 증명하는 서류

연구기간 20○○ ○○ ○○ - 20○○ ○○ ○○

YONSEI UNIVERSITY IRB

Full Version in Korean

1. Trial Study Protocol for IRB's Review
2. Case Report Form Designed for Clinical Trial
3. Written Explanation to Participants
4. Consent Form for Participation
5. Consent Form for Human Biologic Materials
6. **Survey for Stress Response Inventory (SRI)**
7. Survey for Nutrition Intake
8. Self-Assessment Form
9. Advertisement for Recruitment

SRI 설문지

(스트레스 반응 척도)

**평가일**	20 년 월 일
**스크리닝 번호**	Y G – S<br>기관코드 Screening No.
**시험대상자 이니셜**	
**작성자 서명**	

※ 다음 문항들은 여러분이 일상생활에서 스트레스를 받았을 때 경험할 수 있는 것들입니다. 각 문항을 주의 깊게 읽으면서 오늘을 포함하여 **지난 일주일(7일)** 동안에 어느 정도로 경험했는지를 해당되는 **칸에 O 표**를 하십시오.

너무 오랫동안 생각하지 마시고 될 수 있으면 빨리 응답하시기 바랍니다 그리고 문항을 하나도 빠뜨리지 말고 **반드시 한 곳에만 표시**하십시오.

**점수**	/156

**S R I**					
문항	전혀 그렇지 않다	약간 그렇다	웬만큼 그렇다	상당히 그렇다	아주 그렇다
1. 일에 실수가 많다.	0	1	2	3	4
2. 말하기 싫다.	0	1	2	3	4
3. 가슴이 답답하다.	0	1	2	3	4
4. 화가 난다.	0	1	2	3	4
5. 안절부절못한다.	0	1	2	3	4
6. 소화가 안된다.	0	1	2	3	4
7. 배가 아프다.	0	1	2	3	4
8. 소리를 지르고 싶다.	0	1	2	3	4
9. 한숨이 나온다.	0	1	2	3	4
10. 어지럽다.	0	1	2	3	4
11. 만사가 귀찮다.	0	1	2	3	4
12. 잡념이 생긴다.	0	1	2	3	4
13. 쉽게 피로를 느낀다.	0	1	2	3	4
14. 온몸에 힘이 빠진다.	0	1	2	3	4
15. 자신감을 잃었다.	0	1	2	3	4
16. 긴장된다.	0	1	2	3	4
17. 몸이 떨린다.	0	1	2	3	4
18. 누군가를 때리고 싶다.	0	1	2	3	4
19. 의욕이 떨어졌다.	0	1	2	3	4

20. 울고 싶다.	0	1	2	3	4
21. 신경이 날카로워졌다.	0	1	2	3	4
22. 내가 하는 일에 전망이 없다.	0	1	2	3	4
23. 멍하게 있다.	0	1	2	3	4
24. 누군가를 미워한다.	0	1	2	3	4
25. 한가지 생각에서 헤어나지 못한다.	0	1	2	3	4
26. 목소리가 커졌다.	0	1	2	3	4
27. 마음이 급해지거나 일에 쫓기는 느낌이다.	0	1	2	3	4
28. 행동이 거칠어졌다(난폭운전, 욕설, 몸싸움 등).	0	1	2	3	4
29. 무엇인가를 부수고 싶다.	0	1	2	3	4
30. 말이 없어졌다.	0	1	2	3	4
31. 머리가 무겁거나 아프다.	0	1	2	3	4
32. 가슴이 두근거린다.	0	1	2	3	4
33. 누군가를 죽이고 싶다.	0	1	2	3	4
34. 얼굴이 붉어지거나 화끈거린다.	0	1	2	3	4
35. 지루하다.	0	1	2	3	4
36. 참을성이 없다.	0	1	2	3	4
37. 얼굴표정이 굳어졌다.	0	1	2	3	4
38. 나는 아무 쓸모가 없는 사람이다.	0	1	2	3	4
39. 움직이기 싫다.	0	1	2	3	4

Full Version in Korean

1. Trial Study Protocol for IRB’s Review
2. Case Report Form Designed for Clinical Trial
3. Written Explanation to Participants
4. Consent Form for Participation
5. Consent Form for Human Biologic Materials
6. Survey for Stress Response Inventory (SRI)
7. **Survey for Nutrition Intake**
8. Self-Assessment Form
9. Advertisement for Recruitment

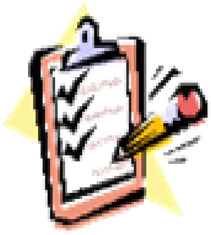

식이조사지 (방문2)

**방문일**	20 ____년 ____월 ____일
**스크리닝 번호**	Y G - S ___ ___ ___<br>기관코드 Screening No.
**인체적용시험대상자 이니셜**	___ ___ ___

* 작성 예

식사	식사시간<br>(소요시간)	음식명	재료명	음식량	식사장소
아침	7:00 시경<br>(30 분)	토스트<br>잼<br>계란후라이<br>우유	식빵<br>잼<br>계란<br>식용유<br>우유	3 장<br>2 찻술<br>1 개<br>1 작은술<br>1 잔(200ml)	식탁
간식	11:00 시경<br>(10 분)	크래커	크래커	3 개(3X4cm)	거실에서<br>TV 보면서
점심	12:00 시경<br>(20 분)	쌀밥<br>된장찌개<br>갈치구이<br>김<br>김치	밥<br>감자<br>호박<br>된장<br>갈치<br>식용유<br>김<br>배추김치	1 공기(210g)<br>1/3 개<br>1/2 개<br>1 큰술<br>중 1 토막<br>1 작은술<br>4 장<br>1/2 접시	외식

✔방문 2 : 24 시간 회상법

- 방문 2 하루 전 식사 (회식 등 평소와 다른 날인 경우 48 시간 전)

식사	식사시간<br>(소요시간)	음식명	재료명	음식량	식사장소
아침					
간식					
점심					
간식					
저녁					
간식					

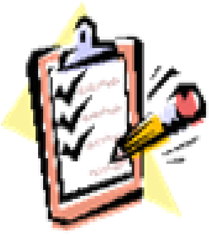

식이조사지(배포용)

**Visit**	☐ 방문 2 ☐ 방문 3 ☐ 추가방문
**방문일**	20 ____년 _____월 _____일
**스크리닝 번호**	Y G **- S** ___ ___ ___<br>기관코드 Screening
**인체적용시험대상자 이니셜**	___ ___ ___

* 연구자께서는 스크리닝 번호, 이니셜, 방문일 등을 기재하여 대상자에게 주십시오

* 매 방문 시 마다 본 **"식이조사지"** 및 **"빈 용기 및 남은 식품"**을 반드시 지참하여 내원하시기 바랍니다.

식사 일지 기록시 유의사항

- 이 식사일지는 식사 섭취량을 조사하기 위한 것입니다.

- 식사일지 작성기간; 다음 방문일 일주일 전 3 일(가능하면 주말 1 일 포함) 동안 평소의 식사와 가장 유사한 날의 식사를 기록해주세요.

(예: 생일식사, 뷔페식사 등은 제외)

- 식사일지 기록 방법

① 식사시간 및 간식시간은 정확하게 기록합니다.

② 음식명을 **주식류**(밥, 빵, 국수 등), **부식류**(국, 찌개 등과 반찬류)의 순서로 빠짐없이 기록합니다.

③ 먹은 **분량**은 **최대한 자세하게** 기록합니다.
잘된 예> 김치 볶음밥 - 보통 밥그릇으로 80% 정도 채워서 1 공기
잘못된 예> 김치 볶음밥 - 1 공기

④ 재료명에는 섭취한 음식의 재료를 빠짐없이 기록합니다.
잘된 예> 김치 볶음밥 - 쌀밥, 배추김치, 후랑크 소시지, 당근, 양파, 계란
잘못된 예> 김치 볶음밥 - 밥, 김치, 계란

⑤ 식사한 장소를 기록합니다.

⑥ **간식도** 식사와 마찬가지로 제품명, 먹은 양 등을 정확하게 기록합니다.

⑦ 식사일지는 평일 2 일, 주말 1 일을 포함하여 다음 방문일 전 1 주일 중 3 일간의 섭취한 음식을 기록하시면 됩니다.
[예시: 2015 년 10 월 13 일(화), 2015 년 10 월 15 일(목), 2015 년 10 월 17 일(토)]

⑧ 식사 직후에 기록합니다.

★ 식사일지

* 작성 예

식사	식사시간<br>(소요시간)	음식명	재료명	음식량	식사장소
아침	7:00 시경<br>(30 분)	토스트<br>쨈<br>계란후라이<br>우유	식빵<br>쨈<br>계란<br>식용유<br>우유	3 장<br>2 찻술<br>1 개<br>1 작은술<br>1 잔(200ml)	식탁
간식	11:00 시경<br>(10 분)	크래커	크래커	3 개(3x4cm)	거실에서<br>TV 보면서
점심	12:00 시경<br>(20 분)	쌀밥<br>된장찌개<br>갈치구이<br>김<br>김치	밥<br>감자<br>호박<br>된장<br>갈치<br>식용유<br>김<br>배추김치	1 공기(210g)<br>1/3 개<br>1/2 개<br>1 큰술<br>중 1 토막<br>1 작은술<br>4 장<br>1/2 접시	외식
간식	3:20 시경<br>(15 분)	찐옥수수	옥수수	1 개	거실에서
저녁	7:00 시경<br>(30 분)	수수밥<br>쇠고기무국<br>감자볶음<br>콩나물무침<br>김치	수수밥<br>쇠고기<br>무<br>소금<br>감자<br>식용유<br>콩나물<br>소금<br>나박김치	2/3 공기<br>2~3 조각(20g)<br>6~7 쪽<br>1/2 작은술<br>1/2 개<br>1 작은술<br>1/4 컵(20g)<br>1/2 작은술<br>1/2 접시	식탁
간식	10:00 시경<br>(5 분)	과일	사과	중 1/2 개	침대에 누워서

*** 작성일 : 1 일(20 년 월 일)**

- 식사 내용을 자세히 기록하여 주세요.

식사	식사시간<br>(소요시간)	음식명	재료명	음식량	식사장소
아침					
간식					
점심					
간식					
저녁					
간식					

*** 작성일 : 2 일(20 년 월 일)**

- 식사 내용을 자세히 기록하여 주세요.

식사	식사시간 (소요시간)	음식명	재료명	음식량	식사장소
아침					
간식					
점심					
간식					
저녁					
간식					

*** 작성일 : 3 일(20 년 월 일)**

- 식사 내용을 자세히 기록하여 주세요.

식사	식사시간<br>(소요시간)	음식명	재료명	음식량	식사장소
아침					
간식					
점심					
간식					
저녁					
간식					

Full Version in Korean

1. Trial Study Protocol for IRB’s Review
2. Case Report Form Designed for Clinical Trial
3. Written Explanation to Participants
4. Consent Form for Participation
5. Consent Form for Human Biologic Materials
6. Survey for Stress Response Inventory (SRI)
7. Survey for Nutrition Intake
8. **Self-Assessment Form**
9. Advertisement for Recruitment

시험대상자 자신에 의한 개선도 평가

시험대상자 이니셜	___ | ___ | ___
스크리닝 번호	YG-S ___ | ___ | ___
평가일	20 년 월 일 (방문 3)

방문1과 비교하여 인체적용시험용 식품 섭취 후 면역력이 증진 되었다고 생각하시나요?	시험대상자 평가
1.매우 우수 : 전반적으로 현저한 개선	
2.우수 : 전반적으로 개선	
3.불변 : 복용전과 차이 없음	
4.악화 : 전반적으로 악화	
5.매우 악화 : 전반적으로 현저한 악화	

작성자	서명:

시험대상자 자신에 의한 개선도 평가

시험대상자 이니셜	___ | ___ | ___
스크리닝 번호	YG-S ___ | ___ | ___
평가일	20 년 월 일 (방문 4)

방문1과 비교하여 인체적용시험용 식품 섭취 후 면역력이 증진 되었다고 생각하시나요?	시험대상자 평가
1.매우 우수 : 전반적으로 현저한 개선	
2.우수 : 전반적으로 개선	
3.불변 : 복용전과 차이 없음	
4.악화 : 전반적으로 악화	
5.매우 악화 : 전반적으로 현저한 악화	

작성자	서명:

Full Version in Korean

1. Trial Study Protocol for IRB's Review
2. Case Report Form Designed for Clinical Trial
3. Written Explanation to Participants
4. Consent Form for Participation
5. Consent Form for Human Biologic Materials
6. Survey for Stress Response Inventory (SRI)
7. Survey for Nutrition Intake
8. Self-Assessment Form
9. **Advertisement for Recruitment**

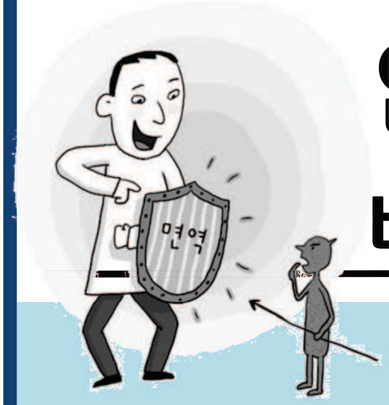

임상 연구 참여자 모집

바이오게르마늄의 면역 증진 효과

연세대학교 식품영양학과에서
바이오게르마늄의 면역증진 효과를 평가하고자
임상 연구를 실시하며, 이에 관심 있는 참여자를 모집합니다.

▶ 참여대상 :
백혈구 수치 4×10^3cells/μℓ ~ 8×10^3cells/μℓ에 해당하는 만 25~75세 남, 여
1년 이내에 2회 이상 감기 증상이 있었던 면역력이 저하된 성인
스트레스 자각 정도가 많은 사람
(선별검사를 통해 기준에 부합하는 자만 참여 가능하며 그 외 제외기준 있음)

▶ 연구장소 : 연세대학교 삼성관 임상영양유전연구실

▶ 참여기간 : 약 8주 (선별 1회, 정규 3회 총 4회 방문)

▶ 섭취방법 :
바이오게르마늄을 1일 2회 (1회 2캡슐) 식후 경구섭취
(무작위배정으로 시험군과 위약군에 1:1의 비율로 배정될 것이며. 위약군에 배정 될 수 있음)

▶ 검사내용:
* 채혈 총 4회 진행(스크리닝방문: 11ml / 0주방문: 18ml / 4주방문: 19ml / 8주방문: 27ml)
1. 기본 혈액검사 및 소변검사
2. 면역지표 검사, 면역 관련 유전자 검사
3. 영양평가 및 상담
* 연구 완료시 소정의 교통비를 지급합니다.

더 자세한 내용은 전화로 문의 바랍니다. (오전 9:30~11:30/ 오후 13:30~18:00)

문의 : ☎ 02-313-8844 (연세대학교 식품영양학과 임상영양유전연구실)
