# Supplementary material for: Immune activation of Bio-Germanium in a randomized, double-blind, placebo-controlled clinical trial with 130 human subjects: Therapeutic opportunities from new insights
Source: PLoS One. 2020 Oct 19;15(10):e0240358. doi: 10.1371/journal.pone.0240358 (PMC7572073; doi:10.1371/journal.pone.0240358)
Supplement: S3 File — (DOC) [file pone.0240358.s003.doc]

**Summary Translation**

**Trial Study Protocol for IRB’s Review**

**Background**

As the worldwide demographics are shifting towards older ages, healthy aging is a defining issue in many countries. Yet, the environmental pollution, work and social stress, and unhealthy lifestyle expose more health risks to geriatric illnesses than before. As such, the topic of enhancing immune function has been continuously attracting researchers' attention, and many new drugs utilizing immunostimulating effects have been developed in the recent years.

Organic germanium has been reported to be biologically active in inducing immunostimulation, anticancer, antitumor, and antiviral activities in addition to various other therapeutic effects. Bio-Germanium, a new type of organic germanium developed in 1990s, is formulated by the biosynthesis of germanium utilizing natural yeast cultivation process.

The aim of this study was to investigate Bio-Germanium supplementation would enhance immune function in healthy individuals. As previous *in-vitro*, *in-vivo* and clinical studies have shown that Bio-Germanium induces the proliferation of B lymphocytes, activation of NK cells, and nitric oxide and TNF-α production as a result of macrophage activation, we focused on further investigating the activities of NK cells and immunocytokines in this clinical trial.

This study will evaluate Bio-Germanium's efficacy on immune function and immune cell activation in healthy volunteers by assessing improvements in certain immune indices (i.e. NK cell activity, white blood cell (WBC) counts, IFN-γ, TNF-α, IgG1, IgG2, IgM, IL-2, IL-6, IL-12).

**Study Purpose**

This study is an 8-week, randomized, double-blind, placebo-controlled clinical trial of Bio-Germanium for the evaluation of efficacy on immune function and immune cell activation.

**Research Location**

Laboratory of Clinical Nutrigenetics/Nutrigenomics

Yonsei University.

Seoul, Korea, Republic of, 03722

**Collaborators**

Fund Support: New Drug Discovery Fund of Geranti Pharmaceutical Ltd.

Contract Research Organization (CRO): NeoNutra Co., Ltd.

**Participating Researchers**

Jong Ho Lee : Department of Food and Nutrition, College of Human Ecology, Yonsei University

Jung Min Cho : Department of Food and Nutrition, College of Human Ecology, Yonsei University

Dong Yeob Shin : Division of Endocrinology and Metabolism, Department of Internal Medicine, Yonsei University College of Medicine

Others : Affiliates from Department of Food and Nutrition, College of Human Ecology, Yonsei University

**Study Duration**

From IRB approval until March 31^st^, 2019

**Study Design**

We designed a randomized, double-blind, placebo-controlled clinical trial for an 8-week supplementation of Bio-Germanium on 130 healthy individuals. The eligibility criteria included healthy male and female volunteers with the age range of 20~75 years and with the leukocyte counts within the 4x10^3^/μL~8x10^3^/μL range.

Principal investigator enrolled the participants and allocated the test materials according to the blinded allocation schedule, which was randomly coded earlier by a third party and strictly controlled for double blind condition. The Bio-Germanium test product and placebo were all in capsules and identical in packaging, appearance, color, texture, and smell.

Subjects were instructed to consume 1,200mg/day (equivalent to 4 capsules) of Bio-Germanium or placebo by taking 2 capsules after breakfast and 2 capsules after dinner. Compliance was assessed by counting the remaining capsules and food records. If the capsules were consumed more than 70%, the compliance was considered fulfilled.

**Inclusion and Exclusion Criteria for Recruitment**

Inclusion Criteria:

1. Healthy male and female volunteers between the ages of 25 to 75 years

2. Screening result for WBC counts in between 4,000 cells/ul and 8,000 cells/ul

3. Volunteers who have agreed to participate in the study and provided a written content by him/herself or through its legal representative

Exclusion Criteria:

1. Those under the treatment for clinically significant acute or chronic diseases in cardiovascular, immune, respiratory, liver, biliary, renal, urinary, nervous, musculoskeletal system as well as psychiatric, infectious, hematologic and neoplastic diseases (exceptions can be made under the discretion of the researcher)

2. Those with uncontrolled hypertension (140/90mmHg or higher, measured after 10 minutes of resting)

3. Those with uncontrolled diabetes (fasting blood glucose levels greater than 126mg/dl or those starting diabetes medication within 3 months)

4. Those received vaccination within 3 months before screening

5. Those with blood AST(GOT) or ALT(GPT) levels greater than 120IU/L

6. Those with blood creatinine level greater than 2.4mg/dL for male and 1.8mg/dL for female

7. Those who have consumed within 2 weeks before screening or are currently consuming health supplements that can affect immune function

8. Those under the severe gastrointestinal symptoms such as heartburn, indigestion, and such

9. Those who are pregnant, breastfeeding or planning to become pregnant during this study

10. Those who are oversensitive or allergic to the investigational product

11. Those who plan to participate in other researches during this study

12. Those who participated in other researches within 4 weeks of the start of this study

13. Those who are deemed inappropriate by the researcher

**Target Size Calculation for Subject Enrollment**

According to the superiority test based on a previous NK cell study, the delta (change) in the NK cell cytotoxic activity at an effector cell:target cell (E:T) ratio of 10:1 was -0.28±10.7% (mean ± standard deviation) in the placebo group and lower than that in the test group (7.4±10.0%). The sample size was then determined *via* a two-sample *t*-test power calculation with an effect size (*d*) of 5.76, a power of 0.8, and a level of significance (*α*) of 0.05. The result indicated that a minimum of 55 subjects per group was needed, and we estimated a dropout ratio of 15%; thus, we selected 65 participants to increase the statistical power of the test for each group, totaling 130 participants for both groups.

**Method for Subject Recruitment**

Utilize poster advertisements.

**Consent from Participants**

The purpose of the study was carefully explained to all subjects, and written informed consent was obtained before participation.

**Primary Outcome Measures**

1) NK cell activity (E:T ratios of 2.5:1, 5:1, 10:1, 50:1)

2) WBC counts

3) IFN- γ

4) IL-2, IL-6, IL-12

5) TNF- α

6) IgG1, IgG2, IgM

7) Self-assessment survey

**Other Materials**

Case report form (CRF), various consent forms, surveys and other materials are shown in Trial Study Protocol's full version in Korean.

**IRB’s Review Result**

**IRB Approval and Registration**

This trial study protocol was approved by the Institutional Review Board of Yonsei University (IRB No. 7001988-201712-HR-322) on December 14^th^ of 2017 according to the Declaration of Helsinki.

The trial information is registered in the below websites.

www.clinicaltrials.gov (NCT03677921)

https://cris.nih.go.kr (KCT0002726)

**Full Version in Korean**

**Table of Contents**

1. **Trial Study Protocol for IRB’s Review**
2. **Case Report Form Designed for Clinical Trial**
3. **Written Explanation to Participants**
4. **Consent Form for Participation**
5. **Consent Form for Human Biologic Materials**
6. **Survey for Stress Response Inventory (SRI)**
7. **Survey for Nutrition Intake**
8. **Self-Assessment Form**
9. **Advertisement for Recruitment**
