# Supplementary material for: Immune activation of Bio-Germanium in a randomized, double-blind, placebo-controlled clinical trial with 130 human subjects: Therapeutic opportunities from new insights
Source: PLoS One. 2020 Oct 19;15(10):e0240358. doi: 10.1371/journal.pone.0240358 (PMC7572073; doi:10.1371/journal.pone.0240358)
Supplement: Attachment — Submitted filename: PO_Response to Reviewers_20200827.docx [file pone.0240358.s005.docx]

**RESPONSES TO REVIEWERS**

**(Reviewer #1)**

August 27^th^, 2020

Dear Reviewer,

We are grateful for your review of our manuscript. In response to your comments, we revised the manuscript accordingly with explanation for your review below.

Thank you again for the opportunity to revise our manuscript, and we hope that our revision meets your expectation and *PLOS ONE*’s standards for publication.

Respectfully,

Jong Ho Lee, Ph.D.

Full Professor, Dept. of Food & Nutrition

College of Human Ecology, Yonsei University

50 Yonsei-ro, Seodaemun-gu, Seoul, 03722, Korea

**============================================================================**

*Note: The line numbers are from the newly submitted, clean version of our Manuscript.*

*The underline represents newly added texts, while the strikethrough represents deleted texts.*

**============================================================================**

1) *The sample size calculation doesn’t match the assumed effect size 5.76. The sample size 55/group will have 80% power to detect an effect size of 0.5 at a two-sided with a significance level (alpha) of 0.050 using a two-sided two-sample equal-variance t-test. If you wanted to test NK cell cytotoxic activity at an effector cell:target cell (E:T) ratio of 10:1 between -0.28±10.7% (mean ± standard deviation) in the placebo group and that in the test group 7.4±10.0%, you only need 30 patients each group. Please provide clear description about the sample size estimate.*

**Authors’ response:**

Thank you for your comment. We updated our manuscript as follows to provide our explanation:

(L267-283)

**Study Design and Determination of Group Size**

An 8-week, randomized, double-blind, placebo-controlled clinical study was conducted with 130 subjects with leukocyte counts of 4-8 (×10^3^/μL) who were divided into two groups: the Bio-Germanium (test) group and the placebo (control) group. Over the eight-week testing period, participants in the test group consumed 1200 mg of Bio-Germanium daily, while those in the control group consumed placebo in the same amount. The sample size was determined and calculated by reference to the NK cell cytotoxic activity results from another clinical trial that showed statistically significant results [61]. According to the superiority test based on that reference study, the delta (change) in the NK cell cytotoxic activity at an effector cell:target cell (E:T) ratio of 10:1 was -0.28±10.7% (mean ± standard deviation) in the placebo group and lower than that in the test group (7.4±10.0%). Based on the difference in deltas between the test and placebo groups (7.68), the application of a 75% adjustment based on the study’s relevance in clinical efficacy yielded an effect size (*d*) of 5.76. The sample size was then determined *via* a two-sample *t*-test power calculation with an effect size (*d*) of 5.76, a power of 0.8, and a level of significance (*α*) of 0.05. The result indicated that a minimum of 55 subjects per group was needed, and we estimated a dropout ratio of 15%; thus, we selected 65 participants to increase the statistical power of the test.

Our step-by-step calculation of the sample size is presented below. The calculation presented below was submitted and provided but unfortunately only in Korean as a supplementary material titled “Trial Study Protocol_Full Version in Korean”. We included this information in the Supporting Information section of our manuscript according to your request.

(Supporting Information: S6)

**Determination of the Group Size and its Calculation**

Our group size was calculated by referring to an earlier clinical trial [61] that showed statistically significant results in NK cell cytotoxic activities. As we set NK cells as our primary biomarker, we used the results from the reference study in the calculation of our sample size.

The values and estimates using the results reported in the reference study are as follows:

| Biomarker | Subject  (n) | | Δ Mean  (baseline to follow-up) | | Standard Deviation (SD) | | Common Standard Deviation (S) |
| --- | --- | --- | --- | --- | --- | --- | --- |
|  | Placebo | Test | Placebo | Test | Placebo | Test |  |
| Cytotoxicity of  NK Cells (10:1) | 31 | 31 | -0.28 | 7.4 | 10.7 | 10.0 | 10.7 |

-	Based on a statistical test power of 80% and significance level of 0.05

-	As the SD of the difference between the test and placebo groups before and after their supplementation was not provided, the correlation coefficient between the before and after supplementation data was estimated to be 0.25

-	A higher SD value of 10.7 was selected and used as S

The following assumptions are applied to estimate the sample size of participants in our trial:

1) Based on the superiority test
2) Significance level (α) of 0.05 using a two-sided test
3) Type 2 error (β) set to 0.2, and the power of the test maintained at 80%
4) A 1:1 allocation ratio in the test and placebo groups
5) Use of data from a clinical trial showing statistically significant NK cell cytotoxicity results. Based on its clinical relevance to our study, a 75% adjustment is applied to the difference in deltas, yielding an effect size (d) of 5.76 (= 7.68 × 75%)

To validate the above, the following hypotheses are set:

H0	:	μt = μc (the mean NK cell activity in the two groups is the same at follow-up)

H1	:	μt ≠ μc (the mean NK cell activity in the two groups is not the same at follow-up)

Assuming 1)-5) above, the sample size calculated for our study is as follows (a two-sided test):

| n_t_ = | (Z_α/2_+Z_β_)^2^ × S^2^ × 2 |
| --- | --- |
|  | d^2^ |

where

Z_α/2_	:	Threshold or fractile that the right tail area is α/2 in a standard normal distribution

Z_β_	:	Threshold or fractile that the right tail area is β in a standard normal distribution

α	:	Size of Type 1 error

β	:	Size of Type 2 error

σ	:	Standard deviation

d	:	Effect size of the difference in Δs between the test and placebo groups following adjustment based on clinical relevance

Using the above, the minimum sample size per group is calculated as 55.

| 55 = | (1.96 + 0.84)^2^ × 10.7^2^ × 2 |
| --- | --- |
|  | 5.76^2^ |

Based on the reference study’s NK cell cytotoxicity data, our sample size per group is set to 55 participants. Assuming a dropout ratio of 15% during the recruiting and screening process, the target sample size of participants for registration is 65 per group, which is equivalent to 130 total participants.

2) *Table 3, Pc-values should not be derived from adjusted baseline ANCOVA for Δ between groups. Please double check. They should be derived from an independent t-test or the Wilcoxon rank-sum test for Δ between groups.*

**Authors’ response:**

We sincerely apologize for our mistake in the footnote. *P*^b^ below in yellow should have been “the Wilcoxon rank sum test” rather than “adjusted baseline ANCOVA”. We suspect that this error could have caused confusion. We revised our manuscript accordingly and also revisited all numbers and footnotes during this process. Thank you for your vigilant review of our work.

To further clarify, we first conducted an independent *t*-test and the Wilcoxon rank sum test based on the normality test results using Kolmogorov Smirnov and Shapiro-Wilk. Then, we conducted an adjusted baseline ANCOVA. Theoretically, we do not need to use the adjusted baseline ANCOVA because we randomly allocated the subjects into our groups.

However, due to the baseline differences between the test and placebo groups, we chose to reconfirm the data using an adjusted baseline ANCOVA. Because our trial revealed statistically significant *P*-values using all methods, we reported the most rigorous results of the adjusted baseline ANCOVA.

Our *P*-values are provided below for your reference. *P*^a^ - *P*^c^ refer to the data originally reported in the manuscript; currently, the data are presented in raw figures before rounding. *P*^d^ is added only to show an independent *t*-test^*^ or the Wilcoxon rank sum test^#^ results we previously calculated and reviewed.

The data represent the mean ± standard deviation ~~error~~.

Δ represents the change from baseline at follow-up.

*P*^a^-values were derived from the Wilcoxon rank sum test at baseline between groups.

*P*^b^-values were derived from the Wilcoxon rank sum test ~~adjusted baseline ANCOVA~~ at follow-up between groups.

*P*^c^-values were derived from adjusted baseline ANCOVA for Δ between groups.

*P*^d^-values derived from an independent *t*-test^*^ or the Wilcoxon rank sum test^#^ for Δ between groups.

**============================================================================**

**RESPONSES TO REVIEWERS**

**(Reviewer #2)**

August 27^th^, 2020

Dear Reviewer,

We are grateful for your review of our manuscript. In response to your comments, we revised the manuscript accordingly and provided an explanation for your review below.

Our team truly enjoyed discussing your intellectually profound and sophisticated comments as they can only come from a distinguished scholar living in pursuit of excellence. Your feedback will remain an exemplary model, especially for the younger members of our team.

Thank you again for the opportunity to revise our manuscript, and we hope that our revision meets your expectation and *PLOS ONE*’s standards for publication.

Respectfully,

Jong Ho Lee, Ph.D.

Full Professor, Dept. of Food & Nutrition

College of Human Ecology, Yonsei University

50 Yonsei-ro, Seodaemun-gu, Seoul, 03722, Korea

**============================================================================**

*Note: The line numbers are from the newly submitted, clean version of our Manuscript.*

*The underline represents newly added texts, while the strikethrough represents deleted texts.*

**============================================================================**

*The manuscript "Immune activation of Bio-Germanium in a randomized, double-blind, placebo-controlled clinical trial with 130 human subjects: therapeutic opportunities from new insights" is interesting, focusing on an interesting point of immune response regulation by organic salts of germanium. There are several articles studying this point, but as the authors report, just a few clinical trials in humans. The idea of studying the natural killer activity as well as humoral immune response is good, but there are some major drawbacks in the study that compromises the results.*

1) *The casuistry is composed of 128 healthy individuals, being 13 males and 115 females. This accounts for a significant difference between the sexes. The females' group were extremely larger than males' group (90% x 10%). It is well known that immune functions differs between sexes, so their particular data might have been discriminated.*

**Authors’ response:**

We screened volunteers recruited from advertisements by the Clinical Nutrigenetics/Nutrigenomics Laboratory at Yonsei University. Although we equally invited both male and female participants aged 20 years to 75 years in the advertisement, we could not control this random nature of sex distribution among the participants. In addition, we did not set any target ratio for female and male participants in our clinical trial protocol, and we could not stop the inflow of female participants. We only started to notice this tendency of more female participants after the midpoint of our recruiting process. At that time, we thought that this trend could reverse at any time and eventually balance out.

In retrospect, our recruiting hours of 9 to 5 during weekdays may have been a factor discouraging the participation of office workers, a population group that tends to include more males in Korea. This population may have a tighter work schedule, especially in the beginning of the year. Therefore, our office hours and particular time of year may have formed an incentivizing factor for the unemployed to participate in our trial. We suspect that this structural limitation could have resulted in more female participation in this particular trial.

After the code open, we examined whether this imbalance between sexes could result in a statistically significant difference. First, the *P*-value derived from an independent *t*-test of the placebo and test groups’ sex distribution was 0.179, suggesting that there was no statistically significant difference between the two groups. Second, we conducted a statistical analysis of the NK cell cytotoxic activities using only the female participants and found that the *P*-values of all E:T ratios were statistically significant, suggesting that there were no contradictory findings between our current analysis results and the female only results. Third, as our protocol was structured as a randomized controlled trial (RCT) following the intention-to-treat (ITT) principle, we could not negligently stop the inflow of female volunteers only to obtain a balanced sex distribution. Thus, we evaluated the effectiveness of supplementation in all subjects using the originally established methodology of the 'per protocol' analysis.

We appreciate your valid comments. According to your request, we included the analysis results presented below in our Supporting Information.

(Supporting Information: S1)

**S1 Table. NK Cell Cytotoxicity and IgG1 Values at Baseline and Follow-up among the Female Subjects.**

The data represent the mean ± standard deviation.

Δ represents the change from baseline at follow-up.

*P*^a^-values were derived from the Wilcoxon rank-sum test at baseline between groups.

*P*^b^-values were derived from the Wilcoxon rank-sum test at follow-up between groups.

*P*^c^-values were derived from adjusted baseline ANCOVA for Δ between groups.

^*^*P*<0.05, ^**^*P*<0.01 and ^***^*P*<0.001 values were derived from a paired *t*-test at follow-up from baseline within groups.

According to your comment, we updated our manuscript as follows:

(L442-448)

When we compared the changes in the two groups (Fig 2), the Bio-Germanium group exhibited NK cell cytotoxicity increases at E:T ratios of 50:1, 10:1, 5:1 and 2.5:1 (12.60±32.91%, 10.19±23.88%, 9.28±16.49% and 7.27±15.28%, respectively), but the placebo group showed decreases (*P*<0.01). Notably, the Bio-Germanium group with an E:T ratio of 2.5:1 exhibited a near twofold increase from 8.91% at baseline to 16.19% at follow-up. The sub-analyses of the female subjects categorized into the pre- and post-menopause groups are provided in the Supporting Information.

As for mentioning this imbalance in our limitations, we added the following according to your request:

(L645-657)

Last but not least, if our study had not been bound by time and resource constraints, more immediate and frequent observations in the earlier stage of the immunocytokine changes, ~~coupled with~~ cytokine secretion assays in response to certain stimuli in ~~on~~ selected immune cells from isolated PBMCs and additional exhaustive observations of NK cells during their isolation from PBMCs would have allowed us to capture a more holistic picture of the immunostimulation mechanism of Bio-Germanium~~,~~ as immunocytokine changes are sometimes instantaneous and short-lived. Also, to better understand the influence of Bio-Germanium on the antibody immune response, further evaluation of the activities of antigen-specific cytotoxic lymphocytes and the responses of antibodies to certain proteins and polysaccharide antigens are imperative to elucidate its mechanism in humoral immunity. Additionally, the use of clinical data from ~~in~~ a single center, the imbalance in the male and female proportion, and the disproportional age distribution could ~~can~~ be ~~a~~ limitations, and caution is needed in generalizing these results to a broader population.

2) *Moreover, as the ages of study groups' were between 20 and 75 years old, it is fundamental to look for reproductive age in females (pre-menopausal x post-menopausal) to evaluate the immunological data (inflammation, NK cytotoxic activity, IgG subclass distribution etc.), as immune function is dependent on hormonal function as well. The immunoendocrine axis is well described nowadays, and immunocytes present receptors for hormones, as well as endocrine system cells respond to immune system agonists and antagonists. so, the data of female subjects might be evaluated according to age and sexual hormonal function.*

**Authors’ response:**

Thank you for raising this very valid issue, and we appreciate your insight.

To further share our findings, as we collected the menopause data of our female participants, we conducted an additional analysis based on your suggestion to separate the female population into two groups based on the pre- and post-menopausal condition. The NK cell cytotoxic activities and IgG1 results were consistent with our current results. According to your suggestion, we included the analysis results presented below in the Supporting Information.

(Supporting Information: S2)

**S2 Table. NK Cell Cytotoxicity and IgG1 Values at Baseline and Follow-up among the Postmenopausal Female Subjects.**

The data represent the mean ± standard deviation.

Δ represents the change from baseline at follow-up.

*P*^a^-values were derived from the Wilcoxon rank-sum test at baseline between groups.

*P*^b^-values were derived from the Wilcoxon rank-sum test at follow-up between groups.

*P*^c^-values were derived from adjusted baseline ANCOVA for Δ between groups.

^*^*P*<0.05 and ^**^*P*<0.01 values were derived from a paired *t*-test at follow-up from baseline within groups.

(Supporting Information: S3)

**S3 Table. NK Cell Cytotoxicity and IgG1 Values at Baseline and Follow-up among the Premenopausal Female Subjects.**

The data represent the mean ± standard deviation.

Δ represents the change from baseline at follow-up.

*P*^a^-values were derived from the Wilcoxon rank-sum test at baseline between groups.

*P*^b^-values were derived from the Wilcoxon rank-sum test at follow-up between groups.

*P*^c^-values were derived from adjusted baseline ANCOVA for Δ between groups.

^*^*P*<0.05 and ^**^*P*<0.01 values were derived from a paired *t*-test at follow-up from baseline within groups.

We agree with your comment that immune function depends on hormonal function. Additionally, you provided profound insight into the interactions between immunocytes and hormones and between endocrine system cells and the immune system. Although we would also like to structure a rigorous trial design, due to resource constraints and the lack of data of the immune response to germanium in humans, we had to position our trial as a newly establishing foundation by exploring the generality of germanium in human immunity.

In our current trial, because we lack hormonal data, we regrettably cannot further investigate the direction you suggested. We attempted to regroup the test group of 65 subjects by sex and age, but due to the small size, the statistical analysis was ineffective.

3) *Another major fault of the study is dependent on the evaluation of humoral immune response. The mere quantification of the immunoglobulin isotypes does not reflect the functionality of antibody responses. It is important to evaluate specific antibody responses to proteins and polysaccharide antigens to evaluate humoral immunity. It is well known that differentiation of B cells leading to the production of diverse isotypes of immunoglobulins depend on the exposition of B cells to different types of antigens in different ecological niches of the immune system (mucosae x systemic immunity, just to cite one example), under different microenvironments, different cytokines etc. Therefore a wider evaluation of humoral immunity is of paramount importance to understand the influence of germanium organic salts in the specific antibody immune response.*

**Authors’ response:**

We agree that a more in-depth analysis of specific antibody responses to proteins and polysaccharide antigens is needed to accurately evaluate the influence of our test material on humoral immunity. Your comments are reflected throughout the manuscript.

As previous studies investigating organic germanium compounds certainly lacked in-depth observations of humoral immune response in humans, it was very difficult for us to determine the depth and breadth of our biomarker selection because we could not anticipate the areas in which our test material would show efficacy. Consequently, we decided that our clinical trial ought to be positioned as a founding milestone for human trials exploring generality, and more targeted and detailed mechanism discoveries are expected to follow.

Although our clinical trial did not include B cells as a biomarker, as Bio-Germanium’s earlier clinical results showed the activation of B cells (CD19), we developed the rationale that Bio-Germanium promotes B cell activation and leads to the secretion of antibodies (IgG1), eventually activating humoral immunity. As B cell activation was observed in an earlier study, we should be more cautious to not be emphatic in our statement.

We edited our manuscript as follows to reflect your comment:

(L174-183)

Bio-Germanium has demonstrated efficacy ~~various efficacies~~ in areas such as promoting immunostimulation, inhibiting tumor progression, and conferring anti-inflammatory effects. In a study by Lee *et al*. [38], Bio-Germanium demonstrated its immunostimulatory effect on humans through the activation of tumor necrosis factor (TNF)-α and B cells (CD19) when compared with the control group ~~(~~*~~P~~*~~<0.05)~~. As TNF-α is involved in the regulation of immune cells, such as in macrophage phagocytosis and T cell homeostasis, and ~~as~~ B cells function in adaptive immunity by secreting antibodies, although more in-depth assessments of the responses of antigen-specific cytotoxic lymphocytes and antibody responses to proteins and polysaccharide antigens are warranted, Bio-Germanium seems to promote stimulation in both humoral and cell-mediated immunity.

(L612-623)

Because ~~With~~ the humoral immunity component is activated by the secretion of antibodies, such as IgG, and the cell-mediated immunity component is activated by antigen-specific cytotoxic lymphocytes, we can suggest further exploration to identify ~~new~~ future opportunities in the application of Bio-Germanium in B cell and T cell combined therapies ~~as a potential application of Bio-Germanium~~. However, as our current study did not focus on investigating the activities of antibodies, more research in this area is warranted in the future. Even though this randomized placebo-controlled study clearly showed that supplementation with Bio-Germanium conferred the immunostimulatory effect, the primary focus of this study was on investigating the working mechanism behind Bio-Germanium's immunostimulation, not on investigating the specificity of effect on a particular disease. To initiate its drug discovery process, further investigation is required in areas such as disease-specific mechanisms and patient-specific effects.

(L645-657)

Last but not least, if our study had not been bound by time and resource constraints, more immediate and frequent observations in the earlier stage of the immunocytokine changes, ~~coupled with~~ cytokine secretion assays in response to certain stimuli in ~~on~~ selected immune cells from isolated PBMCs and additional exhaustive observations of NK cells during their isolation from PBMCs would have allowed us to capture a more holistic picture of the immunostimulation mechanism of Bio-Germanium~~,~~ as immunocytokine changes are sometimes instantaneous and short-lived. Also, to better understand the influence of Bio-Germanium on the antibody immune response, further evaluation of the activities of antigen-specific cytotoxic lymphocytes and the responses of antibodies to certain proteins and polysaccharide antigens are imperative to elucidate its mechanism in humoral immunity. Additionally, the use of clinical data from ~~in~~ a single center, the imbalance in the male and female proportion, and the disproportional age distribution could ~~can~~ be ~~a~~ limitations, and caution is needed in generalizing these results to a broader population.

4) *In the discussion (lines 553-556), the authors tell that the affinity of an antibody to "its antigen" is important to enhance effector functions of NK cells in an antibody dependent cell cytotoxicity (ADCC) mechanism, the observation of increased levels of IgG1 could be directly related to NK stimulation. In my point of view, this conclusion cannot be drawn from your data, as the mere increase of IgG1 do not correlate to the possible activation of NK cells by ADCC.*

**Authors’ response:**

In continuation from your previous comment, we agree that an evaluation of antibody responses to certain proteins and polysaccharide antigens is required to solidify our reasoning. As we previously mentioned, we updated our manuscript accordingly in this respect.

Regarding the correlation between IgG1 and NK cells, although it may be difficult to observe this correlation directly from our results, we developed our reasoning based on the following: as mentioned in our earlier version of manuscript on L573-579, we suspect that the exceptionally unusual weather conditions in Seoul during our trial could be among the reasons for the baseline differences and declining trends observed in certain parameters.

In particular, some immune parameters were decreased at follow-up in both the control and test groups. Our research was carried out from January 9^th^ to June 5^th^, and as most subjects were tested during the winter and spring, the immune-related biomarkers were possibly affected by the cold, windy weather in the winter *(Heo I, Choi Y, Kwon W. The spatial and temporal distributions of NET (Net Effective Temperature) with a function of temperature, humidity and wind speed in Korea. J Geol Soc. 2004;39(1):13-26)* and Asian Dust (AD) outbreaks during the spring. Furthermore, regarding the sudden temperature changes, Brenner et al. reported that cold exposure can contribute to a deterioration in immune functions, including a reduction in the NK cell count *(Brenner I, Castellani J, Gabaree C, Young A, Zamecnik J, Shephard R, et al. Immune changes in humans during cold exposure: effects of prior heating and exercise. J Appl Physiol. 1999;87(2):699-710)*.

In Seoul, the daily average temperature in January and February was below 0 °C (32 °F), and the lowest temperature was -18 °C (0 °F). The temperature abruptly rose to 22 °C (72 °F) in mid-March, dropped back to 0 °C (32 °F) in early April, and then surged to 26 °C (79 °F) in mid-April *(Monthly Weather Report (Jan.-Apr.). Korea Meteorological Administration, 2018. Contract No.: Publication Number: 11-1360000-000002-06)*. Such abrupt undulation of temperature within a short duration was unprecedented from our experience, but it was beyond our capacity to articulate a strong argument regarding weather and immunity.

In addition, AD, originating from the deserts and drylands of Mongolia and China, affects Korea from March to May. One study noted that AD particles can alter immune response profiles *(Honda A, Matsuda Y, Murayama R, Tsuji K, Nishikawa M, Koike E, et al. Effects of Asian sand dust particles on the respiratory and immune system. J Appl Toxicol. 2014;34(3):250-7)*. In reality, AD, fine dust and various pollutants arrive from China mostly in the winter and spring seasons and have already become a diplomatic issue *(https://www.bbc.com/news/world-asia-48346344)*, but again, it was beyond our capacity to investigate the influence of pollutants on immune function. Additionally, due to the diplomatic sensitivity of the topic between Korea and China, we chose to refrain from mentioning “China”.

In summary, we suspect that the severe weather conditions and AD in Seoul had a certain impact on our baseline differences and the declining trends observed in certain parameters in both the placebo and test groups. When we observe a certain deterioration in the placebo group but a flattened trend in the test group, we believe that the test material demonstrates its effect by mitigating potential deterioration.

We agree with your concern, and we removed our earlier statement with an emphatic nuance (L599-611) and expanded our discussion regarding the weather and AD (L624-644).

(L599-611)

Finally, to our knowledge, this study is the first to examine immunoglobulin levels after germanium intervention in human subjects. IgG1 is the major serum immunoglobulin and is principally responsible for the recognition, neutralization, and elimination of pathogens and toxic antigens [94], and the activation of NK cells increases the IgG1 level and facilitates the production of IgG2a antibodies in the *in vivo* setting [95]. As the affinity of an immunoglobulin for an antigen is utilized to enhance immune effector functions [96]~~,~~ and ~~as~~ hypofucosylated IgG1 and IgG3 enhance the effector functions of NK cells [97], the discovery of IgG activation in relation to the activation of ~~and potential subsequent~~ NK cells ~~stimulation~~ by Bio-Germanium in a clinical setting is considered ~~are noted as~~ a novel ~~clinical~~ finding~~s~~ of this study. Additionally, Bio-Germanium’s activation of B cells (CD19) in humans was confirmed earlier by Lee *et al.* [38], which was deemed appropriate as CD19 serves as a signaling intermediary for the stimulation of CD86 on B cells, effecting the subsequent involvement of CD86 in the regulation of the IgG1 levels [98].

(L624-644)

We have also noticed that certain differences in findings from various studies exist due to dissimilarities between experimental tests and clinical trials, including subject selection. Our subjects were healthy adult volunteers between 20 and 75 years of age with normal immune function, while most previous studies were conducted with cancer patients or in *in vivo* settings; thus, the subjects’ physiological and general environmental conditions should also be considered. In particular, some immune parameters were decreased at follow-up in both the control and test groups. O~~o~~ur research was carried out from January 9^th^ to June 5^th^, and as most subjects were tested during the winter and spring, the immune-related parameters were possibly affected by the cold, windy weather in the winter [99] and Asian Dust (AD) outbreaks during the spring in Seoul. Regarding sudden temperature changes, Brenner et al. reported that cold exposure can contribute to deteriorations in immune function, including a reduction in the NK cell count [100]. In Seoul, the daily average temperature in January and February was below 0 °C (32 °F), and the lowest temperature was -18 °C (0 °F). The temperature abruptly increased to 22 °C (72 °F) in mid-March, decreased again to 0 °C (32 °F) in early April, and then surged to 26 °C (79 °F) in mid-April [101]. Such abrupt undulation of temperature within a short duration could be regarded quite extraordinary. In addition, AD, originating from the deserts and drylands of Mongolia, affects Korea from March to May. ~~In addition, o~~One study noted that AD particles can alter immune response profiles [102]. The severe weather conditions and AD in Seoul during the trial period may have had a certain impact on our baseline differences and the declining trends observed in certain parameters in both the placebo and test groups.

5) *There are some points that deserve more discussion. For example, why the placebo group immune function decreased from beginning to ending of experimental period? These alterations are noted in NK cell activity, as well as in IgG1 quantification.*

**Authors’ response:**

We are grateful for your insightful remarks. We also deeply investigated this issue when the results were unblinded.

In contrast to other clinical trials involving subjects under pathological conditions, this trial focused on healthy subjects. From our earlier experience with immunity trials involving healthy subjects (we published more than ten articles since 2013), we often observe similar types of inconsistencies and irregularities in the results. Empirically, we understand that the immune system is very delicate, volatile and easily influenced by external factors, such as the weather, temperature, spring pollen, etc. Even drinking, smoking, exercising or catching a cold on the day before blood collection can alter the results. Thus, we provided the particular weather conditions and Asian dust in Seoul as our previous explanation.

In this trial, we also measured other immunocytokines, although these results are not shown in the report due to statistical insignificance, and the baselines were varied as some were lower while others were higher in the test group. Their trends all differed as some were decreased in test group, while some increased in the placebo group.

We attached the table below for your reference.

The data represent the mean ± standard deviation.

*P*^a^-values derived from an independent *t*-test* or the Wilcoxon rank sum test^#^ at baseline.

*P*^b^-values derived from an independent *t*-test* or the Wilcoxon rank sum test^#^ at follow up between groups.

This issue is always a challenge. Thus, it is very important that we abide by statistical principles and determine the irregularities mathematically. We drafted the manuscript only because the results showed significance based on our rigorous statistical analysis.

As a side note, due to the ethics committee’s heightened awareness of the health and safety of the participants following excessive blood withdrawal, our ethics committee requested that we minimize the volume and frequency of blood withdrawal from what we initially planned, presenting certain predicaments and inflexibilities when defining the depth and breadth of our study in addition to the lack of germanium’s structured clinical trial data regarding human immunity to build upon.

6) *Minor points: The abbreviations presented mainly in page 7 (lines 198-212) must be described before being presented in the text.*

**Authors’ response:**

We appreciate your vigilant review and kind guidance for improving our paper. We reflected your comment in our manuscript.

(L202-225)(KMFDS, Korea Ministry of Food and Drug Safety, is shown in L96)

A number of studies were conducted to determine the organic structure and properties of Bio-Germanium, primarily prepared for the KMFDS approval application for a dietary ingredient with immune enhancement function. The organic properties of Bio-Germanium were analyzed in the following steps: (1) its germanium-binding structure was investigated to determine whether ~~that~~ the germanium in Bio-Germanium is a protein-bound germanium by conducting ~~, for which~~ ion exchange chromatography and gel filtration to compare ~~were used for the comparison of~~ the elution profiles of plain yeast, germanium dioxide and Bio-Germanium, and by conducting sodium dodecyl sulfate-polyacrylamide gel electrophoresis (SDS-PAGE) and N-terminal amino acid sequence analyses ~~were used for the~~ to identify ~~identification of~~ its protein type [29]; (2) the natural formulation technology *via* germanium biosynthesis was assessed to determine whether the inorganic form of germanium is fully transformed into an organic form by conducting~~, for which~~ X-ray diffraction (XRD), nuclear magnetic resonance (NMR) and Fourier transform infrared spectroscopy (FT-IR) analyses ~~were conducted~~ to compare the structural formation of plain yeast, germanium dioxide and Bio-Germanium [30]; (3) the integrity of the organic conversion was investigated to ensure that ~~there is~~ no inorganic germanium is present in Bio-Germanium by following ~~, for which~~ a qualitative analysis protocol utilizing the unique chemical properties of ~~between~~ NaBH_4_ and GeO_2_ reactions ~~was established~~, and NMR, ultraviolet visible spectrophotometry (UV-VIS), inductively coupled plasma atomic emission spectroscopy (ICP-AES), FT-IR and XRD analyses were conducted to detect the presence of inorganic germanium in Bio-Germanium [31]; and~~,~~ (4) the protein-bound germanium structure and its stability were tested to determine ~~for~~ the possibility of dissociation in ~~by~~ gastric juice by applying~~, for which~~ dialysis membrane tubing with a size cut-off of 1200 daltons to ~~was used on~~ Bio-Germanium dissolved in ~~a~~ simulated gastric juice and assessing ~~tested for~~ the presence of inorganic germanium [30]. In conclusion, Bio-Germanium is confirmed to be a protein-bound organic germanium with a stable structure and to not have any inorganic germanium present.

7) *There are several incomplete references, such as ref 21-24, 85-87 and 94-96.*

**Authors’ response:**

We sincerely apologize for the error in our references section. We fixed the problem, and currently, the references are shown in full.

**============================================================================**

**RESPONSES TO REVIEWERS**

**(Reviewer #3)**

August 27^th^, 2020

Dear Reviewer,

We are grateful for your review of our manuscript. In response to your comments, we revised the manuscript accordingly and provided an explanation for your review below.

Our team truly enjoyed discussing your intellectually profound and sophisticated comments as they can only come from a distinguished scholar living in pursuit of excellence. Your feedback will remain an exemplary model, especially for the younger members of our team.

Thank you again for the opportunity to revise our manuscript, and we hope that our revision meets your expectation and *PLOS ONE*’s standard for publication.

Respectfully,

Jong Ho Lee, Ph.D.

Full Professor, Dept. of Food & Nutrition

College of Human Ecology, Yonsei University

50 Yonsei-ro, Seodaemun-gu, Seoul, 03722, Korea

**============================================================================**

*Note: The line numbers are from the newly submitted, clean version of our Manuscript.*

*The underline represents newly added texts, while the strikethrough represents deleted texts.*

**============================================================================**

*The article by Lee* et al*. addresses an important and timely topic, which is to examine changes in immune profiles following Bio-germanium supplementation in a randomized, double-blind, placebo-controlled study. In general, the manuscript is very well written, presenting the data clearly and concisely. However, I have some comments, as follow:*

1) *[The Abstract and the Introduction] The topics were well written. In Line 53, I suggest adding in the sentence that the studies cited were performed in animal model or in cell lines culture.*

**Authors’ response:**

We updated our manuscript accordingly.

(L51-55)

Organic germanium has been reported to be a therapeutic agent with anticancer [3], antitumor [4, 5], antiaging [6, 7], antiviral [8] and anti-inflammatory [9, 10] effects; its anticancer and antiviral effects have been observed *in vivo*, and its antitumor, antiaging and anti-inflammatory effects have been observed both *in vivo* and *in vitro*.

2) *I suggest adding a reference in the sentence from line 90 to 95, or the number of registries in the agencies.*

**Authors’ response:**

As the reviewer suggested, we added the number of each registry as follows:

(L92-98)

In addition, Bio-Germanium is acknowledged as a new dietary ingredient by the US Food and Drug Administration (FDA, ID: FDA-2010-S-0665-0243) and has been approved as a functional ingredient for immune enhancement by the Korea Ministry of Food and Drug Safety (KMFDS, Functional Ingredient No. 2007-15). Furthermore, Bio-Germanium has been granted green list status by the US FDA and is exempted from the import alert of germanium products (FDA Import Alert 54-07).

3) *[Methodology] In Line 240 I believe you would to say: “The fasting whole blood leukocytes…”, right? Correct please.*

**Authors’ response:**

We are grateful for your vigilant review and apologize for this error. We corrected this issue appropriately.

(L247-249)

The fasting whole blood ~~serum~~ leukocyte counts of the volunteers were measured, and 130 participants who met the inclusion criteria were ultimately enrolled.

4) *Regarding the collection of the blood samples, were they collected only before the treatment and after 8 weeks?*

**Authors’ response:**

We updated our manuscript accordingly.

(L319-332)

The p~~P~~articipants were instructed ~~not~~ to not smoke or drink alcohol for at least 30 min before the BP measurement and ~~to~~ fast for 12 h before ~~the initial blood draw and before follow-up visits~~ the blood sample collection, which was conducted at the following check points: screening, week 0, week 4 and the final follow-up visit on week 8. The blood samples were also used to conduct routine checkups and detect any sign of acute conditions. Venous blood specimens were collected in EDTA-coated and plain tubes and were then centrifuged to yield plasma and serum, respectively, which were stored at -70 °C until analysis.

As a side note, due to the ethics committee’s heightened awareness of the health and safety of the participants following excessive blood withdrawal, our ethics committee requested that we minimize the volume and frequency of blood withdrawal from what we initially planned, presenting certain predicaments and inflexibilities when defining the depth and breadth of our study in addition to the lack germanium’s structured clinical trial data regarding human immunity to build upon.

5) *My main doubt in methodology is about the cytotoxicity of NK cells assay. As this test is not performed usually in routine of clinic laboratory, I suggest detailing the technic performed in this assay.*

**Authors’ response:**

We followed the government’s official guidelines for measuring NK cell cytotoxicity outlined in “Guideline for Functional Ingredient Evaluation on Immune Enhancement” *(https://mfds.go.kr, Notification ID: Guide-0779-01)* developed and enforced by the Korea Ministry of Food and Drug Safety (KMFDS), a government body equivalent to the US Food and Drug Administration (FDA). This guideline must be followed to obtain approval from the KMFDS for functional dietary ingredients. Its section on NK cell cytotoxicity assay provides details regarding how isolated PBMCs (E) should be reacted to and cultivated with K562 (T) in various E/T ratios of 5:1, 10:1, etc.

The CytoTox 96® Non-Radioactive Cytotoxicity Assay is a kit manufactured by Promega, a company headquartered in Massachusetts, and we have been using this product since 2013 in more than ten clinical trials performed by our laboratory. We follow the manufacturer’s step-by-step protocol in all our colorimetric cytotoxicity assays. As the assay protocol is provided in extensive details by the manufacturer and is explained by their technicians over the phone and visits, we included only a general overview of how the assay was performed in our earlier manuscript under “Cytotoxic Activity of Natural Killer Cell” to describe the technic performed. According to your request, we expanded this section by providing more details as shown below under a new title - “Preparation for and Execution of the Cytotoxicity Assay”.

(L326-363)

**Preparation for and Execution of the Cytotoxicity Assay**

After the blood collection, to prepare for the isolation of the peripheral blood mononuclear cells (PBMCs) to be used as effector cells, the ~~Isolated peripheral blood mononuclear cells (PBMCs) from whole blood samples were incubated with K562 cells to analyze the cytotoxic activity of NK cells. A~~ whole blood sample was immediately mixed with the same volume of RPMI 1640 medium (Gibco22400-089; Thermo Fisher Scientific, Waltham, Massachusetts, USA), gently overlaid on Histopaque® (1077; Sigma-Aldrich, Irvine, UK) and centrifuged for 20 min at 1800 rpm and 15 °C. After the separation, the buffy coat layer was isolated, washed once with RPMI 1640 medium, and resuspended in 1 mL of 10% FBS medium (fetal bovine serum; Cat.#16000-044; Gibco, Thermo Fisher Scientific, Waltham, Massachusetts, USA) diluted with RPMI 1640. An automated cell counter (LUNA-II; Logos Biosystems, Anyang, Korea) was used to identify and count the live cells among the PBMCs, which completed the effector cell preparation for the assay. Human myeloid leukemia cells (K562; Korean Cell Line Bank, KCLB No. 10243; Korean Cell Line Research Foundation, Seoul, Korea) were used because these cells are highly sensitive and receptive *in vitro* target of NK cells. Bottled and frozen K562 was withdrawn from the liquid nitrogen tank and immediately defrosted in a 37 °C water bath for 10 min. The defrosted K562 was transferred into a conical tube containing 10% FBS medium. The cells were centrifuged, collected, resuspended in fresh FBS medium and distributed into Erlenmeyer flasks ready for cultivation. K562 was cultured in an incubator at 37 °C under 5% CO_2_, and its medium was replaced every 3 days along with regular monitoring to detect any abnormality in morphological changes and cell spheroids. After confirming that K562 reached the log phase, which usually occurred within approximately one week with its peak density approximating 0.75 million/mL, the target cells were centrifuged and collected for the cytotoxicity assay. After completing the preparations of both the effector and target cells for the cytotoxicity assay, t ~~T~~he isolated PBMCs (E) were seeded into 96-well plates with K562 cells (T) at E:T ratios of 50:1, 10:1, 5:1 and 2.5:1 and ~~were then~~ incubated at 37 °C under 5% CO_2_ for at least 4 h. The cytolytic activities of NK cells were analyzed *via* the CytoTox 96® Non-Radioactive Cytotoxicity Assay Kit (G1782; Promega Co., Fitchburg, Massachusetts, USA) according to the manufacturer’s instructions. The color reactions were read at 490 nm using the Victor X5 2030 multilabel plate reader (Victor X5 2030-0050; PerkinElmer, Hopkinton, Massachusetts, USA), and the results were calculated by the following formula:

% Cytotoxicity = (Experimental-Effector Spontaneous-Target Spontaneous) / (Target Maximum-Target Spontaneous) ×100

We adhered to the official guidelines published by KMFDS regarding evaluating NK cell cytotoxicity and the assay kit manufacturer’s instructions regarding using colorimetric methodology. The assays were performed on-site on the same day as the blood collection under randomized and double-blinded conditions, and the obtained assay results were kept blinded until the code open. The PBMCs were used as a whole without the isolation of NK cells and discarded without storage after the analyses were completed.

The kit manufacturer’s introduction to the colorimetric methodology of their product CytoTox 96® Non-Radioactive Cytotoxicity Assay is provided below.

*(https://www.promega.kr/en/products/cell-health-assays/cell-viability-and-cytotoxicity-assays/cytotox-96-non_radioactive-cytotoxicity-assay/?catNum=G1780)*

The CytoTox 96® Non-Radioactive Cytotoxicity Assay is a colorimetric alternative to radioactive cytotoxicity assays. The assay quantitatively measures lactate dehydrogenase (LDH), a stable cytosolic enzyme that is released upon cell lysis, in much the same way as [^51^Cr] is released in radioactive assays. Released LDH in culture supernatants is measured with a 30-minute coupled enzymatic assay that results in the conversion of a tetrazolium salt (INT) into a red formazan product. The amount of color formed is proportional to the number of lysed cells. Visible wavelength absorbance data are collected using a standard 96-well plate reader. The assay can be used to measure membrane integrity for cell-mediated cytotoxicity assays in which a target cell is lysed by an effector cell, or to measure lysis of target cells by bacteria, viruses, proteins, chemicals, etc.

The CytoTox 96® Non-Radioactive Cytotoxicity Assay requires no radioactive waste disposal or [^51^Cr] and eliminates labeling of target cells prior to conducting experiments. Standard equipment can be used to collect absorbance (visible wavelength) data with a standard 96-well plate reader. The assay can be used for a variety of applications including measurement of 1) cell-mediated cytotoxicity; 2) chemical-mediated cytotoxicity; and 3) total cell number. A highly sensitive assay, it can reveal early, low-level damage to cell membranes that is often missed with other methodologies.

6) *[Regarding NK cells assay] Was all the NK assay performed in the same day? Were the assays performed in the same day of blood collection? Were the assays for Placebo and Germanium individuals carried out at the same time (doing a retroactive search after finding out which sample was from which group because of the blind study)?*

*- If the tests have not been carried out on the same day, detail the PBMC storage procedure used before the analysis.*

**Authors’ response:**

Yes, the assays were performed on the same day as the blood collection under randomized, double-blinded conditions. The assay results were first recorded and kept blinded until the code open. The PBMCs were discarded without storage when the NK assays were completed.

We included this information in our manuscript as shown in our response to your earlier question.

7) *The PBMC were added in 96-well plates with K-562 cells. In PBMC, in addition to NK cells, there are different types of lymphocytes and monocytes. Were the monocytes removed prior join to K-562 cells? It seems to me that it would be very interesting to know the proportion of NK cells in the PBMC of each individual. This increase in cytotoxicity could be due to a change in the absolute number of NK cells or maybe effect of other cells, no? Comment.*

*- Even in very careful separations, sometimes residual red blood cells may remain in PBMC. As the assay is based on LDH activity and red blood cells have a lot of LDH, was this considered?*

**Authors’ response:**

We did not separate the NK cells from PBMCs. We used PBMCs as a whole in the cytotoxicity assays. We included this information in our manuscript as shown in our response to your earlier question.

You provided very valid insight, and we greatly appreciate your sophisticated feedback. Ideally, NK cells should be isolated from PBMCs to obtain a more accurate result carving out potential interference from other cells; however, as K562 is primarily prepared and used to test NK cells, due to practicality, PBMCs as a whole are often used, and the results are conventionally accredited to NK cells’ effect. We have conducted NK cell cytotoxicity assays using the same method and technic since 2013 in tandem with the previously mentioned government guidelines for NK cell testing. Additionally, using PBMCs as a whole to test NK cell cytotoxicity on K562 has been widely accepted and reported in other publications.

You also raised the issue of whether the number of NK cells should be counted such that the origin of the increased cytotoxicity can be more accurately identified as either the result of an increase in the number of NK cells or the increased firepower/strength of each NK cell.

We did not count the NK cells as we decided to not distinguish the origin of cytotoxicity as the increased cell counts vs. the increased firepower/strength of each cell for practicality. In general, NK cells represent 10-20% of PBMCs *(Zhang Y, Wallace DL, de Lara CM, et al. In vivo kinetics of human natural killer cells: the effects of ageing and acute and chronic viral infection. Immunology. 2007;121(2):258-265) (Angelo, L.S., Banerjee, P.P., Monaco-Shawver, L. et al. Practical NK cell phenotyping and variability in healthy adults. Immunol Res. 2015;62, 341–356)* and significantly vary in function under the influence of temperature changes, diet, influenza, etc. *(Won, S. J. and Lin, M. T. Thermal stresses reduce natural killer cell cytotoxicity. Journal of Applied Physiology. 1995;79(3), 732-737) (LaVoy, E. C., McFarlin, B. K., and Simpson, R. J. Immune responses to exercising in a cold environment. Wilderness & environmental medicine. 2011;22(4), 343-351) (Barone J, Hebert JR, Reddy MM. Dietary fat and natural-killer-cell activity. Am J Clin Nutr. 1989;50(4):861-867) (Mao H, Tu W, Liu Y, et al. Inhibition of human natural killer cell activity by influenza virions and hemagglutinin. J Virol. 2010;84(9):4148-4157) (Oertelt-Prigione S. The influence of sex and gender on the immune response. Autoimmun Rev. 2012;11(6-7):A479-A485)*; thus, developing a hypothesis to explain the standalone correlation between our test material and the increase in the number of NK cells indeed requires extreme vigilance in controlling the lifestyle of the test subjects and building a large database for a wide spectrum of variables. Currently, although unfortunate, we decided to not include this aspect in our work; however, this topic will be a great area to investigate in the next round of the clinical trial.

The final part of your comment mentions possible interference by red blood cells in our assay. It is true the red blood cells react to LDH; however, from the formula we used shown below, the endogenous LDH release from effector spontaneous is excluded, and then the final cell-mediated % cytotoxicity value is determined. Additionally, we agree with your insight and concerns that red blood cells may have a certain interference; however, as outlined by the KMFDS guideline for immune trials and the kit manufacturer, the interference is deemed tolerable and acceptable.

% Cytotoxicity = (Experimental-Effector Spontaneous-Target Spontaneous) / (Target Maximum-Target Spontaneous) ×100.

According to your request, we included the following in our limitations.

(L645-657)

Last but not least, if our study had not been bound by time and resource constraints, more immediate and frequent observations in the earlier stage of the immunocytokine changes, ~~coupled with~~ cytokine secretion assays in response to certain stimuli in ~~on~~ selected immune cells from isolated PBMCs and additional exhaustive observations of NK cells during their isolation from PBMCs would have allowed us to capture a more holistic picture of the immunostimulation mechanism of Bio-Germanium~~,~~ as immunocytokine changes are sometimes instantaneous and short-lived. Also, to better understand the influence of Bio-Germanium on the antibody immune response, further evaluation of the activities of antigen-specific cytotoxic lymphocytes and the responses of antibodies to certain proteins and polysaccharide antigens are imperative to elucidate its mechanism in humoral immunity. Additionally, the use of clinical data from ~~in~~ a single center, the imbalance in the male and female proportion, and the disproportional age distribution could ~~can~~ be ~~a~~ limitations, and caution is needed in generalizing these results to a broader population.

8) *Concerning K-562 cells, are there some special preparation before use? Is it important the K-562 cells are in log phase before use in the NK-cells assay? Describe in methodology.*

**Authors’ response:**

Thank you very much for noting this issue, and we apologize for missing this issue earlier. We use K562 cells in the log phase. As shown earlier, we updated our manuscript accordingly.

9) *Why this E:T ratio were preferred? Why not an intermediary E:T ratio, like 20, 25 or 30:1?*

**Authors’ response:**

Our ratios of 2.5:1, 5:1, and 10:1 were selected within the assay kit manufacturer's guidelines ranging from 0.625:1 to 10:1. Our selection is also within the boundaries of the previously mentioned government guidelines. Although the kit manufacturer does not provide recommendations or limitations regarding performing assays beyond the 10:1 ratio *(Technical Bulletin: INSTRUCTIONS FOR USE OF PRODUCT of CytoTox 96® Non-Radioactive Cytotoxicity Assay, Promega, USA)*, we chose our 50:1 ratio for exploratory purposes as we often observe that such a ratio is utilized in other publications *(Shen C, Hao SG, Zhao CX, Zhu J, Wang C. Antileukaemia immunity: effect of exosomes against NB4 acute promyelocytic leukaemia cells. J Int Med Res. 2011;39(3):740-747) (Dall'Acqua WF, Kiener PA, Wu H. Properties of human IgG1s engineered for enhanced binding to the neonatal Fc receptor (FcRn). J Biol Chem. 2006;281(33):23514-23524)*

10) *[Results] In general, the results are shown in a clear way. But I have some comments: Why the authors presented some data as mean ± standard deviation and the main results (NK cells and immunoglobulins) in tables and figures as mean ± standard error? Do you think this is the most suitable way? It seems to me that using standard error is not the correct way to present the data. Can you please comment on that?*

**Authors’ response:**

We sincerely apologize for the confusion and inconsistency. We changed all relevant tables to present the standard deviation data. Also, we noticed a mistake in the footnote. *P*^b^ below in yellow should have been “the Wilcoxon rank sum test” rather than “adjusted baseline ANCOVA”. We revised our manuscript accordingly and also revisited all numbers and footnotes during this process.

(L431)

**Table 3. NK Cell Cytotoxicity Values at Baseline and Follow-up.**

The data represent the mean ± standard deviation ~~error~~.

Δ represents the change from baseline at follow-up.

*P*^a^-values were derived from the Wilcoxon rank-sum test at baseline between groups.

*P*^b^-values were derived from the Wilcoxon rank-sum test ~~adjusted baseline ANCOVA~~ at follow-up between groups.

*P*^c^-values were derived from adjusted baseline ANCOVA for Δ between groups.

^*^*P*<0.05, ^**^*P*<0.01 and ^***^*P*<0.001 values were derived from a paired *t*-test at follow-up from baseline within groups.

(L458)

**Table 4. Immunoglobulin G1, G2 and M Values at Baseline and Follow-up.**

The data represent the mean ± standard deviation ~~error~~.

Δ represents the change from baseline at follow-up.

*P*^a^-values were derived from an independent *t*-test (IgG1, normally distributed) or the Wilcoxon rank-sum test (IgG2 and IgM, not normally distributed) at baseline between groups.

*P*^b^-values were derived from the Wilcoxon rank-sum test at follow-up between groups.

*P*^c^-values were derived from an independent *t*-test (IgG1 and IgM, normally distributed) or the Wilcoxon rank-sum test (IgG2, not normally distributed) for Δ between groups.

^*^*P*<0.05 and ^**^*P*<0.01 values were derived from a paired t-test at follow-up from baseline within groups.

11) *Have you any hypothesis to the decreased observed in cytotoxicity and immunoglobulins in placebo group?*

**Authors’ response:**

We also investigated and briefly mentioned this phenomenon in our earlier version of manuscript on L573-579. We suspect that the exceptionally unusual weather conditions in Seoul during our trial could be among the reasons of the baseline differences and declining trends observed in certain parameters.

In particular, some immune parameters were decreased at follow-up in both the control and test groups. Our research was carried out from January 9^th^ to June 5^th^, and as most subjects were tested during the winter and spring, the immune-related biomarkers were possibly affected by the cold, windy weather in the winter *(Heo I, Choi Y, Kwon W. The spatial and temporal distributions of NET (Net Effective Temperature) with a function of temperature, humidity and wind speed in Korea. Journal of the Korean Geographical Society. 2004;39:13-26)* and Asian Dust (AD) outbreaks during the spring. Furthermore, regarding the sudden temperature changes, Brenner et al. reported that cold exposure can contribute to a deterioration in immune functions, including a reduction in the NK cell count *(Brenner I, Castellani J, Gabaree C, Young A, Zamecnik J, Shephard R, et al. Immune changes in humans during cold exposure: effects of prior heating and exercise. Journal of Applied Physiology. 1999;87:699-710)*.

In Seoul, the daily average temperature in January and February was below 0 °C (32 °F), and the lowest temperature was -18 °C (0 °F). The temperature abruptly rose to 22 °C (72 °F) in mid-March, dropped back to 0 °C (32 °F) in early April, and then surged to 26 °C (79 °F) in mid-April *(Monthly Weather Report (Jan.-Apr.). Korea Meteorological Administration, 2018. Contract No.: Publication Number: 11-1360000-000002-06)*. Such abrupt undulation of temperature within a short duration was unprecedented from our experience, but it was beyond our capacity to articulate a strong argument regarding weather and immunity.

In addition, AD, originating from the deserts and drylands of Mongolia and China, affects Korea from March to May. One study noted that AD particles can alter immune response profiles *(Honda A, Matsuda Y, Murayama R, Tsuji K, Nishikawa M, Koike E, et al. Effects of Asian sand dust particles on the respiratory and immune system. Journal of applied toxicology. 2014;34:250-7)*. In reality, AD, fine dust and various pollutants arrive from China mostly in the winter and spring seasons and have already become a diplomatic issue *(https://www.bbc.com/news/world-asia-48346344)*, but again, it was beyond our capacity to investigate the influence of pollutants on immune function. Additionally, due to the diplomatic sensitivity of the topic between Korea and China, we chose to refrain from mentioning “China”.

In summary, we suspect that the severe weather conditions and AD in Seoul had a certain impact on our baseline differences and the declining trends observed in certain parameters in both the placebo and test groups. When we observe a certain deterioration in the placebo group but a flattened trend in the test group, we believe that the test material demonstrates its effect by mitigating potential deterioration.

We agree with your concern, and we expanded our discussion regarding the weather and AD as follows:

(L624-644)

We have also noticed that certain differences in findings from various studies exist due to dissimilarities between experimental tests and clinical trials, including subject selection. Our subjects were healthy adult volunteers between 20 and 75 years of age with normal immune function, while most previous studies were conducted with cancer patients or in *in vivo* settings; thus, the subjects’ physiological and general environmental conditions should also be considered. In particular, some immune parameters were decreased at follow-up in both the control and test groups. O~~o~~ur research was carried out from January 9^th^ to June 5^th^, and as most subjects were tested during the winter and spring, the immune-related parameters were possibly affected by the cold, windy weather in the winter [99] and Asian Dust (AD) outbreaks during the spring in Seoul. Regarding sudden temperature changes, Brenner et al. reported that cold exposure can contribute to deteriorations in immune function, including a reduction in the NK cell count [100]. In Seoul, the daily average temperature in January and February was below 0 °C (32 °F), and the lowest temperature was -18 °C (0 °F). The temperature abruptly increased to 22 °C (72 °F) in mid-March, decreased again to 0 °C (32 °F) in early April, and then surged to 26 °C (79 °F) in mid-April [101]. Such abrupt undulation of temperature within a short duration could be regarded quite extraordinary. In addition, AD, originating from the deserts and drylands of Mongolia, affects Korea from March to May. ~~In addition, o~~One study noted that AD particles can alter immune response profiles [102]. The severe weather conditions and AD in Seoul during the trial period may have had a certain impact on our baseline differences and the declining trends observed in certain parameters in both the placebo and test groups.

As a side note, in contrast to other clinical trials involving subjects under pathological conditions, this trial focused on healthy subjects. From our earlier experience with immunity trials involving healthy subjects (we published more than ten articles since 2013), we often observe similar types of inconsistencies and irregularities in the results. Empirically, we understand that the immune system is very delicate, volatile and easily influenced by external factors, such as the weather, temperature, spring pollen, etc. Even drinking, smoking, exercising or catching a cold on the day before blood collection can alter the results. Thus, we provided the particular weather conditions and Asian dust in Seoul as shown in our earlier explanation.

In this trial, we also measured other immunocytokines, although these results are not shown in the report due to statistical insignificance, and the baselines were varied as some were lower while others were higher in the test group. Their trends all differed as some were decreased in test group, while some increased in the placebo group.

We attached the table below for your reference.

The data represent the mean ± standard deviation.

*P*^a^-values derived from an independent *t*-test* or the Wilcoxon rank sum test^#^ at baseline.

*P*^b^-values derived from an independent *t*-test* or the Wilcoxon rank sum test^#^ at follow up between groups.

This issue is always a challenge. Thus, it is very important that we abide by statistical principles and determine the irregularities mathematically. We drafted the manuscript only because the results showed significance based on our rigorous statistical analysis.

12) *[Discussion] The discussion is ok. The limitations are those appointed in the methodology, regarding NK cells assay. I am not sure if is possible to be so emphatic in your statements.*

*- I suggest remove finding of previous study in the last paragraph and keep the conclusion of this study only.*

**Authors’ response:**

In response to your comments regarding the NK cell assay, we updated the manuscript as follows:

(L645-657)

Last but not least, if our study had not been bound by time and resource constraints, more immediate and frequent observations in the earlier stage of the immunocytokine changes, ~~coupled with~~ cytokine secretion assays in response to certain stimuli in ~~on~~ selected immune cells from isolated PBMCs and additional exhaustive observations of NK cells during their isolation from PBMCs would have allowed us to capture a more holistic picture of the immunostimulation mechanism of Bio-Germanium~~,~~ as immunocytokine changes are sometimes instantaneous and short-lived. Also, to better understand the influence of Bio-Germanium on the antibody immune response, further evaluation of the activities of antigen-specific cytotoxic lymphocytes and the responses of antibodies to certain proteins and polysaccharide antigens are imperative to elucidate its mechanism in humoral immunity. Additionally, the use of clinical data from ~~in~~ a single center, the imbalance in the male and female proportion, and the disproportional age distribution could ~~can~~ be ~~a~~ limitations, and caution is needed in generalizing these results to a broader population.

Regarding our emphatic statements, we edited the text as follows:

(L174-183)

Bio-Germanium has demonstrated efficacy ~~various efficacies~~ in areas such as promoting immunostimulation, inhibiting tumor progression, and conferring anti-inflammatory effects. In a study by Lee *et al*. [38], Bio-Germanium demonstrated its immunostimulatory effect on humans through the activation of tumor necrosis factor (TNF)-α and B cells (CD19) when compared with the control group ~~(~~*~~P~~*~~<0.05)~~. As TNF-α is involved in the regulation of immune cells, such as in macrophage phagocytosis and T cell homeostasis, and ~~as~~ B cells function in adaptive immunity by secreting antibodies, although more in-depth assessments of the responses of antigen-specific cytotoxic lymphocytes and antibody responses to proteins and polysaccharide antigens are warranted, Bio-Germanium seems to promote stimulation in both humoral and cell-mediated immunity.

(L612-623)

Because ~~With~~ the humoral immunity component is activated by the secretion of antibodies, such as IgG, and the cell-mediated immunity component is activated by antigen-specific cytotoxic lymphocytes, we can suggest further exploration to identify ~~new~~ future opportunities in the application of Bio-Germanium in B cell and T cell combined therapies ~~as a potential application of Bio-Germanium~~. However, as our current study did not focus on investigating the activities of antibodies, more research in this area is warranted in the future. Even though this randomized placebo-controlled study clearly showed that supplementation with Bio-Germanium conferred the immunostimulatory effect, the primary focus of this study was on investigating the working mechanism behind Bio-Germanium's immunostimulation, not on investigating the specificity of effect on a particular disease. To initiate its drug discovery process, further investigation is required in areas such as disease-specific mechanisms and patient-specific effects.

(L599-611)

Finally, to our knowledge, this study is the first to examine immunoglobulin levels after germanium intervention in human subjects. IgG1 is the major serum immunoglobulin and is principally responsible for the recognition, neutralization, and elimination of pathogens and toxic antigens [94], and the activation of NK cells increases the IgG1 level and facilitates the production of IgG2a antibodies in the *in vivo* setting [95]. As the affinity of an immunoglobulin for an antigen is utilized to enhance immune effector functions [96]~~,~~ and ~~as~~ hypofucosylated IgG1 and IgG3 enhance the effector functions of NK cells [97], the discovery of IgG activation in relation to the activation of ~~and potential subsequent~~ NK cells ~~stimulation~~ by Bio-Germanium in a clinical setting is considered ~~are noted as~~ a novel ~~clinical~~ finding~~s~~ of this study. Additionally, Bio-Germanium’s activation of B cells (CD19) in humans was confirmed earlier by Lee *et al.* [38], which was deemed appropriate as CD19 serves as a signaling intermediary for the stimulation of CD86 on B cells, effecting the subsequent involvement of CD86 in the regulation of the IgG1 levels [98].

Lastly, regarding your comment to remove the previous study in the last paragraph, we would like to seek your understanding. As shown above (L599-611), B cells are an important component interconnecting NK cells and IgG1. We very much regret having to discard the B cell assay during our biomarker selection process, and in retrospect, we would not have done so if had known earlier that our trial would reveal a significant immunoglobulin result. Although the B cell data are derived from an earlier clinical trial with the same test material and the same design of an 8-week, randomized, double-blind, placebo-controlled trial, we would like to seek your kind reconsideration of this issue as we clearly stated that these data are from “an earlier clinical study” to avoid any confusion. Furthermore, we included a phrase at the end of the sentence separated by a comma structured in a present participial phrase and clearly cited its reference to prevent any confusion. Should you disagree, please do kindly let us know.

(L658-666)

In conclusion, this clinical trial confirms that Bio-Germanium supplementation stimulates immune function by increasing the cytotoxicity of NK cells and activating immunoglobulin**, in addition to** activating B cells and TNF-α, as observed **in an earlier clinical study [38]**. We have added newly discovered clinical findings for germanium's immunostimulation mechanism substantiated through a large-scale trial offering a universal basis by focusing on healthy human subjects, and have contributed to bridging the gap between preclinical and clinical results. We believe Bio-Germanium is a highly promising therapeutic agent and should certainly be further explored for immunotherapy where its immunostimulation effect can have potential development opportunities.

**============================================================================**

**RESPONSES TO REVIEWERS**

**(Reviewer #4)**

August 27^th^, 2020

Dear Reviewer,

We are grateful for your review of our manuscript. In response to your comments, we revised the manuscript accordingly and provided an explanation for your review below.

Our team truly enjoyed discussing your intellectually profound and sophisticated comments as they can only come from a distinguished scholar living in pursuit of excellence. Your feedback will remain an exemplary model, especially for the younger members of our team.

Thank you again for the opportunity to revise our manuscript, and we hope that our revision meets your expectation and *PLOS ONE*’s standards for publication.

Respectfully,

Jong Ho Lee, Ph.D.

Full Professor, Dept. of Food & Nutrition

College of Human Ecology, Yonsei University

50 Yonsei-ro, Seodaemun-gu, Seoul, 03722, Korea

**============================================================================**

*Note: The line numbers are from the newly submitted, clean version of our Manuscript.*

*The underline represents newly added texts, while the strikethrough represents deleted texts.*

**============================================================================**

*In general, the manuscript is well-written, and presents interesting results from what looks to have been a well-run study.*

1) *Please comment on the large sex imbalance in the sample. Only about 10% were male. This is presumably a result of the recruitment method(s) used. Although the sample size is limited, the authors should perform a subset analysis for males and females separately. (An alternative is to perform analysis with sex as a main effect and as an interaction term with treatment in the ANCOVA approach.) This can be reported in a supplemental table, and summarized quite briefly in the manuscript. Please add some remarks about the potential effects of this study imbalance to the discussion of the limitations of the study.*

**Authors’ response:**

We screened volunteers recruited from advertisements by the Clinical Nutrigenetics/Nutrigenomics Laboratory at Yonsei University. Although we equally invited both male and female participants aged 20 years to 75 years in the advertisement, we could not control this random nature of sex distribution among the participants. In addition, we did not set any target ratio for female and male participants in our clinical trial protocol, and we could not stop the inflow of female participants. We only started to notice this tendency of more female participants after the midpoint of our recruiting process. At that time, we thought that this trend could reverse at any time and eventually balance out.

In retrospect, our recruiting hours of 9 to 5 during weekdays may have been a factor discouraging the participation of office workers, a population group that tends to include more males in Korea. This population may have a tighter work schedule, especially in the beginning of the year. Therefore, our office hours and particular time of year may have formed an incentivizing factor for the unemployed to participate in our trial. We suspect that this structural limitation could have resulted in more female participation in this particular trial.

After the code open, we examined whether this imbalance between sexes could result in a statistically significant difference. First, the *P*-value derived from an independent *t*-test of the placebo and test groups’ sex distribution was 0.179, suggesting that there was no statistically significant difference between the two groups. Second, we conducted a statistical analysis of the NK cell cytotoxic activities using only the female participants and found that the *P*-values of all E:T ratios were statistically significant, suggesting that there were no contradictory findings between our current analysis results and the female only results. Third, as our protocol was structured as a randomized controlled trial (RCT) following the intention-to-treat (ITT) principle, we could not negligently stop the inflow of female volunteers only to obtain a balanced sex distribution. Thus, we evaluated the effectiveness of supplementation in all subjects using the originally established methodology of the 'per protocol' analysis.

We appreciate your valid comments. According to your request, we included the analysis results presented below in our Supporting Information.

(Supporting Information: S1)

**S1 Table. NK Cell Cytotoxicity and IgG1 Values at Baseline and Follow-up among the Female Subjects.**

The data represent the mean ± standard deviation.

Δ represents the change from baseline at follow-up.

*P*^a^-values were derived from the Wilcoxon rank-sum test at baseline between groups.

*P*^b^-values were derived from the Wilcoxon rank-sum test at follow-up between groups.

*P*^c^-values were derived from adjusted baseline ANCOVA for Δ between groups.

^*^*P*<0.05, ^**^*P*<0.01 and ^***^*P*<0.001 values were derived from a paired *t*-test at follow-up from baseline within groups.

In addition, we further regrouped the female subjects into the pre- and post-menopausal groups and found that the NK cell cytotoxic activities and IgG1 results were consistent with our current results. These results are also included in the Supporting Information.

(Supporting Information: S2)

**S2 Table. NK Cell Cytotoxicity and IgG1 Values at Baseline and Follow-up among the Postmenopausal Female Subjects.**

The data represent the mean ± standard deviation.

Δ represents the change from baseline at follow-up.

*P*^a^-values were derived from the Wilcoxon rank-sum test at baseline between groups.

*P*^b^-values were derived from the Wilcoxon rank-sum test at follow-up between groups.

*P*^c^-values were derived from adjusted baseline ANCOVA for Δ between groups.

^*^*P*<0.05 and ^**^*P*<0.01 values were derived from a paired *t*-test at follow-up from baseline within groups.

(Supporting Information: S3)

**S3 Table. NK Cell Cytotoxicity and IgG1 Values at Baseline and Follow-up among the Premenopausal Female Subjects.**

The data represent the mean ± standard deviation.

Δ represents the change from baseline at follow-up.

*P*^a^-values were derived from the Wilcoxon rank-sum test at baseline between groups.

*P*^b^-values were derived from the Wilcoxon rank-sum test at follow-up between groups.

*P*^c^-values were derived from adjusted baseline ANCOVA for Δ between groups.

^*^*P*<0.05 and ^**^*P*<0.01 values were derived from a paired *t*-test at follow-up from baseline within groups.

According to your comment, we updated our manuscript as follows:

(L442-448)

When we compared the changes in the two groups (Fig 2), the Bio-Germanium group exhibited NK cell cytotoxicity increases at E:T ratios of 50:1, 10:1, 5:1 and 2.5:1 (12.60±32.91%, 10.19±23.88%, 9.28±16.49% and 7.27±15.28%, respectively), but the placebo group showed decreases (*P*<0.01). Notably, the Bio-Germanium group with an E:T ratio of 2.5:1 exhibited a near twofold increase from 8.91% at baseline to 16.19% at follow-up. The sub-analyses of the female subjects categorized into the pre- and post-menopause groups are provided in the Supporting Information.

As for mentioning this imbalance in our limitations, we added the following according to your request:

(L645-657)

Last but not least, if our study had not been bound by time and resource constraints, more immediate and frequent observations in the earlier stage of the immunocytokine changes, ~~coupled with~~ cytokine secretion assays in response to certain stimuli in ~~on~~ selected immune cells from isolated PBMCs and additional exhaustive observations of NK cells during their isolation from PBMCs would have allowed us to capture a more holistic picture of the immunostimulation mechanism of Bio-Germanium~~,~~ as immunocytokine changes are sometimes instantaneous and short-lived. Also, to better understand the influence of Bio-Germanium on the antibody immune response, further evaluation of the activities of antigen-specific cytotoxic lymphocytes and the responses of antibodies to certain proteins and polysaccharide antigens are imperative to elucidate its mechanism in humoral immunity. Additionally, the use of clinical data from ~~in~~ a single center, the imbalance in the male and female proportion, and the disproportional age distribution could ~~can~~ be ~~a~~ limitations, and caution is needed in generalizing these results to a broader population.

2) *Corn starch was used as placebo. Although it seems likely that this would have no immune effects, this should probably be justified in the manuscript.*

**Authors’ response:**

We previously investigated and found no such research suggesting that corn starch has an immunological effect on humans. If you know otherwise, please kindly let us know. As numerous human studies investigating immune function use corn starch as a placebo *(Komano Y, Shimada K, Naito H, Fukao K, Ishihara Y, Fujii T, et al. Efficacy of heat-killed Lactococcus lactis JCM 5805 on immunity and fatigue during consecutive high intensity exercise in male athletes: a randomized, placebo-controlled, double-blinded trial. J Int Soc Sports Nutr. 2018;15(1):39) (Rautava S, Collado MC, Salminen S, Isolauri E. Probiotics modulate host-microbe interaction in the placenta and fetal gut: a randomized, double-blind, placebo-controlled trial. Neonatology. 2012;102(3):178-84)* and the test drug manufacturer’s previous clinical trial also used corn starch, we chose the same for our clinical trial.

While making the placebo, small amounts of color and flavor additives are added (shown in Table 1) to ensure that the placebo appears and smells identical to the test product. Regarding the other candidates for the placebo material, such as sugar and cellulose, we are told it was very difficult to imitate the color, smell and texture of the test product.

According to your request, we updated our manuscript as follows:

(L304-308)

Those assigned to the test group received Bio-Germanium, and those assigned to the control group received corn starch as placebo. Corn starch was chosen as it is conventionally used as placebo in human trials involving immune experiments [62, 63]. Participants, healthcare providers, investigators, outcome assessors and data analysts were kept blinded throughout the process.

3) *In general, although not strictly necessary, the readability of the tables would probably benefit from rounding at one rather than at two decimal places. The authors may wish to try this out and see.*

**Authors’ response:**

We attempted to use one decimal place, but we decided to show two decimal places as we would like to keep the data more informative and detailed.

4) *In Table 3, there appear to be clear differences between the groups at baseline. Do the authors have any thoughts about why this might be? Could some other covariate potentially explain this difference?*

**Authors’ response:**

We also investigated and briefly mentioned this phenomenon in our earlier version of manuscript on L573-579. We suspect that the exceptionally unusual weather conditions in Seoul during our trial could be among the reasons of the baseline differences and declining trends observed in certain parameters.

In particular, some immune parameters were decreased at follow-up in both the control and test groups. Our research was carried out from January 9^th^ to June 5^th^, and as most subjects were tested during the winter and spring, the immune-related biomarkers were possibly affected by the cold, windy weather in the winter *(Heo I, Choi Y, Kwon W. The spatial and temporal distributions of NET (Net Effective Temperature) with a function of temperature, humidity and wind speed in Korea. Journal of the Korean Geographical Society. 2004;39:13-26)* and Asian Dust (AD) outbreaks during the spring. Furthermore, regarding the sudden temperature changes, Brenner et al. reported that cold exposure can contribute to a deterioration in immune functions, including a reduction in the NK cell count *(Brenner I, Castellani J, Gabaree C, Young A, Zamecnik J, Shephard R, et al. Immune changes in humans during cold exposure: effects of prior heating and exercise. Journal of Applied Physiology. 1999;87:699-710)*.

In Seoul, the daily average temperature in January and February was below 0 °C (32 °F), and the lowest temperature was -18 °C (0 °F). The temperature abruptly rose to 22 °C (72 °F) in mid-March, dropped back to 0 °C (32 °F) in early April, and then surged to 26 °C (79 °F) in mid-April *(Monthly Weather Report (Jan.-Apr.). Korea Meteorological Administration, 2018. Contract No.: Publication Number: 11-1360000-000002-06)*. Such abrupt undulation of temperature within a short duration was unprecedented from our experience, but it was beyond our capacity to articulate a strong argument regarding weather and immunity.

In addition, AD, originating from the deserts and drylands of Mongolia and China, affects Korea from March to May. One study noted that AD particles can alter immune response profiles *(Honda A, Matsuda Y, Murayama R, Tsuji K, Nishikawa M, Koike E, et al. Effects of Asian sand dust particles on the respiratory and immune system. Journal of applied toxicology. 2014;34:250-7)*. In reality, AD, fine dust and various pollutants arrive from China mostly in the winter and spring seasons and have already become a diplomatic issue *(https://www.bbc.com/news/world-asia-48346344)*, but again, it was beyond our capacity to investigate the influence of pollutants on immune function. Additionally, due to the diplomatic sensitivity of the topic between Korea and China, we chose to refrain from mentioning “China”.

In summary, we suspect that the severe weather conditions and AD in Seoul had a certain impact on our baseline differences and the declining trends observed in certain parameters in both the placebo and test groups. When we observe a certain deterioration in the placebo group but a flattened trend in the test group, we believe that the test material demonstrates its effect by mitigating potential deterioration.

We agree with your concern, and we expanded our discussion regarding the weather and AD as follows:

(L624-644)

We have also noticed that certain differences in findings from various studies exist due to dissimilarities between experimental tests and clinical trials, including subject selection. Our subjects were healthy adult volunteers between 20 and 75 years of age with normal immune function, while most previous studies were conducted with cancer patients or in *in vivo* settings; thus, the subjects’ physiological and general environmental conditions should also be considered. In particular, some immune parameters were decreased at follow-up in both the control and test groups. O~~o~~ur research was carried out from January 9^th^ to June 5^th^, and as most subjects were tested during the winter and spring, the immune-related parameters were possibly affected by the cold, windy weather in the winter [99] and Asian Dust (AD) outbreaks during the spring in Seoul. Regarding sudden temperature changes, Brenner et al. reported that cold exposure can contribute to deteriorations in immune function, including a reduction in the NK cell count [100]. In Seoul, the daily average temperature in January and February was below 0 °C (32 °F), and the lowest temperature was -18 °C (0 °F). The temperature abruptly increased to 22 °C (72 °F) in mid-March, decreased again to 0 °C (32 °F) in early April, and then surged to 26 °C (79 °F) in mid-April [101]. Such abrupt undulation of temperature within a short duration could be regarded quite extraordinary. In addition, AD, originating from the deserts and drylands of Mongolia, affects Korea from March to May. ~~In addition, o~~One study noted that AD particles can alter immune response profiles [102]. The severe weather conditions and AD in Seoul during the trial period may have had a certain impact on our baseline differences and the declining trends observed in certain parameters in both the placebo and test groups.

5) *Although nothing needs to be done in the presentation, note that the ANCOVA approach to the "delta" values requires some care in interpretation. That is, the results hold conditional on a given baseline level.*

**Authors’ response:**

We sincerely apologize for our mistake in the footnote. *P*^b^ below in yellow should have been “the Wilcoxon rank sum test” rather than “adjusted baseline ANCOVA”. We revised our manuscript accordingly and also revisited all numbers and footnotes during this process.

To clarify, we first conducted an independent *t*-test and the Wilcoxon rank sum test based on the normality test results using Kolmogorov Smirnov and Shapiro-Wilk. Then, we conducted an adjusted baseline ANCOVA. Theoretically, we do not need to use the adjusted baseline ANCOVA because we randomly allocated the subjects into our groups.

However, due to the baseline differences between the test and placebo groups, we chose to reconfirm the data using an adjusted baseline ANCOVA. Because our trial revealed statistically significant *P*-values using all methods, we reported the most rigorous results of the adjusted baseline ANCOVA.

Our *P*-values are provided below for your reference. *P*^a^ - *P*^c^ refer to the data originally reported in the manuscript; currently, the data are presented in raw figures before rounding. *P*^d^ is added only to show an independent *t*-test^*^ or the Wilcoxon rank sum test^#^ results we previously calculated and reviewed.

The data represent the mean ± standard deviation ~~error~~.

Δ represents the change from baseline at follow-up.

*P*^a^-values were derived from the Wilcoxon rank sum test at baseline between groups.

*P*^b^-values were derived from the Wilcoxon rank sum test ~~adjusted baseline ANCOVA~~ at follow-up between groups.

*P*^c^-values were derived from adjusted baseline ANCOVA for Δ between groups.

*P*^d^-values derived from an independent *t*-test^*^ or the Wilcoxon rank sum test^#^ for Δ between groups.

6) *To help interpret the results in the paper, please include some supplemental figures that display the raw data as "spaghetti" plots. That is, each endpoint is displayed as a set of pre- and post- measurements connected by lines. Treatment groups are separated and juxtaposed.*

**Authors’ response:**

We added your requested graphs in the Supporting Information. We are uncertain of the meaning of “juxtaposed”; thus, we placed the graphs top-and-bottom. Please let us know if placing the line graphs as follows satisfies your request.

**S4 Fig. Line Graphs of NK Cell Cytotoxicity and IgG1 Values at Baseline and Follow-up in the Placebo Group.**

**S5 Fig. Line Graphs of NK Cell Cytotoxicity and IgG1 Values at Baseline and Follow-up in the Bio-Germanium Group.**

7) *In the results section (e.g., Line 408) please report actual p-values rather than "P<0.05").*

**Authors’ response:**

According to your request, we updated our manuscript.

(L457)

(*P*=0.044 ~~<0.05~~, Table 4).

8) *As displayed in Figures 2 and 3, and visible in the manuscript, placebo seems rather active. Can the authors shed some light on this? Could there be a regression to the mean effect at play? Especially in Figure 3, we see that the treatment group essentially shows no change, while the placebo group does show change. It seems very difficult to imply an effect to bio-germanium that seems to only exist in contrast to a marked placebo change from pre to post.*

**Authors’ response:**

We are grateful for your insightful remarks. We also deeply investigated this issue when the results were unblinded.

In contrast to other clinical trials involving subjects under pathological conditions, this trial focused on healthy subjects. From our earlier experience with immunity trials involving healthy subjects (we published more than ten articles since 2013), we often observe similar types of inconsistencies and irregularities in the results. Empirically, we understand that the immune system is very delicate, volatile and easily influenced by external factors, such as the weather, temperature, spring pollen, etc. Even drinking, smoking, exercising or catching a cold on the day before blood collection can alter the results. Thus, we provided the particular weather conditions and Asian dust in Seoul as our previous explanation.

In this trial, we also measured other immunocytokines, although these results are not shown in the report due to statistical insignificance, and the baselines were varied as some were lower while others were higher in the test group. Their trends all differed as some were decreased in test group, while some increased in the placebo group.

We attached the table below for your reference.

The data represent the mean ± standard deviation.

*P*^a^-values derived from an independent *t*-test* or the Wilcoxon rank sum test^#^ at baseline.

*P*^b^-values derived from an independent *t*-test* or the Wilcoxon rank sum test^#^ at follow up between groups.

This issue is always a challenge. Thus, it is very important that we abide by statistical principles and determine the irregularities mathematically. We drafted the manuscript only because the results showed significance based on our rigorous statistical analysis.

As a side note, due to the ethics committee’s heightened awareness of the health and safety of the participants following excessive blood withdrawal, our ethics committee requested that we minimize the volume and frequency of blood withdrawal from what we initially planned, presenting certain predicaments and inflexibilities when defining the depth and breadth of our study in addition to the lack of germanium’s structured clinical trial data regarding human immunity to build upon.

9) *The data do not appear to be available to perform spot checks or to make more informed suggestions.*

**Authors’ response:**

We included more data in the Supporting Information section according to your request. We are more than grateful to have received such insightful feedback and suggestions, allowing our work to be presented in a more comprehensive manner.

10) *In addition, here are some minor suggested edits:*

*Line 80: Change to "clinical study to-date with a group of 130".*

*Line 149: Change to "from yeast by dissolution in either".*

*Line 171: Change to "demonstrated efficacy in areas".*

*Lines 175 and 192: Delete the decorative P-values here in the background section.*

*Lines 227 and 229: Change all occurrences of "a history" to "any history".*

*Line 236: Identify the confidence level associated with "1.23-3.88".*

**Authors’ response:**

We appreciate your vigilant review and kind guidance for improving our paper. We reflected your comment in our manuscript.

(L30-33)

To build a more clinically substantiated foundation for the mechanism underlying ~~behind~~ its immunostimulation, we structured by far the most rigorous clinical study to-date with a ~~larger~~ group of 130 human subjects to examine changes in immune profiles following germanium supplementation.

(L81-83)

For this reason, we structured by far the most rigorous clinical study to-date with a ~~larger~~ group of 130 human subjects to examine changes in immune profiles following 8 weeks of germanium supplementation.

(L149-153)

Previous ~~In~~ *in vitro* studies have confirmed that~~,~~ Bio-Germanium ~~was confirmed to~~ contain~~s~~ only organic germanium by verifying that the germanium in Bio-Germanium is protein-bound organic germanium; the inorganic form of germanium is not present in Bio-Germanium; and the germanium in Bio-Germanium does not dissociate from yeast by dissolution in either gastric juice or water [29-31].

(L174-175)

Bio-Germanium has demonstrated efficacy ~~various efficacies~~ in areas such as promoting immunostimulation, inhibiting tumor progression, and conferring anti-inflammatory effects.

(L176-200)

In a study by Lee *et al.* [38], Bio-Germanium demonstrated its immunostimulatory effect on humans through the activation of tumor necrosis factor (TNF)-α and B cells (CD19) when compared with the control group ~~(~~*~~P~~*~~<0.05)~~. As TNF-α is involved in the regulation of immune cells, such as in macrophage phagocytosis and T cell homeostasis, and ~~as~~ B cells function in adaptive immunity by secreting antibodies, although more in-depth assessments of the responses of antigen-specific cytotoxic lymphocytes and antibody responses to proteins and polysaccharide antigens are warranted, Bio-Germanium seems to promote stimulation in both humoral and cell-mediated immunity. In a test of the anticancer and antitumor activities of Bio-Germanium by Baek *et al.* [2], sarcoma-180 tumor-bearing C57BL/6 mice were fed plain yeast (S. cerevisiae) in the negative control group, doxorubicin (chemotherapy drug) in the positive control group, and Bio-Germanium in the test group. In this study, Bio-Germanium treatment significantly reduced the tumor weight in sarcoma-180 tumor-bearing mice, suggesting that the suppressed progression of tumors may have been the result of increased TNF-α production and effector function of NK cells. Interestingly, while the doxorubicin group showed significant body weight loss, the Bio-Germanium group did not exhibit a weight decrease. In addition to this antitumor effect, the anti-inflammatory effect of Bio-Germanium against paw edema was investigated by Lee et al. [10]. Male Sprague-Dawley rats (180-200 g) were injected with 100 μL of 1% carrageenan to induce paw edema. Ibuprofen (nonsteroidal anti-inflammatory drug) was used as a positive control, and Bio-Germanium was applied in different concentrations as test treatments. Ibuprofen significantly inhibited carrageenan-induced edema, and Bio-Germanium also showed comparable inhibitory effects in a dose-dependent manner ~~(~~*~~P~~*~~<0.05)~~. Further study showed that the anti-inflammatory activity of Bio-Germanium appears to be related to the inhibition of arachidonic acid release and prostaglandin E_2_ (PGE_2_) production in rat basophilic leukemia cells (RBL-2H3).

(L231-236)

The exclusion criteria were as follows: the consumption of drugs or dietary supplements related to immune function within two weeks prior to screening; an allergy to the study products and related substances; hypertension; diabetes; any history/presence of significant metabolic disease; any history/presence of acute or chronic infection of the liver, kidney, or gastrointestinal system; and any history/presence of any other acute or chronic disease requiring treatment.

(L241-244)

Tamakoshi *et al.* reported that the relative risk (RR) of cardiovascular disease mortality in patients with leukocyte counts of 8 to 10 (×10^3^/μL) was more than twofold greater than that in patients with leukocyte counts of 4 to 7.9 (×10^3^/μL) (RR=2.18, 95% confidence interval: 1.23-3.88) [56].

**============================================================================**
